# The Complete Genome Sequence of *Cupriavidus metallidurans* Strain CH34, a Master Survivalist in Harsh and Anthropogenic Environments

**DOI:** 10.1371/journal.pone.0010433

**Published:** 2010-05-05

**Authors:** Paul J. Janssen, Rob Van Houdt, Hugo Moors, Pieter Monsieurs, Nicolas Morin, Arlette Michaux, Mohammed A. Benotmane, Natalie Leys, Tatiana Vallaeys, Alla Lapidus, Sébastien Monchy, Claudine Médigue, Safiyh Taghavi, Sean McCorkle, John Dunn, Daniël van der Lelie, Max Mergeay

**Affiliations:** 1 Molecular and Cellular Biology, Belgian Nuclear Research Center SCK•CEN, Mol, Belgium; 2 Laboratoire Ecosystèmes Lagunaires (ECOLAG), Centre National de la Recherche Scientifique - UMR 5119, Université Montpellier II, Montpellier, France; 3 Joint Genome Institute, Lawrence Berkeley National Laboratory, Walnut Creek, California, United States of America; 4 Laboratoire Microorganismes: Génome et Environnement (LMGE), Centre National de la Recherche Scientifique - UMR 6023, Université Blaise Pascal, Aubière, France; 5 Laboratoire d'Analyses Bioinformatiques pour la Génomique et le Métabolisme (LABGeM), Centre National de la Recherche Scientifique - UMR8030 and CEA/DSV/IG/Genoscope, Evry, France; 6 Biology Department, Brookhaven National Laboratory, Upton, New York, United States of America; University of Hyderabad, India

## Abstract

Many bacteria in the environment have adapted to the presence of toxic heavy metals. Over the last 30 years, this heavy metal tolerance was the subject of extensive research. The bacterium *Cupriavidus metallidurans* strain CH34, originally isolated by us in 1976 from a metal processing factory, is considered a major model organism in this field because it withstands milli-molar range concentrations of over 20 different heavy metal ions. This tolerance is mostly achieved by rapid ion efflux but also by metal-complexation and -reduction. We present here the full genome sequence of strain CH34 and the manual annotation of all its genes. The genome of *C. metallidurans* CH34 is composed of two large circular chromosomes CHR1 and CHR2 of, respectively, 3,928,089 bp and 2,580,084 bp, and two megaplasmids pMOL28 and pMOL30 of, respectively, 171,459 bp and 233,720 bp in size. At least 25 loci for heavy-metal resistance (HMR) are distributed over the four replicons. Approximately 67% of the 6,717 coding sequences (CDSs) present in the CH34 genome could be assigned a putative function, and 9.1% (611 genes) appear to be unique to this strain. One out of five proteins is associated with either transport or transcription while the relay of environmental stimuli is governed by more than 600 signal transduction systems. The CH34 genome is most similar to the genomes of other *Cupriavidus* strains by correspondence between the respective CHR1 replicons but also displays similarity to the genomes of more distantly related species as a result of gene transfer and through the presence of large genomic islands. The presence of at least 57 IS elements and 19 transposons and the ability to take in and express foreign genes indicates a very dynamic and complex genome shaped by evolutionary forces. The genome data show that *C. metallidurans* CH34 is particularly well equipped to live in extreme conditions and anthropogenic environments that are rich in metals.

## Introduction

The abundant metal-containing minerals in our environment have shaped the world we live in. While metals such as calcium or sodium are essential micronutrients sustaining life, other (‘heavy’) metals, such as cobalt, copper, nickel, and zinc are vital cofactors for metallo-proteins and enzymes. At excessive concentrations, these metals may be toxic. Nonessential heavy metals, such as mercury, lead, and cadmium are considered toxic at any concentration: hence they are termed ‘toxic metals’.

Perhaps the largest natural environmental sources of heavy metals are volcanic emissions, forest fires, deep-sea vents, and geysers [1]. Because of geobiochemical transformations and natural routes of accumulation, metal-rich sites are fairly common across the planet. Such sites became colonised by long-lasting microbial consortia that overcame metal toxicity by adapting to metal stress and developing the underlying cellular mechanisms to overcome metal toxicity (such as complexation, efflux, or reduction of metal ions). However, consequent upon industrialisation, the concentrations of heavy and toxic metal in soils further increased worldwide over the past few hundred years [2]. A major contribution to this acceleration is the large-scale burning of fossil fuels that releases a wide range of toxic metals into the environment. Anthropogenic sources of metal contamination also include mining and smelting sites, metal-manufacturing plants, painting- and coating-industries, and tanneries. Other human practices such as applying heavy metals in herbicides and pesticides or using sewage sludge as fertiliser recently raised concern as they have led to the presence of toxic metals in crops, and subsequently, into the foodchain. Typical hot spots of metal contamination are urbanised areas with large industrial activities surrounded by agricultural areas.

The facultative chemolithoautotrophic β-proteobacterium *Cupriavidus* (formerly *Wautersia, Ralstonia, Alcaligenes*) *metallidurans* was isolated in 1976 as a cadmium-resistant and hydrogen-oxidising pseudomonad from a decantation tank at a metal processing factory in Belgium [3]. This strain, later called CH34, was found to be highly resistant to Zn^2+^, Cd^2+^, and Co^2+^; the extrachromosomal genetic determinants conferring this resistance were transferable to related bacteria. Subsequent studies identified multiple loci for metal resistance, many of which were located on two large plasmids pMOL28 and pMOL30 [4]–[14].

Many environmental microbes evolved new or better strategies to survive metal-stressed conditions via the exchange of DNA i.e. by gene transfer. Novick and Roth [15] were among the earliest to report on plasmid-mediated heavy metal resistance when they discovered separate genetic loci on the plasmids of *Staphylococcus aureus* encoding resistance to arsenate, arsenite, antimony, lead, cadmium, zinc, bismuth, and mercury [15]. Since then, the transfer of genes involved in heavy metal resistance (HMR) was documented for many different bacterial species [6],[16]–[18]. For *C. metallidurans* strain CH34, some important observations related to horizontal gene transfer and the acquisition of HMR can be made. First, upon its discovery, strain CH34 was described as a good acceptor of foreign genes in homologous and heterospecific matings that involved the broad host range IncP plasmid RP4::miniMu (aka pULB113) containing a very efficient transposon (prophage miniMu3A). This transposon promotes the generation of R-prime plasmids via an “*in vivo*” cloning process [19], [20] later used to map the largest chromosome of *C. metallidurans* CH34 [21], [22]. Second, strain CH34 is a good recipient of large plasmids that are involved in the HMR or hydrogenotrophy of other *Cupriavidus* strains [23]. And third, strain CH34 served as recipient and donor in soil microcosms to either assess (recombinant) gene dissemination promoted by broad host range plasmids [24]–[26] or to directly capture new broad host range plasmids [27]–[30] and plasmids carrying genes for degradation of xenobiotics [31].

Today, following several taxonomic amendments, strain CH34 is known as the type strain of the species *C. metallidurans* [32]–[34]. This strain tolerates high concentrations of heavy metal ions, including but not necessarily limited to Cu^+^, Cu^2+^, Ni^2+^, Zn^2+^, Co^2+^, Cd^2+^, CrO_4_^2−^, Pb^2+^, Ag^+^, Au^+^, Au^3+^, HAsO_4_^2−^, AsO_2_^−^, Hg^2+^, Cs^+^, Bi^3+^, Tl^+^, SeO_3_^2−^, SeO_4_^2−^, and Sr^2+^, with at least 24 gene clusters distributed over four replicons, i.e., two main chromosomes and two megaplasmids ([8], [10], [14], [35]–[37] and unpublished results). *C. metallidurans* CH34 accomplishes metal detoxification by the concerted action of efflux systems including at least eight P-type ATPases and a wide variety of systems for RND (for Resistance, Nodulation, cell Division) and CDF (for Cation Diffusion Facilitation). It also contains transporters specialized for certain metal ions, whereby efflux may be followed by metal sequestration or complexation (this study; [7], [8], [38]–[40]). Many of these metal responses overlap and are multilayered ([10] and unpublished results). In addition, we have noted changes in gene expression, cell morphology and physiology for *C. metallidurans* CH34 cells in response to spaceflight (i.e. cells grown at the International Space Station during 10-day visits) and oxidative stress [41], [42], as well as simulated microgravity and ionizing radiation (unpublished). The organism has been isolated from very harsh environments such as ultra-clean spacecraft assembly rooms [43] and spent-nuclear-fuel pools [44]. Not surprisingly, genome data indicate that CH34 has at its disposal intricate regulatory networks and many signal transduction systems.

Members of the genus *Cupriavidus* are metabolically diverse and occur in a wide range of habitats. In this study, we compared the CH34 genome with the genomes of three other species: The H_2_-oxidising *C. eutrophus* H16 [45], the chloroaromatic pollutant degrading *C. pinatubonensis* JMP134 (unpublished), and the nitrogen-fixing plant symbiont *C. taiwanensis* [46]. While each of these four species carry plasmids that *de facto* determine their lifestyle, orthology and synteny analyses showed they share many genetic characteristics defined by their bipartite chromosomal structure (denoted as CHR1 and CHR2 throughout this study).

The CH34 genome was also compared with the genomes of more distantly related bacteria belonging to the family *Burkholderiales*. The species included in this analysis were selected on the basis of high overall synteny between their genomes and the CH34 genome, exemplifying their phylogenetic relationship. One striking feature across the analyzed genomes is the overrepresentation of periplasmic receptors containing the Bug domain, a carboxylate-binding motif first noted as overrepresented in host-restricted *Bordetella* species [47]. The prevalence of these receptors in *Cupriavidus* and closely related free-living bacteria may point to a common adaptation strategy to polluted environments rich in metal-carboxylates or carboxylated compounds (i.e., sewage, activated sludge, effluents, and industrial wastewaters), and in rhizospheres affected by anthropogenic pollution or in which carboxylates exuded from plant roots have accumulated.

Here we describe the complete sequencing of *C. metallidurans* strain CH34, and discuss its gene functions derived from manual curation. New insights into its genome structure, its anthology of mobile genetic elements, the many metal-resistance mechanisms, its regulatory networks, vitamin biosynthesis, and siderophore binding and translocation are revealed. Respiration and autotrophy, the oxidation of hydrogen and sulfur, the synthesis of PHB polymeric granules, its metabolic capacities, capsular-protein production, its signal sensory and transduction systems, and its secretion and transport machinery were also considered.

## Results and Discussion

### Genome Structure and General Features

**Figure 1:**
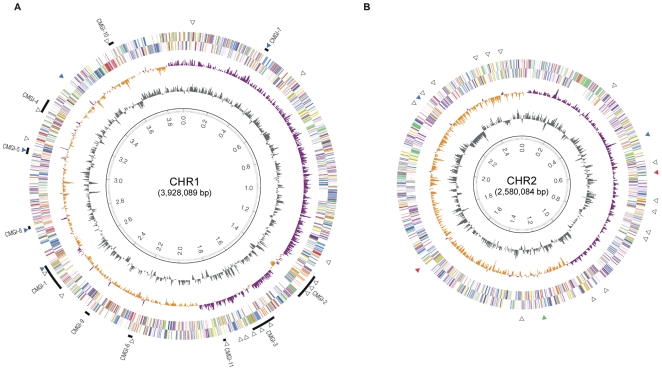
Circular representation of the two main replicons of CH34. Circles display from the inside outwards: (ring 1) scale in Mb, (ring 2) GC-content using a 2 kb window, (ring 3) GC-skew (G-C/G+C ratio) using a 2 kb window, (rings 4 and 5) predicted CDSs transcribed in a counterclockwise/clockwise direction; genomic islands are indicated in black solid bars and given a numbering; black open triangles represent the IS elements; colored triangles depict transposons: red, Tn*6049*; blue, Tn*6049*; and green, Tn*6050*.

The total genome of *C. metallidurans* CH34, of 6,913,352 bp, comprises four circular replicons: Chromosome 1 (CHR1) (3,928,089 bp); chromosome 2 (CHR2) (2,580,084 bp); and two megaplasmids pMOL28 (171,459 bp), and pMOL30 (233,720 bp). Since the two megaplasmids are detailed by Monchy *et al.* [10] and Mergeay *et al.* [7] this report mainly covers the sequencing and expert annotation of the two large replicons. Nonetheless, in a separate subsection below, we give a brief update on the megaplasmids. Table 1 lists the general features of all four replicons. There are four sets of 5S, 16S, and 23S rRNA genes and 12 structural RNA genes that include one RNAaseP RNA (*rnpB*; Rmet_R0004), one tmRNA (*ssrA*; Rmet_R0007), one SRP RNA (*ffs*; Rmet_R0026), and nine riboswitches as predicted by Rfam (http://rfam.sanger.ac.uk/) (Rmet_R0001, Rmet_R0012, Rmet_R0043, Rmet_R0047, Rmet_R0067, Rmet_R0068, Rmet_R0083, Rmet_R0084, and Rmet_R0085). Sixty-two transfer RNA (tRNA) genes with specificities for all 20 amino acids were identified, most located on CHR1. Five tRNA genes are duplicated on CHR2 (two genes once, and three genes twice) (Table 1). Genome analysis using the MaGe system [48] predicts 6,717 coding sequences (CDS). Of those, 4,518 CDS were assigned a function using one of five evidence classes, while 1,274 and 611 CDS remained, respectively, ‘conserved hypothetical’ or ‘hypothetical’. Using the automated COGnitor module [49] imbedded in the MaGe system 5,133 CDS were assigned to one or more COG functional classes (COGs are Clusters of Orthologous Groups of proteins generated by comparing the protein sequences of complete genomes - each cluster contains proteins or groups of paralogs from at least three lineages). (Figure 1; Table 1). The G+C content of the replicons all are very similar to each other, with an average of 63.7% for the large chromosomes, and of 60.3% for the two plasmids (Table 1).

**Table 1:** General features of the *Cupriavidus metallidurans* CH34 genome.

Feature	Chromosome 1	Chromosome 2	Plasmid 1	Plasmid 2
	(CHR1)	(CHR2)	(pMOL28)	(pMOL30)
Size (bp)	3,928,089	2,580,084	171,459	233,720
G + C ratio (mol %)	63.82	63.60	60.50	60.13
tRNA	54	8	0	0
rRNA operons	2	2	0	0
IS elements	30	19	3	5
Transposable elements	8	7	2	2
Genomic Islands	11	0	3	2
Total number of CDSs	3,766	2,493	175	283
Overlapping CDSs	104	92	2	13
CDSs with assigned function	2,784	1,468	100	166
Conserved hypothetical	690	478	54	52
Hypothetical	250	292	15	54
CDSs assigned to COGs	3,045	1,888	77	123
Genes involved in transport	433	288	5	16
Sigma factors	10	7	1	0
*bug* genes	37	87	0	1

**Figure 2:**
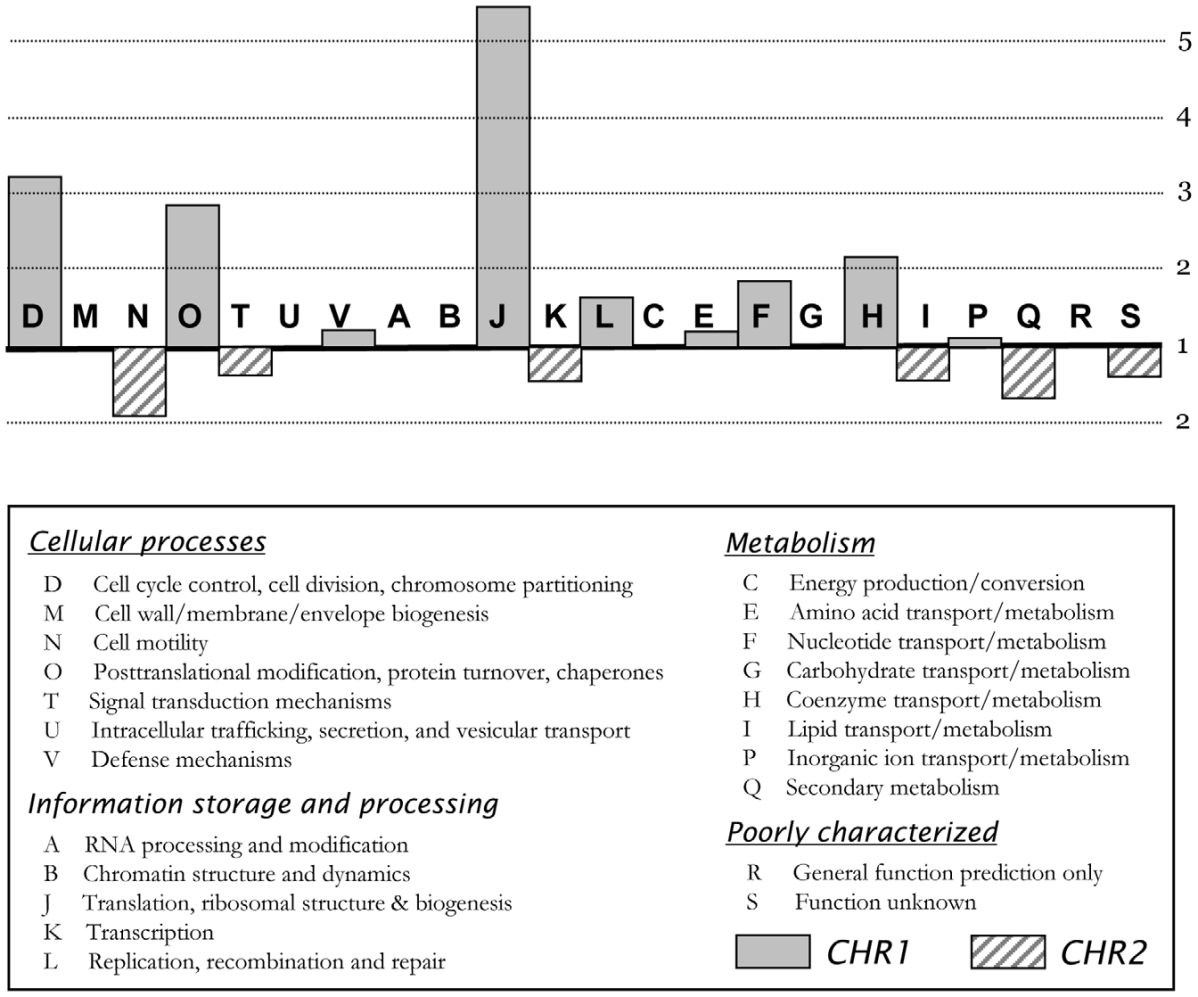
Functional distribution over CHR1 and CHR2 by COG classification. The scale represents the normalised ratio of CDS numbers per replicon (i.e. the ratio of percentages for each class per replicon); a ration of 1 means that genes classified in a particular COG are, numberwise, evenly distributed over both replicons, taking into account the different total count of genes per replicon. Detailed data are in Table S1.

The distribution over the replicons of the genome's functional content also was analyzed using the MaGe system. As expected, the main replicon CHR1 carries most of the essential housekeeping genes, including those needed for DNA replication, DNA repair, cell division, transcription and translation, along with sets of genes required for protein processing, folding, and post-translational modification (Figure 2; Table S1). However, a bias towards particular COG classes was also seen for the smaller replicon (classes I, K, N, Q, and T). These functional classes generally point to adaptation and survival in terms of energy storage and responsive cellular behavior. In addition, the CHR2 replicon contains several genes paralogous to CHR1 genes. Most notably are *dbpA* (Rmet_5284) encoding a second ATP-dependent RNA helicase, a second excinuclease gene *uvrA2* (Rmet_4549), a third copy of an exodeoxyribonuclease gene *xthA3* (Rmet_4910), a second glutamate dehydrogenase gene *gdhA2* (Rmet_5114), a second elongation factor G encoded by *fusA2* (Rmet_5930), a second translation initiation factor IF-1 encoded by *infA2* (Rmet_5176), a second housekeeping sigma-70 factor encoded by *rpoD2* (Rmet_4661) and six genes that code for alternative sigma factors *fliA* (Rmet_3702), *sigJ* (Rmet_3844), *rpoK* (Rmet_4001), *rpoJ* (Rmet_4499), *rpoQ* (Rmet_4686), and *rpoM* (Rmet_5400) - see also dedicated subsection on transcription factors. Furthermore, the CHR2 replicon harbours many genes and gene casettes involved in specialised metabolic- and biosynthetic-activities and adaptive responses, including those involved in carotenoid biosynthesis (*carX*, Rmet_5644; *crtB*, Rmet_4149), acetone utilisation and tolerance (*acxRABC*, Rmet_4104-07), polyhydroxyalkanoic acid (PHA) synthesis and conversion (*phaZ*, *phaZ3*, *phaB3*, *phaC3*), sulfonate/taurine transport and utilisation (*asfRAB*, *tauABC*, *tauD*, *tauE*), sulfite oxidation (*sorAB)*, squalene/hopene synthesis (*sqhC*, Rmet_4148), tannin degradation (*tanA*, Rmet_3841), biofilm formation (*pelABCDEFG*), and exopolysaccharide synthesis (*epsAPB*). It carries most of the genes for motility and chemotaxis (*mot*, *flh*, *fli*, *flg*, *cpa*, *tad*, *pil*, *fim*, and *che* loci), peroxide removal and oxidative stress (*yhjA*, *ahpC*, *ahpD*, *katG*, *pcaCD*, *soxR*, *glrX*), DNA adenine methylation (*dam*), DNA repair (*ada*, *alkA*, *alkB1, alkB2, sbcCD, ogt2, ogt3*), adaptive mutagenesis (*imuA-imuB*-*dnaE2*, Rmet_4128-29-30), and eight copies of the *uspA* gene (two of which are located on transposons) encoding the universal stress protein [most genes and loci without Rmet numbers are also discussed in separate sections]. The CHR2 replicon also carries an intact master two-component phosphorelay system (*bvgSA*) that, in *Bordetella* species, controls hundreds of genes involved in an extensive variety of responses and physiological functions, including virulence, biofilm formation, chemotaxis, and motility [50].

Both chromosomes display clear GC skew transitions that likely correspond with their replication origin and terminus [51] (Figure 1). For CHR1 the GC skew transition region (at point 3.84 on the map, Figure 1A) is about 90 kb away from the typical *dnaA-dnaN-gyrB* layout (genes Rmet_0001, Rmet_0002, and Rmet_0003, with *dnaA* starting at position 104) although the 400 bp upstream region of *dnaA* contains 1 perfect DnaA-binding box (TTATCCACA) and 3 putative DnaA boxes with one or two mismatches to this consensus (Figure S1A). Interestingly, the GC skew transition region located around coordinate 3,837,500 is preceeded immediately by a short region of 213 nt compassing 3 perfect DnaA boxes. Four more putative DnaA boxes (tightly clustered in a 65 nt region and each with one mismatch to the consensus) occur even further upstream of *dnaA*, at coordinates 3,707,588–3,707,653 (Figure S1A). For CHR2 the switch in GC skew is situated around the *repA-parA-parB* genes (Rmet_5817, Rmet_5819, and Rmet_5820) (point 2.43 of the map, Figure 1B) indicating that CHR2 partitioning may be plasmid-like. The *repA* upstream region contains 3 putative DnaA boxes with only one mismatch to the consensus, at locations −392, −419, and −1080 with respect to the startcodon of *repA* (Figure S1B). This region also contains at least 12 repetitive 17 nt-long elements with a highly conserved motif (consensus CGCAGAA(A/T)(C/T)(A/G)GGTACG(C/T)) that may serve as RepA binding sites. In CHR1, the terminus region as predicted by a shift in GC skew is diametrically opposed to the origin of replication (Figure 1)_leading to replichores of approximately equal length. However, the CHR2 replicon is clearly divided in two unequal replichores. The reason for this asymmetry is unclear but may be related to genome dynamics since the *C. metallidurans* CH34 genome harbours numerous Mobile Genetic Elements (MGEs) including many Insertion Sequence (IS) elements, transposons, and Genomic Islands (CMGIs). We further discuss details of the *C. metallidurans* CH34 mobilome in separate subsections.

### Phylogeny and genome comparative analyses

Phylogenetic analyses based on 16S rRNA sequences placed *Cupriavidus metallidurans* CH34 (formerly *Ralstonia* and at one point transferred to *Wautersia*; [34]) firmly into the genus *Cupriavidus*, with its closest relative *C. pauculus* [33], [52]. The complete genome sequences of three other *Cupriavidus* species have been published (Table 2): The pollutant degrading *R. eutropha* strain JMP134 (reclassified as *C. pinatubonensis* by Sato *et al.* [52]); the lithoautotrophic “Knallgass” bacterium *C. necator* strain H16 (with the proposed name *C. eutrophus* H16, Vandamme & Coenye, pers. com.); and the beta-rhizobial nitrogen-fixing plant symbiont *C. taiwanensis*. Throughout this paper we will often refer to these genomes as Cmet (for *C. metallidurans*), Cpin (for *C. pinatubonensis* JMP134), Ceut (for *C. eutrophus* H16) and Ctai (for *C. taiwanensis*).

**Table 2:** Genome organisation of representative species (discussed in this contribution) within the order of *Burkholderiales.*

Organisma,b	Main property	Family	Principle habitat	CHR1	CHR2	Replicon 3 c	Replicon 4	Year/Ref.
C. metallidurans* CH34	heavy metal resistance	*Burkholderiaceae*	heavy metal-rich sediments and soils	3.93	2.58	0.17 (pMOL28)	0.23 (pMOL30)	This report
*C. pinatubonensis* JMP134	degrader of xenobiotics	*Burkholderiaceae*	soil, aquatic; volcanic mudflow deposits	3.81	2.74	0.63 (MPL)	0.09 (pJP4)	unpublished
*C. eutrophus* H16	hydrogen oxidation, PHA^1^ metabolism	*Burkholderiaceae*	soil, freshwater	4.05	2.91	0.45 (pHG1)	-	2006/[45]
*C. taiwanensis*	N_2_-fixing legume symbiont	*Burkholderiaceae*	plant root nodules	3.42	2.50	0.56 (pRALTA)	-	2008/[46]
*R. solanacearum* GMI1000	plant pathogen with wide host range	*Burkholderiaceae*	soil, rhizosphere	3.72	2.09	-	-	2002/[223]
*B. xenovorans* LB400	PCB^2^ degrader	*Burkholderiaceae*	rhizosphere, polluted soils, landfills	4.90	3.36	1.47 (MPL)	-	2006/[224]
D. acidovorans* SPH-1	organotrophy, LAS^3^ degrader	*Comamonadaceae*	soil, aquatic, sewage	6.77	-	-	-	unpublished
B. petrii* Se*-*1111R	degrader of aromatic compounds	*Alcaligenaceae*	river sediments, TCB^4^ contaminated soils	5.29	-	-	-	2008/[148]
*Janthinobacterium sp.*	heavy metal resistance, small sized	*Oxalobacteraceae*	aquatic, 0.22 µm filtered water	4.11	-	-	-	2007/[225]
*H. arsenicoxydans*	arsenic oxidation and scavenging	*Oxalobacteraceae*	activated sludge, industrial waste water	3.42	-	-	-	2007/[226]
*P. necessarius* STIR1	obligate intracellular endosymbiont	*Burkholderiaceae*	freshwater ciliates	1.56	-	-	-	unpublished

a species that are rarely recovered from clinical samples are indicated with an asterisk (*);

b only the propensity for which the species and/or particular strain is best known is given;

c MPL, megaplasmid.

1 PHA, poly(3-hydroxybutyric acid) (cfr. ‘bioplastic’);

2 PCB, polychlorinated biphenyl (cfr. ‘herbicide’);

3 LAS, linear alkylbenzene sulfonate (cfr. ‘laundry surfactant’);

4 TCB, trichlorobenzene.

The genome organisation of these four species is very similar. All four carry two large replicons, that we further nominated as CHR1 and CHR2, with each replicon being of comparable size to their counterparts. The main difference in terms of genome organisation stems from the presence of plasmids (Table 2). Whereas Cmet harbours two megaplasmids of 171- and 233-kb, Ceut and Ctai carry a single megaplasmid, respectively, of 452 kb and 557 kb. The Cpin genome includes a small plasmid of 88 kb (pJP4) along with a megaplasmid of 635 kb.

We determined orthology between all those replicons by analysing the reciprocal best BlastP hits, and then performed cluster analysis using calculated distance scores (see Methods). Our analysis also encompassed representative genomes that, according to the MaGe system, display a well conserved gene order (synteny) to *C. metallidurans* (measuring synteny to the CHR1 replicon). These were *Ralstonia solanacearum* GMI1000 (two large replicons), *Burkholderia xenovorans* LB400 (two large replicons and a plasmid), and *Bordetella petrii* DSM12804, *Delftia acidovorans* SPH-1, *Janthinobacterium* sp. Marseille, and *Herminiimonas arsenicoxydans* (all having a single replicon). We further included *Polynucleobacter necessarius* STIR1 (a single replicon with surprisingly good synteny to Cmet CHR1) to represent, next to the genera *Ralstonia*, *Cupriavidus*, and *Burkholderia*, the fourth genus in the family of *Burkholderiaceae* [53]. Table 2 gives general information on all those species. Lastly, we employed the genome of *Escherichia coli* K-12 in this analysis as a reference and outlier.

**Figure 3:**
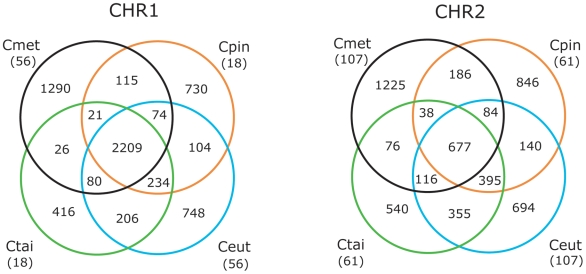
Shared and unique proteins of *Cupriavidus* chromosomes. 4-way comparison Venn diagram illustrating the intersection and differences between chromosomal replicons of completed *Cupriavidus* genomes. Intersections show the number of shared proteins between two or more organisms based on reciprocal best BLAST hits (see Methods). Numbers in parantheses depict the missing overlap sectors due to circular drawing and represent proteins shared between opposite genomes but absent in the other two genomes. Abbreviations: Cmet, *C. metallidurans* CH34; Cpin, *C. pinatubonensis* JMP134; Ceut, *C. eutrophus*; Ctai, *C. taiwanensis*.

Considering only the four *Cupriavidus* species, some interesting observations were made. First, the core number of genes common to all four species clearly is larger for the first replicon with 2,209 genes for CHR1 compared to 677 genes for CHR2 (Figure 3): calculated against the average number of genes present on each replicon, this amounts to 62% and 28%, respectively. This observation, together with a strong bias for essential genes, as we noted for Cmet, and also seen for Ceut and Ctai [45], [46], indicates that CHR1 constitutes the ancestral replicon. The smaller replicon CHR2 probably was acquired as a plasmid during the evolution of *Cupriavidus* and gradually evolved into a second chromosome through genetransfer from either CHR1 or external sources [54]. This idea may explain why CHR2 carries features typical for plasmid-type partitioning, as well as its unique core cellular functions. Here, the delegation of essential genes from the primary replicon to a second replicon would ensure the maintenance of all genes on that replicon, a strategy seemingly common in multipartite prokaryotic genomes [55]. Second, *C. taiwanensis* (Ctai) and *C. eutrophus* H16 (Ceut) have more genes in common (Figure 3) i.e., are more closely related than is the case for the other *Cupriavidus* species, confirming the findings of Amadou *et al.* [46]. And third, both large replicons of Cmet harbour more species-specific genes than do the large replicons of the other *Cupriavidus* genomes (Figure 3).

Additional patterns emerge from the similarity matrix (Figure S3) holding protein-based orthology data for these genomes. As expected, the CHR1 replicons of the four *Cupriavidus* species group tightly together. This is also the case for the *Cupriavidus* CHR2 replicons. For both replicons, the highest orthology is between Ctai and Ceut, and the ranking of the species according to orthology in this cluster remains the same for both replicons, with Cmet displaying the least orthology. There is a clear division among all analysed replicons into two large clusters A and B (Figure S3). Cluster A contains the CHR1 group and the main replicons of *B. xenovorans* and *R. solanacearum*, along with the chromosomes of *D. acidovorans, B. petrii, H. arsenicoxydans*, *Janthinobacterium*, and *P. necessarius* (with the *E. coli* chromosome inside this cluster but clearly an outlier). Cluster B consists of the CHR2 group together with the *C. pinatubonensis*, *B. xenovorans* and *R. solanacearum* megaplasmids and the *B. xenovorans* second chromosome. The grouping of the remaining *Cupriavidus* replicons (all plasmids) from Blast analyses is less clear, although pMOL28 (Cmet), pRALTA (Ctai), and pHG1 (Ceut) appear to be somewhat more related (cluster C) to each other than is the case for pMOL30 (Cmet) and pJP4 (Cpin). Probably, this reflects the fact that the three plasmids in cluster C have the same backbone, i.e., contain the essential genes for plasmid replication and maintenance ([7] and see further). Interestingly, the genome of *P. necessarius* STIR1, an obligate intracellular symbiont of the freshwater ciliate *Euplotes aediculatus*, not only clusters well with the CHR1 group but does so with nearly two thirds (63% on average) of its proteins having an ortholog in any of the other members of this group (in contrast to only 40% of its proteins having an ortholog in the outlier *E. coli*). This high orthology may denote genomic reduction in *P. necessarius* STIR1, in which mainly essential genes were preserved, and is reflected in a significant degree of gene synteny between the *P. necessarius* STIR1 genome and CHR1 of Cmet but much less with CHR2 (Figure S4A). This high synteny with CHR1 was fully supported by MUMmer3 analysis on protein level using the PROmer module (Figure S4B).

**Figure 4:**
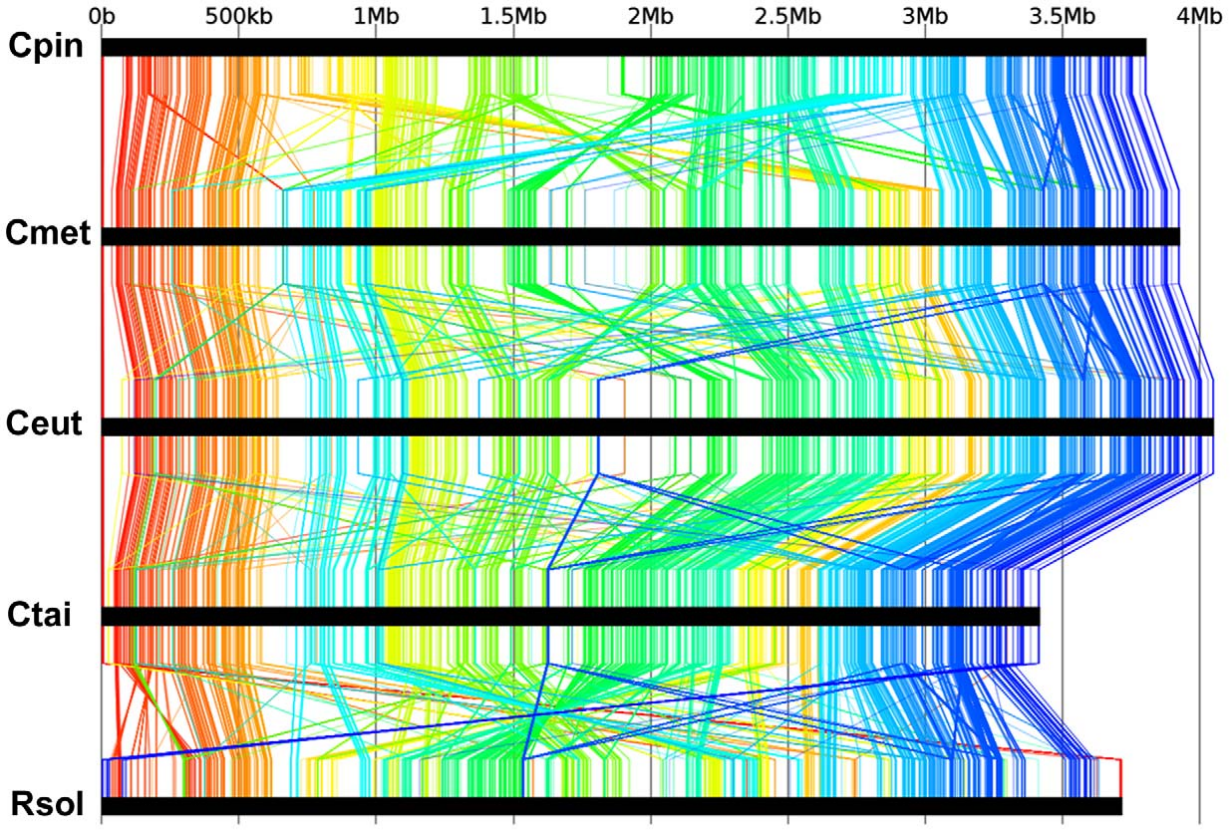
Comparative DNA analysis of *Cupriavidus* chromosomes. Nucleotide based comparison of the CHR1 replicons of the four *Cupriavidus* species (abbreviations as in Figure 3) and *R. solanacearum* GMI1000 (denoted as Rsol) using the anchor-allignment software Murasaki (http://murasaki.dna.bio.keio.ac.jp/) [218]. Scale in Mb is shown on top.

Full length alignment of the main replicons of the four *Cupriavidus* genomes and the closely related *R. solanacearum* genome clearly suggests a common ancestral architecture for CHR1 (Figure 4): overall homology between their respective CHR2 replicons is much lower (Figure S2) probably owing to different evolutionary histories. Such multiple alignments also reveal a large number of DNA rearrangements in the CH34 genome compared to the other analysed genomes (and vice versa), including some large inversions, and multiple sequence duplications causing significant paralogy in both CH34 replicons (discussed further). Pairwise synteny analysis using the LinePlot tool embedded in the MaGe interface fully supported these results (data not shown) while limited synteny was observed between CHR1 of *C. metallidurans* and the single replicons of the other organisms listed in Table 2. The intragenomic synteny between the CHR1 and CHR2 replicons of *C. metallidurans* was not very apparent. However, choosing a small synton size in the LinePlot analysis tool (with S≤5 e.g considering five or less genes for each synteny group), highlighted a myriad of sequence matches (data not shown). This type of local synteny may be partly explained by evolutionary DNA exchange between the two replicons or by parallel insertion of homologous sequences of incoming DNA.

### Paralogy and protein families

Gene duplications (paralogs) are seen as evolutionary events contributing to the innovative drive of organisms in order to adapt to changing environments. We measured paralogy within the *C. metallidurans* genome at the protein level using the GeneRAGE algorithm that facilitates clustering of large protein sequence sets into families according to their sequence similarity [56]. To allow GeneRage to analyse genome parology, we undertook a basic similarity search by an all-to-all BLAST analysis of all *C. metallidurans* CH34 proteins against each other. Smith-Waterman dynamic programming (see Methods) automatically removed ambiguities in the resulting similarity matrix, assuring the method's high accuracy. Multi-domain proteins detected in the genome were disregarded. Half of the remaining proteins (3,311 or 52%) had one or more paralog, while the other half (3,008 or 48%) remained singletons having no match with any of the other proteins in the genome. Remarkably, 1,358 proteins (21%) grouped together into 43 clusters of 10 or more members, while 4 clusters held more then 110 members. These large paralogous families represented proteins involved in two-component signal transduction (n = 138) (predominantly containing GGDEF, EAL, PAS, and HisKA domains), extra-cytoplasmic solute receptors of the tripartite tricarboxylate transporter (TTT) family (n = 123), LysR-type transcriptional regulators (n = 121), and ABC-transporter related proteins (n = 110). Other large families (size>30) comprised proteins involved in membrane structure and transport (membrane fusion proteins, porins, and major facilitator superfamily transporters), or enzymes with general metabolic functions (short-chain dehydrogenases, enoyl-CoA reductases, L-carnitine dehydratases, acyl-CoA dehydrogenases, and acyl-CoA synthetases). These findings are in line with those of Gevers *et al.* [57] who studied paralogy in 106 bacterial genomes, reporting a significant paralog enrichment in functional classes involved in cellular defense mechanisms, metabolism, and transcriptional responses. These authors broadly correlated the number of paralogs in the genomes with the organism's lifestyle, with paranome sizes of 7% for intracellular organisms, such as *Rickettsia conorii*, and up to 41% for free-living highly versatile soil bacteria, such as *Streptomyces coelicolor*. Accordingly, it is not surprising that the survivalist *C. metallidurans* possesses a large, functionally biased paranome. In exploring the smaller clusters holding one- to five-paralogs (656 clusters with a total of 1,724 proteins), it is difficult to discern a functional bias or propose a plausible explanation for paralogy. Many of them contain conserved hypothetical proteins, or proteins similar in function but differing in substrate or cellular roles. Others contain cellular- or transcriptional-regulators specifically directed to heavy-metal resistance (see also Nies *et al.* [58]) or encompass functions related to mobile genetic elements. A quick survey points to several interesting findings. For instance, apparantly, there are no less than six copies of 2-nitropropane dioxygenase that catalyzes the enzymatic reaction 2-nitropropane + oxygen <-> acetone + nitrite. This could be an important finding in view of the known acetone tolerance of *C. metallidurans* CH34 (see further in this discussion), or in terms of converting environmental nitroalkanes for bioremediation purposes. Likewise, there are three copies of butyryl-CoA:acetate-CoA transferase, an enzyme that may be involved in acetate detoxification or instrumental in delivering butyrate to produce polyhydroxybutyrates (PHBs), biopolymers that are stored as an energy reserve in the cell, and which have industrial applications as bioplastics (see further, section on polyester synthesis PHA/PHB). Undoubtedly, it will be exciting to follow these leads but a systematic functional breakdown of these clusters is beyond the scope of this report.

### Mobile genetic elements

A striking feature of the *C. metallidurans* CH34 genome is the presence of at least 16 laterally acquired genomic islands and the abundance of transposase- and recombinase-related gene sequences across the four replicons (Table 1). Using Van Houdt *et al* 's criteria [59], 11 GIs were identified on CHR1 but none on CHR2. Defining GIs on CHR2 is not straightforward since its structure is very different from that of CHR1 and it appears to consist of a complicated patchwork of gene clusters and subclusters generated by multiple integration events with the subsequent retention (and occasional loss) of acquired genes. Further, the CHR2 replicon contains fewer tRNA genes (i.e. only eight compared to 54 in CHR1) and only very few *xerC/D*-like or *int*-like recombinase genes. Previously identified GIs of the plasmids pMOL28 and pMOL30, respectively referred to as CMGI-28a, -28b, -28c, and CMGI-30a and -30b, respectively, all carry heavy-metal response genes; discussed in detail elsewhere [7], [10] and in the next subsection on megaplasmids.

The largest island CMGI-1 is 109 kb and is almost identical (>99.95% DNA sequence similarity) to the PAGI-2 island in *Pseudomonas aeruginosa* clone C, and to the highly conserved PAGI-2C-type islands from many other isolates of this species. It also corresponds to several *C. metallidurans* and *Cupriavidus campinensis* strains isolated from environments polluted with sewage and heavy metals [60], [61]. The observation that clinical strains of nosocomial infections and environmental strains isolated from industrial sites have such a large segment of genetic material in common is worrying since it suggests a recent breach of the natural and artificial barriers between (industrial) environmental reservoirs and clinical environments. Clearly, *P. aeruginosa* has expanded its genomic repertoire to survive in a wide range of different environments, including the human host, in which lateral gene-acquisition might have been enhanced by co-inhabitation and by genetic relatedness [62]. In this respect there is a certain resemblance between the evolutionary forces shaping the genomes of *P. aeruginosa* and *C. metallidurans*, as the latter also acquired and retained many new genetic elements, and consequently new traits, enabling it to survive in many different, often harsh, environments. Moreover, two *C. metallidurans* strains identified by 16S rDNA sequence analysis were recently isolated from the respiratory tract of cystic fibrosis patients [63] warranting concern about the disease-causing potential of *C. metallidurans*.

Three of the *C. metallidurans* GIs (CMGI-2, CMGI-3, and CMGI-4) belong to the Tn*4371* family, first described in strain *C. oxalaticus* A5 (formerly *R. eutropha* A5) [64]–[66]. This family is defined by a quadripartite structure consisting of an integrase module, a region with plasmid/phage/GI maintenance genes, the accessory genes, and the conjugative genes [59] and was recently revisited to describe genomic islands of other environmental bacteria [67].

The 101 kb large CMGI-2 contains at least 25 genes involved in degrading toluene corresponding to CH34's ability to grow with toluene, benzene, or xylene as their sole carbon source (see section on organoautotrophy and degradation of aromatics). Apparantly, additional accessory genes are inserted near this catabolic cluster, including genes involved in chemolithotrophy (e.g. *hyp* and *hox* genes – see section on hydrogenotrophy).

The 97 kb CMGI-3 island also shelters accessory genes in chemolithotrophy organized as two gene cassettes flanked by IS*1071*: one contains the genes for the fixation of CO_2_ (*cbb* genes; described in the section on autotrophic metabolism) and the other encompassing additional genes for hydrogenotrophy (*hyp* and *hox* genes). All the genes allowing *C. metallidurans* to grow aerobically at the expense of CO_2_ and H_2_ are confined to CMGI-2 and CMGI-3 in CHR1: this differs in the closely related H_2_-oxidising chemolithotrophic bacterium *C. eutrophus* H16 wherein the *hox* and *hyp* genes are situated on a 452 kb megaplasmid, pHG1 [45], [68]. It is unclear whether strains H16 and CH34 inherited these homologous sets of *hyp* and *hox* genes from a common ancestor through vertical descent, or whether they were passed on by lateral gene transfer. In contrast, the *cbb* loci (for CO_2_ fixation) in both strains clearly have different origins since the CH34 *cbb* genes are phylogenetically much more closely related to the *cbb* homologues of photosynthetic- and nitrifying-organisms [45].

The 56 kb CMGI-4 island has a mosaic structure and lacks the conjugative module. Instead, its extremity carries genes involved in the biosynthesis of methionine, and the degradation of phosphonates; the transposon Tn*6048* also occurs there. The DNA sequence of the CMGI-4 island is highly similar to a region in the *D. acidovorans* SPH-1 genome [69]–[71] except that the latter does not contain Tn*6048* but carries a complete Tn*4371*-like structure flanked by Tn*6051* [59].

The contribution of the remaining GIs (between 11- and 24-kb in size) towards the particular lifestyle of *C. metallidurans* is unclear. Seemingly, CMGI-5 is composed of plasmid remnants, while CMGI-7 might be involved in resistance to arsenic. The CMG-11 island harbours a putative fimbrial operon of the chaperone-usher-dependent assembly pathway. The islands CMGI-6 and CMGI-8 are cryptic islands holding copies of IS*1087* and Tn*6049*, while CMGI-9 and CMGI-10 consist almost entirely out of hypothetical genes.

The *C. metallidurans* CH34 genome accommodates 19 transposons of five different types [59] (Table 3). Two ‘mercury transposons’ Tn*4378* and Tn*4380* (both of the Tn*501* family) are located, respectively, on pMOL28 and pMOL30, and were previously reported [10]. We identified three novel types of transposon: Tn*6048* (10.4 kbp), containing 8 genes of unknown function but highly induced by zinc and lead, Tn*6049* (3.5 kbp), often associated with genomic islands, and Tn*6050* (6.8 kbp) found twice in CHR2 in opposite orientations and separated by 143 kb. Remarkably, all 12 copies of Tn*6049* carry the same ATPase-encoding gene, putatively named *gspA* because its product shows strong similarities to putative general secretion pathway ATPases. We also note that although the core region (*tnpA*-*tnpR*) of Tn*6050* is very similar to that of the mercury transposons Tn*4378* and Tn*4380*, its accessory gene cassette is uninvolved in mercury resistance and encodes proteins that are only rarely associated with transposons, viz., a sulfate permease, a universal stress protein (UspA), and a DksA-like DnaK suppressor protein. We reason that there is a functional linkage between these genes, as this module, in full or part, also occurs on other Tn*3*-related transposons i.e., alone, as in Tn*1013* from the IncP-1α plasmid pBS228 [72], or in combination with antibiotic resistance determinants, as inTn*6001,* Tn*1403*, and Tn1404* transposons detected in clinical- and environmental-strains of *Pseudomonas* [73], [74]. Closer examination of the chromosomal regions flanking the Tn*6050* transposons in strain CH34 suggests the possibility of chromosomal inversion through homologous recombination, thereby effecting several loci involved in metal resistance [75], [76].

**Table 3:** Distribution of transposons in *C. metallidurans* CH34.

Element	Total nr.	CHR1	CHR2	pMOL28	pMOL30
Tn*4378*	1			1	
Tn*4380*	1				1
Tn*6048*	3	1	2		
Tn*6049*	12	7	3	1	1
Tn*6050*	2		2		

Significantly, the *C. metallidurans* CH34 genome bears no fewer than 57 insertion elements evenly distributed over the four replicons (Table 4). The most abundant are IS*Rme3* (an IS*3* family member), with 10 copies, and IS*1088* (an IS*30* family member), with nine copies. Conceivably, many of those 57 IS elements have played an important role in modulating the architecture of the CH34 genome and in the continuous acquisition of new traits. For instance, the incorporation of *hyp* and *hox* genes into CMGI-3 likely results from active IS*1071* elements, while in contrast, the ability of strain CH34 to grow autotrophically may be lost owing to IS*1071*-mediated excision. Similarly, the two IS*Rme5* copies on CMGI-2 are located amidst multiple transposase genes and fragments, and probably were the driving force toward integrating the *hyp/hox* cluster on CMGI-2. In addition, both copies of IS*Rme7* flank the genomic island CMGI-11 while two copies of IS*Rme3* are associated with the genomic islands in pMOL30, further strengthening our belief that IS elements principally shaped the CH34 genome.

**Table 4:** Distribution of IS elements in *C. metallidurans* CH34.

Element	Family (sub-)	Total nr.	CHR1	CHR2	pMOL28	pMOL30
IS*1071*	Tn*3*	3	3			
IS*1086*	IS*30*	3	1	1	1	
IS*1087*	IS*3* (IS*2*)	2	2			
IS*1088*	IS*30*	9	3	6		
IS*1090*	IS*256*	4	4			
IS*Rme1*	IS*5* (IS*427*)	4	2	2		
IS*Rme3*	IS*3* (IS*3*)	10	3	5		2
IS*Rme4*	IS*21*	2	2			
IS*Rme5*	IS*481*	4	3	1		
IS*Rme6*	IS*5* (IS*427*)	1	1			
IS*Rme7*	IS*6*	2	2			
IS*Rme8*	IS*4*	2	1	1		
IS*Rme9*	IS*21*	1			1	
IS*Rme10*	IS*30*	1				1
IS*Rme11*	IS*3*	2		2		
IS*Rme12*	IS*3* (IS*150*)	1	1			
IS*Rme15*	IS*3* (IS*51*)	2		1		1
IS*Rme17*	IS*3* (IS*150*)	1	1			
IS*Rme18*	Tn*3*	1			1	
IS*Rme19*	IS*66*	1				1
IS*Rme20*	IS*21*	1	1			

We also detected in the CH34 genome a series of putative mobile elements that previously were unnoticed (detailed by Van Houdt *et al.* [59]). The first group of such elements contain tyrosine-based site-specific recombinase genes consecutively placed in series of three. We observed such trios in a wide variety of α- and β-proteobacteria and termed them *r*ecombinase *i*n *t*rio or *rit* elements. One trio (*ritA_2_B_2_C_2_*) (Rmet_1239 to Rmet_1241) belongs to the integrase module of CMGI-2 and apparently inactivates a putative DNA repair gene, *radC*. This trio is highly similar to the *B. petrii rit* element (Bpet1498 to Bpet1500) of genomic island GI3, having corresponding aa (amino acid) sequence identities of 77% or higher. Intriguingly, the *B. petrii rit* element inactivates a *radC* gene (Bpet1497), and all genes in both *rit* elements display ATGA overlaps strongly suggesting translational coupling. A second trio (*ritA_1_B_1_C_1_*) (Rmet_1271 to Rmet_1273) also lies on CMGI-2 and is part of a complex accessory module; it inactivates a *tnpA* gene of the IS*66* family. The *C. metallidurans ritA_1_B_1_C_1_* element exhibits high similarity to the *rit* elements on plasmid pHG1 of *C. eutrophus*, showing very high aa sequence similarities (>92% identity) for *ritA_1_* and *ritB_1_* to either PHG129 and PHG141, or either PHG130 and PHG140, respectively. Further, there are high aa sequence similarities (40–54% identity) between *ritA_1_B_1_C_1_* and the trio PHG134/135/136. Immediately adjacent to this maze of pHG1 recombinase genes we discerned an IS*66* type *tnpA* gene (PHG142) but we are uncertain whether it is interrupted or not. We also noticed four-gene elements, comprising three recombinases close to each other and invariably accompanied by a fourth gene encoding a transcriptional regulator of the TetR family [59]. The last group of mobile elements we list here is the *bimAB* module, named after the *bimA* recombinase and *bimB* putative repressor genes first noted on the pMOL28 and pMOL30 plasmids as genepairs Rmet_6192/93 (linked to a *chr* locus) and Rmet_5987/88 (linked to a *czc* locus), respectively [7]. The latter module (21 genes) is fully conserved in *Ralstonia picketti* 12J, indicating a horizontal transfer event. The *bimAB* element has also been associated to HMR loci in other bacteria [59].

An interesting feature of *C. metallidurans* strain CH34, and characteristic for related metallotolerant *C. metallidurans* strains, is the increased activity of transposable elements when cells are grown at elevated temperature i.e. 37°C instead of 30°C. This phenomenon is referred to as temperature induced mutagenesis in which survivor cells appear to have undergone DNA arrangements, insertions, or deletions [6], [22], [77], [78]. One well studied case is the integration of a 44 kb DNA fragment in the pMOL28 plasmid and the identification of IS*1086* particpating in this event [77], [79]. The resulting plasmid pMOL50 has a much higher conjugation frequency then either pMOL28 or pMOL30 and is able to very efficiently (retro)mobilise very large DNA fragments from the CH34 chromosome (of up to 1 Mb), a property that was exploited to construct the genetic map of the CH34 main chromosome, CHR1 [22].

### A brief update on megaplasmids in strain CH34

Both megaplasmids pMOL28 and pMOL30 carry key genes for resistance to heavy metals [9]. Plasmid pMOL28 encodes resistance to nickel and cobalt [80], [81], chromate [5], mercury [4], [10], and thallium [82], [83]. Plasmid pMOL30 specifies resistance to zinc, cadmium, and cobalt [13], [84], copper [75], silver [8], and lead [37], [85]. The corresponding gene clusters on pMOL28 and pMOL30, and the replication region of pMOL28 were identified subsequently (reviewed by [7], [8], [10]). Monchy *et al.* [10] derived detailed genetic maps of both megaplasmids from their full DNA sequence. This distinguished the genes involved in plasmid maintenance, replication, and partition, together constituting the ‘backbone’ of the plasmid, next to specialized ‘accessory’ genes that group together on genomic islands i.e., CMGI-28a, -28b, -28c, and CMGI-30a and -30b. In pMOL28 and pMOL30, these GIs contain all the genes involved in metal resistance [7], [59]. Notably, the pMOL28 replicon belongs to a family of megaplasmids that share a similar backbone and include the megaplasmids pHG1 (452 kb) of *C. eutrophus* H16 and pRALTA (557 kb) of *C. taiwanensis*. Owing to the distinctly specialized functions on pMOL28 (heavy metal resistance), pHG1 (hydrogenotrophy and chemolithotrophy), and pRALTA (nitrogen fixation and legume symbiosis), these megaplasmids might well be regarded as the decisive factor in fixing their hosts to their ecological niche. A direct relationship of the megaplasmid pMOL30 to other megaplasmids is less certain. Nonetheless, through synteny analysis and advanced annotation using the MaGe platform we noted that the backbone of pMOL30 shares, over a region of approximately 60 kb, many characteristics with the pBVIE01 megaplasmid (398 kb) of *B. vietnamiensis* G4 [7].

Despite detailed annotation of megaplasmids pMOL28 and pMOL30 [10], continuous manual curation of the CH34 genome via updated databases, and earlier functional information from proteomic- and transcriptomic-approaches [5], [75], [83], [86] many enigmatic genes remain in both plasmids (Table 1).

Recent transcriptomic analyses revealed that five such genes, located on CMGI-28a, are induced by submillimolar concentrations of chromate (no data given). All five genes are sited immediately downstream of the chromate-regulated operon *chrIA_1_B_1_CEF_1_* (Rmet_6199 through Rmet_6204): we called them *chrO* (Rmet_6198), *chrN* (Rmet_6197), *chrP* (Rmet_6196, encoding an MFS family permease with ten transmembrane α-helical segments), *chrY* (Rmet_6195, encoding a putative membrane protein), and *chrZ* (Rmet_6194) (Table 5). Synteny analysis using MaGe shows a fair to good resemblance between the pMOL28 *chr* locus of *C. metallidurans* and the *chr* loci of *Methylobacterium* sp. 4–46, *B. vietnamiensis* G4, and *B. pseudomalei*. However, the *chr* locus in *Methylobacterium* sp. 4–46 lacks *chrE*, *chrN*, and *chrP* orthologues, the four *chr* loci of *B. vietnamiensis* G4 all lack *chrP* and *chrZ* orthologues, and the majority of *chr* loci across 21 *B. pseudomallei* strains lack *chrC*, *chrE*, *chrO*, and *chrN* orthologues. This puzzling variation in *chr* locus structure may point to differences among these bacteria in terms of selectivity to chromate or another metal-oxyanion.

**Table 5:** Metal detoxification in *C. metallidurans* CH34 occurs via a variety of export systems encoded by 24 HMR gene clusters on 4 replicons.

Type^a^	Replicon	Gene cluster^b^	Rmet_ number	Metal(s)	GI/transposon^c^	Mechanism^d^	Fc^e^	Refs.
**partially plasmid-redundant**	CHR1	***cdfX**pbrR_2_cadA pbrC_2_*, *pbrR_3_* (§)	2299, 2302–2304, 3456	Pb^2+^	CMGI-1 (P)	ExP, pCDF	+	[59]
	pMOL30	***pbrUb***|***Ua**TR pbrABCD* (#)	6180, 5944–5949		CMGI-30a	ExP, seq	+	[85]
	CHR1	*merRTPA′A″*	2312–2315	Hg^2+^	CMGI-1 (P)	HgRed	?	[59]
	pMOL28	*merRTPADE urf-2*	6344–6346, 6183–6186		CMGI-28a/Tn*4378*	HgRed	+	[4]
	pMOL30	*merRTPADE urf-2*	6171–6177		CMGI-30a/Tn*4380*	HgRed	+	[4]
	pMOL30	*merRTΔP*	5990–5992		CMGI-30a	HgTra	?	[10]
	CHR2	*chrBAF*	3866–3864	CrO_4_^2-^	-	ExChr	?	[5]
	pMOL28	*chrIBACEF**ONPYZ***	6204–6194		CMGI-28a	ExChr, FS, MFS	+	[12]
	CHR2	*cus**D**CBAF*	5030–5034	Ag^+^, Cu^+^,Cu^2+^	-	ExHME4	+	[8]
	pMOL30	*sil**D**CBA **cusΔF***	6133–6136, 5953		CMGI-30b	ExHME4	(+)	[10]
	CHR2	*copSRABCD*	5673–5668	Cu^2+^	-	ExCop, seq	+	[8]
	pMOL30	*cop**V**TK**MN**SRABCDI**J**GF**O**L**Q**H**EW***	6105–6119, 6382, 6120–6124		CMGI-30b	ExCop, ExP, seq	+	[75]
	CHR2	*zntA czcICΔBA ubiG czcSR**L**| **hns mmmQ*** (†)	4594–4597, 4469–4461	Cd^2+^, Zn^2+^, Co^2+^	-	ExHME1, ExP	(+)	[59]
	pMOL30	*czc**M**NICBADRSE**J ompP** czcP*	5985–5974, 5970		CMGI-30a	ExHME1, ExP	+	[10]
	CHR2	*nimBA*|*AC* (*)	5682–5677	Ni^2+^, Co^2+^	-	ExHME3b	-	[8]
	pMOL28	*cnrYXHCBAT*	6205–6211		CMGI-28a	ExHME2, CDF	+	[80], [81]
	pMOL30	*nccCB″B′A nreB **mmrQ***	6148–6143		CMGI-30b	ExHME2, MFS	(+)	[10]
**not plasmid-redundant**	CHR1	*ars**P**HC_1_BC_2_IR**M***	0327–0334	HAsO_4_^2−^, AsO_2_^−^	CMGI-7	AsRed (ExAs)	+	[94]
	CHR1	*cupric*	3523–3525	Ag^+^, Cu^+^,Cu^2+^	-	ExP	+	[75]
	CHR1	*agrCBARS*	1748–1752		-	ExHAE	?	[8]
	CHR1	*hmzRS yodB hmzBΔA*	3016–3011	divalent metals	CMGI-4	ExHME3b	-	[59]
	CHR2	*hmvCBΔA*	3836-3838		-	ExHME3a	-	[8]
	CHR2	*zniABCSR zne**P**RSCAB*	5319–5323, 5325–5330		-	ExHME3a, MSF	?	[58]
	CHR2	***hmyCB***|*hmyA* (‡)	4120–4123		-	ExHME3b	+	[8]

a chromosomal HMR resistance loci with genes that have a counterpart with a homologous function on a plasmid are called partially redundant, other loci are not-redundant.

b Underlined genes are regulators; bold genes are new in respect to an earlier report [8] (see also Table S2); geneparts created by a frameshift are indicated by ‘and“; IS- or Tn-insertions are indicated by a pipe symbol (|); truncated genes are indicated with a delta (Δ) symbol; (§), *pbrR_3_* acts on *zntA* (Rmet_4594); (#),insertion of Tn*6049* in *pbrU*; (†), *czcB* truncated by Tn*6050*-mediated rearrangement (inversion) and insertion of IS*1088* between *czcL* and *hns*; (*), insertion of IS*Rme3* in *nimA*; (‡),insertion of IS*1088* between *hmyB* and *hmyA*.

c CMGI, *Cupriavidus metallidurans* CH34 Genomic Island; (P), PAGI-2 family.

d Mechanisms of HM detoxification: ExP, efflux (P1-ATPase); pCDF, putative CDF-like; seq, periplasmic sequestration; HgRed, reduction of Hg^2+^ into Hg^0^ which then volatilizes out of the cell; HgTra, tentative transport of Hg^2+^; ExChr, transport of chromate; FS, Fe-SOD (*chrC*); MFS, major facilitator superfamily permease (i.e., *chrP*); CDF, cation diffusion facilitator; ExHME, efflux by RND-HME type 1 to 4 (see text); ExHAE, efflux by HAE-RND (unknown substrate); ExCop, extrusion of Cu^2+^; AsRed (ExAs), reduction of HAsO_4_^2−^ into AsO_2_^−^and efflux of AsO_2_^−^.

e Functionality; +, active; (+), partly active; ?, unknown; -, inactive.

We also identified hypothetical genes on CMGI-28b that encode membrane proteins rich in YD (tyrosyl-aspartyl) motifs and named them *rhsA* (Rmet_6263), *rhsB* (Rmet_6261), and *rhsC* (Rmet_6258) because such YD-repeat proteins apparantly are linked to RHS (Recombination Hot Spot) regions [87], [88]. Noteworthily, these genes are centered around the *tnpA* and *tnpB* genes (respectively Rmet_6260 and Rmet_6259) of the IS*Rme9* mobile element.

The pMOL28 backbone region covered by Rmet_6288 through Rmet_6297 provisionally was named the *pil* locus because of some resemblance in its sequence and gene order with other *pil* loci [7], [10]. However, although this region and the flanking ones display good synteny with the corresponding backbone regions on the *C. taiwanensis* pRALTA and *C. eutrophus* pHG1 megaplasmids, the individual sequence similarity of genes Rmet_6288 to Rmet_6294 to known *pil* genes is very low. Either these genes are not involved in conjugation (but rather in secretion), or pMOL28 has evolved a *sui generis* conjugative system. For now, we will retain the *pil* epitaph. We note that the Rmet_ 6291 gene, formerly annotated as *pilQ* [10], now is annotated as *pilB* because its product shows no aa sequence similarity to the PilQ usher protein encoded in the *pilMNOPQ* locus on CHR1 (Rmet_3268 to Rmet_3272); rather, it probably acts as an ATPase.

A fair number of plasmid-born genes are seemingly involved in either gene exchange or membrane processing. For instance, the entire region between the *pbr* and *czc* loci on CMGI-30a is speckled by very small hypothetical genes, transposases (*tnp*), site-specific recombinases (*int*), or remnants thereof, some of which are upregulated by heavy metals [10]. In addition, both genomic islands on pMOL30 contain a *gtrMAB* locus encoding proteins that are required for translocating oligosaccharide precursors across the cytoplasmic membrane and the subsequent glucosylation of lipopolysaccharide (LPS). Several genes of both *gtr* loci are upregulated by heavy metals [10] while the *gtr* loci are centrally placed between the *pbr* and *mer* clusters on CMGI-30a (as genes Rmet_5966/67/68) and between the *cop* and *sil* clusters on CMGI-30b (as genes Rmet_6128/29/30). A possible reason for recruiting *gtr* loci and embedding them in metal resistance clusters may be that modified (i.e., shortened) LPS chains could positively affect heavy-metal extrusion. Alternatively, GtrMAB proteins may act on other components outside of the inner membrane thereby limiting heavy-metal damage inflicted on membranes.

### Heavy metal resistance (HMR) genes in CH34

Metal detoxification in *C. metallidurans* CH34 mainly occurs via ion efflux although other systems are present (Table 5; Table S2). A variety of export systems that enlist members of three protein families assure ion efflux: The RND (for Resistance, Nodulation, cell Division) family of permeases [74], the CDF (for Cation Diffusion Factor) family of heavy metal transporters [89], [90], and the P-type ATPase family of ion pumps [91]. These export systems may act on a range of cations, including Cu^+^, Ag^+^, Cu^2+^, Co^2+^, Cd^2+^, Ni^2+^, Pb^2+^ and Zn^2+^ [8], [10], [59], [75], [80], [83], [85], [90], [92], [93]. Alternative mechanisms with specificity to other cations or oxyanions include the chromate CrO_4_^2−^ efflux system [5], the reduction of arsenate HAsO_4_^2−^ by ArsC reductases followed by the extrusion of the formed arsenite AsO_2_^−^ by the ArsB permease [94], the CopABCD/PcoABCD mediated periplasmic detoxification for Cu^2+^ [95], [96], and the reduction of Hg^2+^ into metallic mercury [4], [59], [97], [98] (Table 5; Table S2). However, the function and mode of action of many proteins encoded by putative heavy-metal resistance genes remain undetermined. Structural analysis of proteins at the molecular level offered some mechanistic information (i.e., via protein crystallization, nuclear magnetic resonance analysis, and cryo electron microscopy) for the *C. metallidurans* proteins CopK (Rmet_6108), CopH (Rmet_6122), and CzcE (Rmet_5976) expressed by pMOL30, and CnrX (Rmet_6206) expressed by pMOL28 [99]–[102]. In other cases, such data were gained from aa sequence based comparison of closely related proteins, such as for the CzcD protein (Rmet_5979) that greatly resembles the recently studied *E. coli* YiiP zinc transporter protein [103], [104].

It is worth mentioning that RND sytems for HME (Heavy Metal Efflux) were first discovered in *C. metallidurans* CH34 with the archytypes CzcCBA, conferring resistance to Co, Zn and Cd [9], [105], and CnrCBA, conferring resistance to Co and Ni [80], [106], with their genes, respectively, located on megaplasmids pMOL30 and pMOL28. Such RND-HME complexes comprise three components spanning the complete cell wall and mediating cation/proton antiporter efflux via a chemiosmotic gradient from the cytoplasm towards the periplasm and into the exterior of the cell. The main component of these complexes (i.e. CzcA, CnrA) is associated with the inner membrane and typically contains 12 transmembrane domains. An outer-membrane efflux protein (i.e. CzcC, CnrC) functions as an auxiliary element to export metals and is characterized by a channel formed by 12 beta-sheets extending further into the periplasm by alpha-helices. The third component (i.e. CzcB, CnrB) is a membrane fusion protein (MFP) bridging the two membrane-bound export proteins. This configuration effectively avoids the release of metal ions in the periplasmic space, and ensures their full disposal by the cell. Other chemiosmotic antiporter efflux systems include the NccCBA complex (Ni, Co, Cd) of *C. metallidurans* 31A (formerly known as *Alcaligenes/Achromobacter xylosoxydans*) [107], the CzrCBA complex (Cd and Zn) of *Pseudomonas aeruginosa* [108], and the CusCBA complex (Cu and Ag) of *E. coli* [109]. Remarkably, *C. metallidurans* CH34 possesses 12 such transenvelope RND-HME export devices (Table 5).

In contrast, the cation diffusion facilitator (CDF) family transporters, which act as chemiosmotic ion-proton exchangers, and the P-type ATPases, that use ATP-energy to pump metal ions out of the cytoplasm, are single-subunit systems located in the cytoplasmic membrane. Sometimes, all three types of metal-ion-efflux systems, e.g. RND-HME, P-ATPase, and CDF, occur in the same locus. For instance the *czc* cluster of pMOL30 contains besides the RND-HME module *czcCBA* also *czcD* (Rmet_5979; a CDF protein) and *czcP* (Rmet_5970; a cation efflux P1-ATPase). We and others [8], [37], [75], [76], [110], [111] suggested that this combination entails much higher resistance to heavy metals and better detoxification of cellular compartments, especially the cytoplasm (thiol pool). Together, there are at least 30 heavy-metal-efflux systems in *C. metallidurans* CH34 encoded by 155 genes in 25 loci (Table 5; Table S2). To our knowledge no other soil bacterium has acquired so many ion efflux systems specialized in removing heavy metals. We think that genomic islands likely played an important role in this procurement. This is most notable for CHR1 (viz. *cdfX pbrR cadA pbrC* in CMGI-1, *hmz* in CMGI-4, and *ars* in CMGI-7), and for the large genomic islands on the megaplasmids pMOL28 and pMOL30 encoding intertwined nested HMR loci.

In addition to this interplay of HMR determinants there is a redundancy of HMR genes across the four replicons of *C. metallidurans* CH34 (Table 5). For instance the two chromosomes of CH34 contain a number of HMR loci, i.e., *chrBAF, copSRABCD*, *pbrR-cadA/pbrA-pbrC*, and *czcICBA-RS* that have counterparts on either pMOL28 (*chr*) or pMOL30 (*cop, pbr, czc*); furthermore their products display a high sequence similarity (i.e., for the products of the *copSRABCD* locus this ranges between 50 and 87% identity over their entire length). Such duplicates may reflect multiple acquisition of the same HMR locus (possibly derived from different lineages), or may represent internal rearrangements generated by DNA mobility. However, not all of these loci (or genes within loci) are functional, as some HMR loci appear to be affected by either a frameshift mutation (e.g. the *merA* gene of CMGI-1 and the *nccB* gene of CMGI-30b) or by partial deletion (e.g. *hmzA* of CMGI-4 and *merP* of CMGI-30a ). A number of HMR loci could be extended or updated via MaGe-based annotation (Tables 5 and S2, gene names in bold face). Some of those novel genes are strongly induced by heavy metals (not shown), most notably *czcJ* (Rmet_5975) and *mmrQ* (Rmet_6143; formerly *orf-167*), and to a lesser extent *copQ* (Rmet6121). We noted previously that CczJ, MmrQ, and CopQ have a signal sequence (well defined by SignalP embedded in MaGe) and have a common domain motif, RDXXTDG, repeated 3 to 4 times [10]. A closer look learns that these domains are connected with spacers of defined length and sequence. Three other genes that are highly induced in the presence of metals, *mmmQ* (Rmet_ 4461), *mmtQ* (Rmet_4187), and *mmsQ* (Rmet_4908), encode proteins with apparent signal sequences and similar domains and spacers. Note that *mmsQ* has an almost identical paralog *mmsQ1* (Rmet_5594) thus both genes are covered by the same probe in transcriptomic analysis. The *mmmQ* gene is linked to the *czc* locus on CHR2 (Table 5) displaying excellent synteny with metal efflux loci in the three other *Cupriavidus* genomes (in spite of an IS*1088* insertion between the *mmmQ* and the *czc* genes). In contrast, the other genes *mmtQ, mmsQ* and *mmsQ1* appear to be unlinked to any HMR locus. The metal induction of these seven genes (*czcJ*, *mmrQ*, *copQ*, *mmmQ*, *mmtQ*, and *mmsQ/Q1*) follows a varied and overlapping response, with very high induction by some metals and no induction by other metals. For example, *czcJ* and *mmrQ* are strongly induced by Cd^2+^, Cu^2+^, Ni^2+^, and Zn^2+^, moderately induced by Pb^2+^, and not induced by Hg^2+^, while the *mmtQ* gene is highly induced by Cd^2+^, Hg^2+^, and Pb^2+^, only moderately by Zn^2+^ and not by Cu^2+^ or Ni^2+^ (data not shown). Much to our surprise, according to BLAST analyses against GenBank, these genes (with *copQ* as prototype) and their orthologs are restricted to the genera *Cupriavidus* and *Ralstonia*. We are currently extending this analysis using pattern matching software to search for CopQ-like protein sequence motifs across multiple genomes. We are also performing additional transcriptomic studies on these genes induced by a wider range of metals and other types of stress, and are investigating their gene upstream regions for regulatory DNA sequences.

Through genome annotation, we also found additional candidate HMR genes that warrant further investigation. They are genes *dmeF* (Rmet_0198 on CHR1) and *fieF* (Rmet_3406 on CHR2) encoding CDF-type transporters, and the *atmA* gene (Rmet_0391 on CHR1) coding for an ABC-type transporter. According to MaGe data, these genes have orthologues in the three other *Cupriavidus* species and are well-conserved in other bacteria. Interestingly, disrupting *dmeF* in CH34 causes the Cnr and Czc systems of pMOL28 and pMOL30, respectively, to become ineffective against Co^2+^, indicating that *dmeF* is essential in cobalt homoeostasis [112]. Correspondingly, *fieF* mutants of strain CH34 showed decreased resistance to Ni^2+^, Co^2+^, Cd^2+^ and Zn^2+^ [112] and *atmA* mutants of strain CH34 exhibit lower resistance to Ni^2+^ and Co^2+^ [113].

Clearly, *C. metallidurans* CH34 has a history of colonizing biotopes with moderate to high-levels of bioavailable metals and, via the uptake and reshuffling of HMR loci, adapted very well to such extreme conditions. This adaptation required the dedicated management of incoming HMR systems to avoid functional redundancy (i.e., under stress when resources are scarce), the optimization of plasmid-born genes to counter elevated levels of metal toxicity, and the economical interplay between plasmid- and chromosomal-genes, supported by a large diversity of sigma subunits of RNA polymerase [114]. We expect that whole-genome transcriptomic studies using a wide range of metal cations and -oxyanions will reveal additional HMR genes and highlight new interplays between chromosomal and plasmid borne genes. Such analyses also will refine our knowledge about the intricate regulation patterns apparantly governing the response to high concentrations of heavy metals.

### Small-molecule transport and protein secretion systems

The four *Cupriavidus* genomes were submitted to the TransportDB [115], [116] website (http://www.membranetransport.org/) to identify efflux systems and transporter families by the TransAAP (Transporter Automatic Annotation Pipeline) tool. The TransportDB relational database holds the complete membrane transport complements of 288 bacteria classified into protein families according to the Transport Classification (TC) system [117], [118]. The recently updated TC system organizes nearly 5,000 representative transporters in more than 500 families [118] and is freely accessible at http://www.tcdb.org/. From the mode of transport and the mechanisms of energy coupling, TCDB discerns four major classes of transporters: Channels; secondary carriers; primary transporters; and group translocators. Each class is divided in families and subfamilies according to their function, phylogeny, and substrate specificity. In our analysis with TransAAP we consider only five trans-membrane families: 1.A: alpha-Type channels; 2.A: Porters (uniporters, symporters, antiporters); 3.A: P-P-bond-hydrolysis-driven transporters; 4.A: Phosphotransfer-driven group translocators; and 9.A: Recognized transporters of unknown biochemical mechanism. A more extensive analysis using all 9 TC classes was reported albeit on the unfinished genome sequence [119]. The numbers of transport proteins predicted by our TransAAP analysis were about the same in all four *Cupriavidus* species (Table S3), amounting to 12 to 14% of their total number of proteins. This appears to be typical for β-proteobacteria [120]. In addition the distribution of transporter classes by species is very similar (Table S3). The largest group of transporters (ca. 400 per species) is presented by the porters (TC2.A), closely followed by the primary transporters (TC3.A). The largest families herein are the Major Facilitator Superfamily (MSF) (TC2.A.1) and the ATP-binding Casette (ABC) Superfamily (TC3.A.1) that are involved in the uptake of various nutrients, such as amino acids, sugars, nucleosides and various organic and inorganic anions. They also remove antibiotics, metabolites, and toxic byproducts. A third large group is the Tricarboxylate Transporter (TTT) Family of solute receptors (TC2.A.80) that carry a typical carboxylate-binding motif (the “Bug domain”), and probably is involved in diverse metabolic and transport activities (discussed further under ‘Signal Transduction’). These three large families constitute up to 70% of all predicted transporter proteins in the four *Cupriavidus* strains. Of immediate interest to our research on *C. metallidurans* are the systems involved in transporting transition metals: MIT (1.A.35), CDF (2.A.4), ZIP (2.A.5), RND (2.A.6), CHR (2.A.51), NiCoT (2.A.52), P-type ATPase (3.A.3), MerT (9.A.2), and TerC (9.A.30) (with TC class in parentheses). These systems, their genes, and their regulation were discussed in detail elsewhere [8], [10], [75], [76]. Evidently, the cellular household of *C. metallidurans* CH34 is tuned towards adaptating to a wide range of heavy-metal species (see our brief update on HMR genes earlier in this discussion) or to surviving in biotopes that are either occasionally or constitutively rich in heavy metals. Unsurprisingly therefore, *C. metallidurans* and its related strains most often are found in heavily polluted anthropogenic- or industrial-environments [6], [8].

Only a few transporter systems are unique to *C. metallidurans* CH34 compared to the other three *Cupriavidus* species (Table S3): they are the 2-Hydroxycarboxylate Transporter (2-HCT) Family (TC2.A.24), and the Iron/Lead Transporter (ILT) Family (TC9.A.10). The former is represented in *C. metallidurans* by gene Rmet_4998 (situated on CHR2 and listed in MaGe as encoding a citrate carrier) while the latter is represented by two members Rmet_5945 and Rmet_6106 (both located on plasmid pMOL30 as part of the *czc* and *pbr* operons, respectively).

Nine transport systems not present in *C. metallidurans* were found in at least one of the other *Cupriavidus* species (indicated in gray in Table S3) but apparently this observation lacks physiological relevance. However, no member of the Monovalent Cation (K+ or Na+):Proton Antiporter-3 (CPA3) Family (TC2.A.63) was detected in *C. metallidurans*, while the other three *Cupriavidus* species have seven to nine predicted transport proteins belonging to this family. A closer examination reveals that these predicted proteins are subunits of a large multicomponent antiporter with their encoding genes organized as a single operon (e.g., denoted as the *mnhABCDEFG* operons in *C. eutrophus* H16 and *C. taiwanensis*). Such antiporters were linked in *Bacillus* species with cellular responses to short-term changes in salinity or pH [121]. Interestingly, MaGe synteny maps show that *D. acidovorans* and *B. petrii* have exactly the same set of genes in one single locus and so does *Sinorhizobium meliloti* and *Bacillus halodurans* but not *Ralstonia pickettii* nor any of the three *R. solanacearum* species listed in the MaGe databases.

*C. metallidurans* CH34 possesses fully intact Sec-dependent and Sec-independent (twin-arginine translocation “TAT”) protein export pathways for secreting proteins across the inner/periplasmic membrane. The Sec-dependent pathway consists of a protein translocation channel made by the contact of two heterotrimeric complexes SecYEG (encoded by Rmet_3297, Rmet_3340, and Rmet_0926) and SecDFYajC (encoded by Rmet_2945-47). The SecA and SecB proteins (encoded by Rmet_3118 and Rmet_0254, respectively) cooperatively bind with the signal sequence of secretory proteins that, after translocation, is cleaved off by signal peptidases SPaseI (Rmet_2420) or SPaseII (of which there are three candidate enzymes in *C. metallidurans* i.e. encoded by Rmet_2304, Rmet_2886, and Rmet_5948). Here, an important accessory component is the membrane insertase YidC (Rmet_3613) that may act independently from the Sec translocase and is involved in folding the inner membrane proteins and the biogenesis of respiratory-chain complexes (listed under TC class 2.A.9 in Table S3). Genome analysis of *C. metallidurans* also identified Rmet_3094 encoding a signal recognition particle (SRP54) that acts with FtsY (Rmet_0281) as an alternative to the SecA-SecB pair for targeting protein precursors. The TAT secretion pathway is specialized to translocate proteins that already have undergone folding in the cytoplasm [122]. It consists of the three subunits TatA, TatB and TatC (encoded by Rmet_3235-27; listed under TC class 2.A.64 in Table S3) that form distinct multimeric TatA or TatABC complexes acting together at the point of translocation.

Extracellular secretion of folded proteins across the outer membrane depends on the Type II Secretion System (T2SS), also often referred to as the “general secretion pathway (gsp)”. In proteobacteria this apparatus comprises 12 to 16 different gene products forming a multiprotein complex spanning the periplasmic compartment; it shares many features with the type IV pilus biogenesis system, including the ability to assemble a pilus-like structure. There are two clusters of *gsp* genes in *C. metallidurans* CH34. The largest cluster of 11 genes *gspGHIJKLMNDEF* (Rmet_3384-94) is situated on CHR1 and is well conserved in the four *Cupriavidus* species, while CHR2 (Rmet_4704 through Rmet_4713) supports another smaller cluster consisting of *gspD, gspEFG*, two additional copies of *gspG*, and four conserved hypothetical genes (three of which predictably code for transmembrane proteins). Lateral gene transfer may have allowed the acquisition of this genomic region, which does not display any synteny with the genomes of the other three *Cupriavidus* species. Interestingly, this cluster of genes is flanked by two IS elements in *R. solanacearum* GMI1000. We detected around 30 genes required for type-IV pilus biogenesis and assembly in *C. metallidurans*, some of which occurred in multiple copies across the genome (see also section on motility).

There is no direct evidence for a functional pathogenicity-related Type III Secretion System (T3SS) in *C. metallidurans*. However, all the *fli* and *flh* genes encoding the structural proteins for a basal flagella body, i.e., those that display homology to T3SS genes through common ancestry, are present (Rmet_3689-99, Rmet_5258, Rmet_5261, Rmet_5263-64, Rmet_5299, and Rmet_5301-03) together with all additional *fli*, *flh*, *mot*, and *flg* genes required for assembling full flagella (see section on motility).

The Type IV Secretion System (T4SS) is a multifunctional family of translocation pathways that mediate the transfer of macromolecules (proteins, DNA or DNA-protein complexes) across the bacterial cell envelope either to the outside medium or to other cells (prokaryotic or eukaryotic). These systems, widespread in nature, are evolutionary related to bacterial conjugation systems and largely contribute to the rapid dissemination among bacterial populations of a range of catabolic- and metabolic-genes and of antibiotic resistance genes and other virulence traits. Moreover, because often they are sited on plasmids or other mobile genetic elements, many T4SS are laterally transferred between species. We discerned two T4SS gene clusters in the *C. metallidurans* genome, each with nine genes homologous to the *vir* genes of the Ti-plasmid of *Agrobacterium tumefaciens* (Rmet_1336 through _1350 and Rmet_1546 through _1559). These clusters are located on CHR1 as part of Tn*4371*-like mobile genetic elements (MGEs) and may be involved in GI maintenance or conjugational transfer [66]. We denoted these MGEs as genomic islands CMGI-2 and CMGI-3; they carry large sets of genes required for chemolithotrophic growth and degrading aromatic compounds (addressed elsewhere in this discussion). A third homologous cluster of ten genes, mostly denoted as *trb* or *tra* genes (Rmet_6299 through _6309), is present on plasmid pMOL28 preceeding the *uvrD-parAB* genes. These *trb/tra* genes probably contribute to the maintenance and conjugal transfer of the pMOL28 plasmid and are part of the plasmid backbone common to a range of megaplasmids, including pHG1 of *C. eutrophus* strain H16 and pRALTA of *C. taiwanensis* [7], [10].

### Sigma factors and transcriptional regulators

An important strategy of bacteria is to use alternative sigma factors to cope with environmental changes; *C. metallidurans* CH34, with eighteen sigma factors, appears to be particularly poised to combat stress. We generally adopted the earlier proposed description [76], [114], [123] for annotating these eighteen sigma factors, except for the housekeeping sigma-70 factor. In the initial annotation [123], gene Rmet_4661 located on CHR2 was named as *rpoD* while a second RpoD-like protein (encoded by Rmet_2606 situated on CHR1) was named as RpoG [114]. However, this latter annotation was cumbersome. In updating the genome sequence, it became evident that this ambiguity reflected a sequencing error by which only the carboxyterminal part of RpoG displays conserved sigma factor domains (regions 1.1 and 1.2), while the erroneous aminoterminal part does not reveal any features typical of sigma factors. In this updated version, the Rmet_2606 protein now displays all proper characteristics of a sigma-70 housekeeping factor, and has a high homology with the *E. coli* RpoD (56% aa identity, minLrap 0.99). Consequently we propose to rename RpoG as RpoD1 and its gene Rmet_2606 as *rpoD1*. In addition, since the Rmet_4661 protein displays a good similarity with the *E. coli* RpoD (34% aa identity, minLrap 0.99) and clearly contains all necessary domains to be a functional housekeeping sigma factor, we propose renaming RpoD as RpoD2 with its gene Rmet_4661 as *rpoD2* (i.e. replacing *rpoD*).

The presence of multiple sigma-70 housekeeping factors is not unique to *C. metallidurans*: *C. eutrophus* H16 and *C. taiwanensis* both contain two sigma-70 factors (albeit both located on the main replicon) while *C. pinatubonensis* JMP134 contains three copies of this sigma factor (one on each chromosome and one on the megaplasmid) (Table S4). In our study, only *B. xenovorans* contained four sigma-70 genes, all on its second chromosome. Only one copy was detected in the other genomes we analyzed (of *R. solanacearum*, *H. arsenicoxydans*, *Janthinobacterium, Polynucleobacter*, *B. petrii*, *D. acidovorans*, and *E. coli*).

Pairwise Smith-Waterman alignments of RpoD protein sequences for all analysed genomes (represented in Figure S5 as a heatmap) showed that *C. metallidurans* RpoD1 (Rmet_2606) forms a tight group with orthologous RpoD proteins from the three other *Cupriavidus* species (Reut_A0888, RALTA_A2226 and H16_A2725) whose genes all sit on CHR1. In contrast, in this same analysis, *C. metallidurans* RpoD2 (Rmet_4661) did not group with any of the other RpoD orthologs in the *Cupriavidus* family (Figure S5); indeed, it had more significant BlastP hits with the RpoD proteins of *Bordetella* and *Burkholderia* species. Therefore we believe that the *C. metallidurans* RpoD2 protein originates from another lineage. The co-existence of multiple sigma-70 factors may be useful for regulatory intricacy. Assuming that these factors would recognize the same set of promoters, slight nuances in factor binding and/or DNA interaction support finely tuned expression patterns such as when cells enter a different growth mode, or at the onset of a particular stress condition. It is unclear how this complexity in transcriptional regulation is attained. It would be interesting to undertake mutational analysis on either one of the *C. metallidurans* rpoD genes (Rmet_2606, Rmet_4661) to check their functionality, to follow cell viability of mutants, and subsequently to measure whole-genome gene expression under a variety of conditions.

Grosse *et al.* [114] described at least ten sigma factors in *C. metallidurans* CH34 that belong to the extracytoplasmic function (ECF) family, most of which were implicated in HMR. These authors also showed that one particular sigma factor, RpoI (Rmet_1120) is essential for producing siderophores in CH34 (see following section) and that this sigma factor and two other related ones, RpoJ (Rmet_4001) and RpoK (Rmet_4499), were induced by iron depletion. Consequently, the researchers classified these three factors as belonging to the ECF:FecI group (type 1) to indicate a similar function in iron assimilation as FecI in *E. coli*. Interestingly, the other three *Cupriavidus* species (Cpin, Ceut, and Ctai) do not possess RpoJ or RpoK factors while only *C. taiwanensis* has an RpoI ortholog (RALTA_B1239) albeit with weak similarity (37% aa identity, minLrap 0.98) (Table S4).

The complex transcriptional behavior of *C. metallidurans* not only is manifested by the many alternative sigma factors, but also its high count of transcriptional regulators. Using MaGe-based data for CH34 at least 543 transcription factors were discerned, corresponding to 9% of all genes in the genome, while the average percentage transcription factors is only 2.4% for the 673 bacteria in the DBD database (http://www.transcriptionfactor.org/) [124], [125]. This large difference partially may be explained by the facts that the relative number of transcriptional regulators increases with genome size [126], and generally depends on the organism's life-style [127], [128]. Nonetheless, our analysis shows that only *Bordetella pertussis* strain Tohama I, six *Burkholderia* species, and *Streptomyces coelicor* display a higher density of transcriptional regulators. Based on Pfam predictions [129], roughly a quarter of all regulators in *C. metallidurans* belong to the LysR family (n = 126) (Table S5). Members of this family typically repress transcription from their own promotor, but activate transcription from a divergent promotor. The products of these activated genes perform a wide range of functions [130] while their activation is mediated by co-inducers, such as extracellular signaling molecules, metabolites, or ions. Other important one-component systems in *C. metallidurans* were GntR (n = 43), TetR (n = 35), AraC (n = 25), MarR (n = 16), and IclR (n = 15) (Table S5), all of which contribute to the transcriptional control of stress responses, carbon metabolism, catabolic pathways, multidrug efflux pumps, biosynthesis of antibiotics, response to osmotic stress and toxic chemicals, differentiation processes, and virulence and pathogenicity.

In terms of one-component metalloregulatory proteins in CH34, three families MerR (n = 10), ArsR (n = 6), and Fur (n = 2) deserve our attention. Although these regulators are evolutionary related in their DNA binding properties, their mode of action is different [131]. While MerR-type regulators nearly exclusively function as transcriptional activators, ArsR-type regulators generally act as repressors that upon metal binding dissociate from the DNA giving way to the RNA polymerase (coined as transcriptional derepression). Fur proteins although being typical repressors can also activate gene expression, either directly, or indirectly via the Fur-dependent repression of a small regulatory antisense RNA. Importantly, there is a wide range in metal selectivity within these three families. Metal sensing in MerR regulators is governed by the effector binding domain in their C-terminal region. Structural diversity in this domain allows MerR family proteins to sense not only various metal ions, e.g., Zn^2+^ by ZntR, Au^+^ by GolS, Cd^2+^ by CadR, Pb^2+^ by PbrR, or Hg^2+^ by MerR, but also oxidative stress, e.g., by SoxR via an [2Fe-2S]^2+^ cluster (reviewed in [131]. Preferential sensing by MerR regulators of one metal ion over another, or monovalent ions over divalent ions, is defined by the positioning of Cys and His but also other conserved residues, mainly in the DNA binding loop. The stringency of this preference probably varies among MerR regulators as some respond to more then one metal. In strain CH34 there are four MerR proteins and three PbrR proteins encoded by the *mer* and *pbr* loci (regulators are underlined in Table 5), and one CupR (Rmet_3523) which responds to Cu^+^ and Au^+^ [35]. The remaining MerR-type regulators in CH34, including SoxR (Rmet_4538), are not physically linked to any structural HMR gene.

The metal sensing in ArsR regulators in contrast is not defined by a single metal binding pocket but is distributed over distinct locations of the repressor involving a number of alpha helices containing key Cys or His residues [131]. Members of this family sense, next to As(III) and Sb(III), a wide variety of divalent metal ions including Zn^2+^, Ni^2+^, Co^2+^, Pb^2+^, and Cd^2+^. Some ArsR family members display sensitivity to multiple metal ions and are able to respond to either monovalent or multivalent ions, probably as a result of evolutionary adaptations in their protein structure particularly in the α3N and α5 helical regions. The CH34 genome enlists only one *bona fide* ArsR regulator (Rmet_0333 of the *ars* locus on CHR1; Table 5). This repressor has been shown to dissociate from its operator DNA in a metal and valence qualifying manner [94], with three groups of heavy metals displaying decreasing degrees of ArsR dissociation: As(III)/Bi(III)/Co^2+^/Cu^2+^/Ni^2+^; Cd^2+^; and Pb^2+^/Zn^2+^. No ArsR dissociation was observed in the presence of As(V). The other five ArsR family proteins of CH34 appear to be unlinked to any HMR gene cluster except Rmet_5676 which is located adjacent to the defective *nim* locus on CHR2 (Table 5). The two Fur proteins in CH34 are discussed in the next section on siderophores. Besides the MerR, ArsR, and Fur families, no other structural families of one-component metal responsive regulators are currently recognized in the CH34 genome. Although also LysR, TetR, and MarR families reportedly contain members that are directly involved in metal homeostasis or metal-regulated responses [131], only very few well-documented cases are known sofar. Nonetheless, these three families are represented in CH34 by a total of a few hundred proteins so it may be worthwhile to look also within this pool for further candidate metal response regulators.

Among two-component systems the OmpR system constituted the largest group (n = 33) while the LuxR family (best known for its quorum sensing regulators) also was well represented (n = 41) (Table S5). According to the MiST2 database [132], there are 56 two-component pairs of adjacent genes in the CH34 genome. Several of those have been implicated in HMR, notably two pairs of *copRS* and *czcRS*, and one pair each of *agrRS*, *hmzRS*, *zniRS*, and *zneRS* (underlined in Table 5). From MaGe data and the DBD and MiST2 databases we discovered that the second replicon of *C. metallidurans* (CHR2) carries proportionally almost twice the number of regulatory genes as compared to the main replicon CHR1. This enrichment in regulatory genes agrees well with the earlier notion that CHR2 carries many functions relevant to the survival and responsive behavior of *C. metallidurans* CH34 (Figure 2).

Clearly, the wide diversity of sigma factors and transcriptional regulators in *C. metallidurans* CH34 points to intricate regulatory mechanisms for activating genes in response to environmental stimuli. For instance, microarray analyses of genome-wide gene expression studies on strain CH34 pointed to layered multiple responses when cells are challenged with heavy metals [10]. Similar complex expression patterns were shown for CH34 cells grown under other harsh conditions, including simulated microgravity, growth on basalt, and spaceflight (data not shown). To unravel these regulatory mechanisms and better understand the molecular basis of *C. metallidurans'* ability to survive prolonged periods of stress, we now are focussing on reconstructing the regulatory networks by systematically identifying regulatory motifs in the upstream regions of stress-induced and co-regulated genes.

### Siderophores

At neutral pH and oxic conditions, bacteria cannot readily utilize low-solibility Fe(III) minerals. Accordingly, they synthesise and secrete soluble iron-complexing compounds, called siderophores. *C. metallidurans* CH34 grown under iron-limiting conditions produces a unique phenolate-type siderophore containing neither hydroxamate nor catecholate groups [133]. This siderophore, originally referred to as “alcaligen E”, eventually was classified as a polycarboxylate staphyloferrin B [134] because it resembled the siderophores typically produced by staphylococci. It since was recognized that other organisms produce staphyloferrin B, such as *R. solanacearum* [135]. Notably, *C. metallidurans* staphyloferrin B can interact with Cd^2+^ thereby decreasing the metal's bioavailability and hence lowering its cellular toxicity in the bacterium. Also, siderophore-producing *C. metallidurans* tested in bioaugmentation-assisted phytoextraction increased, by a factor of 5, the accumulation of Cr(VI) in plant shoots [136]. Future studies on siderophore production in *C. metallidurans* CH34 might thus open up exciting lines of research in heavy metal tolerance and bioremediation.

Genes for staphyloferrin B biosynthesis (Rmet_1109-1118) and two genes encoding ferric/ferrichrome siderophore TonB-dependent receptors BfrC and FcuA (Rmet_1104 and Rmet_1108, respectively) are located on CHR1 within a 52 kb region that lacks synteny with Cpin, Ceut, and Cai. Although a tRNA-Ser gene preceeds this region, we could not identify any tyrosine-based site-specific recombinases, flanking IS elements, or direct repeats; hence, we did not classify it as a genomic island (see our discussion on Mobile Genetic Elements). It includes genes encoding a siderophore synthase decarboxylase (*ssd*, Rmet_1110), an aldolase (Rmet_1111), staphyloferrin B biosynthesis proteins (Rmet_1112, Rmet_1113, and Rmet_1115), a permease of the Major Facilitator Superfamily (MSF) (Rmet_1114), an ornithine cyclodeaminase (*ocd*, Rmet_1116), needed to biosynthesize alcaligin [137], a cysteine synthase (*cysK*, Rmet_1117), and the staphyloferrin B receptor (*aleB*, Rmet_1118). This core set of genes (Rmet_1110 to Rmet_1117) displays high synteny with the staphyloferrin B biosynthesis regions of *Ralstonia solanacearum* GMI1000 and *Staphylococcus aureus* MW2 [135] and of all other *S. aureus* strains present in the MaGe database. In addition, *R. solanacearum* GMI1000 and *C. metallidurans* CH34 share the *aleB* gene (Rmet_1118) and a gene for the sigma-19 transcription factor FecI (RpoI; Rmet_1120). The insertion of a mini-transposon Tn*5* into *aleB* decreased iron uptake three to four times in iron-starved cells compared to the wild type strain CH34 [133]. The *C. metallidurans* CH34 genome lacks the *entA*, *entB*, *entC*, *entD*, *entF,* and *entS* genes required for enterobactin biosynthesis but seems to contain an *entE* gene (Rmet_3777) on CHR2.

The regulation of staphyloferrin B biosynthesis is controlled by two systems: A *fecRI* regulon located immediately upstream of *aleB*, and the *fur* gene distant from *aleB*. The regulator FecR (Rmet_1119) is a sensor for the presence of iron dicitrate in the periplasm and regulates its transport by inactivating FecI (Rmet_1120). The Fur protein (for ferric uptake regulation) tightly represses staphyloferrin B biosynthesis under iron-replete conditions [138]. In both *C. metallidurans* and *R. solanacearum*, a gene (Rmet_2977) encoding the global virulence regulator PhcA is located next to the *fur* gene (Rmet_2976). In *R. solanacearum*, PhcA negatively regulates staphyloferrin B biosynthesis [135]. Furthermore, the *phcA* gene of *C. metallidurans* CH34 can complement a *phcA^−^* mutant of *R. solanacearum* [139]. Interestingly, two Fur-like regulators, Rmet_0128 and Rmet_5746, respectively, were found on CHR1 and CHR2.

Besides the *aleB* locus, the genome of *C. metallidurans* CH34 contains additional genes that are probably involved in binding siderophores. These are Rmet_0837 and Rmet_4617 encoding hydroxamate-type ferrisiderophore receptors, Rmet_4496 and Rmet_4497 encoding ferric siderophore receptors, Rmet_5806 and Rmet_5807 encoding FecA-type ferric citrate outer membrane receptors, a small locus Rmet_2277/8/9 encoding a complete TonB-ExbB-ExbD type energy-transducing complex. Supplementary genes encode a TonB-like protein (Rmet_3055) and ExbB/ExbD homologues (Rmet_0536/7). The presence of multiple siderophore binding and translocation systems is consistent with the observation that *C. metallidurans* CH34 employs various ferric ion chelators for iron uptake, including desferriferrioxamine B, D2 and E, desferriferricrocin, desferriferrichrysin, desferriferrichrome A, and ornibactin [133]. In addition, its genome encodes proteins involved in iron storage (*bfr*, Rmet_0195, Rmet_0248) and soluble ferrous Fe(II) transport (*feoB*, Rmet_5890 and Rmet_5890). Table S6 offers an overview of CH34 genes implemented in iron uptake and -metabolism.

### Signal transduction

Like many other environmental bacteria, *C. metallidurans* CH34 deploys early warning systems and response mechanisms to cope with many different processes such as growth and development, metabolic regulation, and sensing, and to react swiftly to various stresses. In bacteria, the linkage of input signals to output responses entails a cascade of protein modifications, basically of two modes: One-component and two-component systems (denominated as 1CST and 2CST, respectively). In 1CST systems, the input- and output-domains are fused in a single protein, whereas in 2CST systems, the sensory- and receiver-domains lie on two different proteins (reviewed by Galperin and Gomelsky [140]). The latter has the advantage that the sensory component can be membrane-associated to receive *extra*cellular stimuli while the receiver domain can be part of the actual regulator, often acting at the level of transcription. Until recently, 2CST systems were thought to be the primary mode of signal transduction in prokaryotes, but it became evident that 1CST systems carry out much of the signal transduction [128]. The input and output domains of these systems display a remarkable diversity, are widely distributed across species, and are mainly involved in DNA binding and the *intra*cellular detection of environmental stimuli [128]. To survey signal transduction proteins in *C. metallidurans* and related organisms we used the Microbial Transduction System (MiST) database [141] (http://genomics.ornl.gov/mist/) that holds the predicted signal transduction proteins for nearly 750 bacterial and archaeal organisms, using Pfam and SMART reference domains (added note: an extended database MiST2 was very recently established at http://mistdb.com/ [132]). These data demonstrated that all four *Cupriavidus* species contained approximately the same proportional number of proteins putatively involved in signal transduction (approx. 9–10% of their proteome) (Table S7). The same proportion was evident in *D. acidovorans*, *Janthinobacterium,* and *B. xenovorans*, while *B. petrii*, *R. solanacearum,* and *H. arsenicoxydans* displayed slightly lower values (8.3-, 8.2- and 7.0%, respectively). In contrast, and in line with its deterministic lifestyle, the intracellular symbiont *P. necessarius* STIR1, whose single replicon displays good synteny with *C. metallidurans* CHR1 (see section on Phylogeny), deploys only 1.6% of its protein complement for signal transduction. For all the organisms we compared, there were two- to three-fold more predicted proteins involved in 1CST systems than in 2CST systems (Table S7) (the only exception being *B. petrii* that has a slightly higher preference for 1CST systems). Also, this comparison demonstrated that the second replicon of all organisms with a multipartite genome (i.e. the four *Cupriavidus* species, *R. solanacearum*, and *B. xenovorans*) displayed an enrichment on their second replicon for proteins involved in signal transduction (data not shown), once more underpinning the importance of this replicon for adaptation and survival.

According to MiST data, protein domains commonly used for signal transduction in *C. metallidurans* CH34 were Bug (123), LysR_substrate (122), Response_reg (98), HATPase_c (72), HisKA (55), HAMP (45), TetR_N (36), GGDEF (30), PAS (30), FCD (24), EAL (23), MCPsignal (16), Sigma54_activat (16), and GAF (10) (domain names are in Pfam notation with numbers in parantheses). Most of these domains are integrated as signal receivers into transcriptional regulators (e.g. LysR_substrate, TetR_N, FCD) whether in 2CST or 1CST format, while others have more specific functions suggesting a role in chemotaxis (e.g. Response_reg, MCPsignal), His-Asp phosphorelay (HisKA, HAMP, HATPase_c), cyclic di-GMP turnover (GGDEF, EAL, GAF), or solute binding (e.g. Bug). Undoubtedly, many output domains constitute DNA binding motifs for the transcriptional regulation of genes. These domains typically have helix-turn-helix (e.g. HTH_1, IclR) or winged helix (e.g. Trans_reg_C, MarR) structures (Table S7, not all data shown).

**Figure 5:**
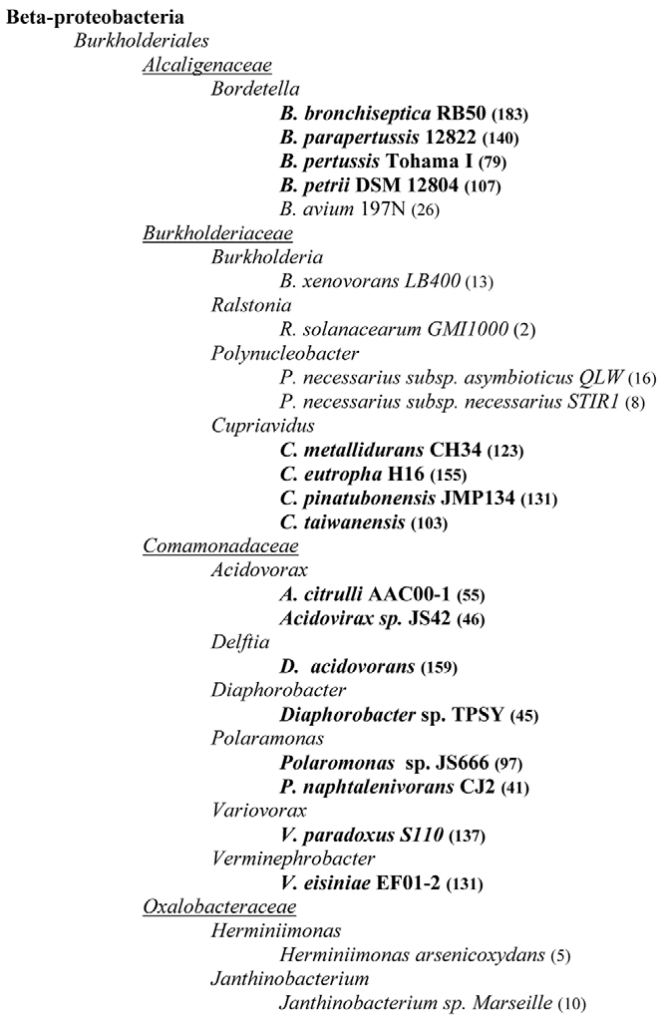
Distribution of the Bug domain. Overrepresentation of ‘Bug’ domains in the family *Burkholderiales* according to MIST data (http://mistdb.com/), with numbers in parantheses. Organisms encoding more then 40 Bug domain-containing proteins are in bold. Only relevant taxa and species are shown (see text). Detailed data are in Table S8.

In general, the distribution of signal transduction domains is the same across the four *Cupriavidus* species (Table S7) and is not very different from their distributions in the other freeliving species. However, we noted an important deviation for the Bug domain that occurs at high frequency (>100) for all four *Cupriavidus* species but is far less prevalent in either *R. solanacearum* or *B. xenovorans* (respectively, 2 and 13 Bug domains). The Bug domain, named after the *Bordetella pertussis bugT* gene identified by Antoine *et al.* [142], was reported as the first member of a large family of periplasmic solute-binding receptors present in in great numbers (up to 181 per genome) in *Bordetella* species, with much lower numbers (1 to 11) in some other bacterial genomes [47]. The only exception in their study was *C. metallidurans* for which 102 Bug homologs were predicted. Typically, Bug proteins are highly conserved in size (median size 325 residues), contain an identifiable signal-sequence, and share well-conserved carboxylate-binding motifs that form a characteristic Venus-fly-trap fold [143], [144]. This latter characteristic and the fact that the family member TctC, a periplasmic citrate-binding protein of *Salmonella typhimurium*, is part of a novel transport-system TctABC, led researchers to classify many members of this family to the tripartite tricarboxylate transporter (TTT) family (listed as TC 2.A.80 in the Transport Classification Database; http://www.tcdb.org). Nonetheless, although Bug proteins clearly form a distinct family of solute receptors, their sequences have diverged widely and their genes often are not linked to transport-related operons. Antoine and co-workers [47], [145] posited that these *bug* genes may be used by other transport systems, or involved in other metabolic activities or other pathways activated under stress. In this context it is interesting to note that many of the *bug* genes in CH34 seem linked or are in the close vicinity of transporter and regulatory genes. We also find it intriguing that *C. metallidurans*, and the other *Cupriavidus* species, harbour so many *bug* genes: Out of 200 genomes analysed by Antoine *et al.* [47], only *Bordetella* species and *C. metallidurans* were listed with a prodigious number of *bug* genes. We might argue that *Cupriavidus* species are free-living organims wherein the use of solute receptors for specialised strategies of adaptation and survival evolved, but then, we question why so few other free-living organisms failed to develop this approach? We surveyed for the Bug domain in the 1,216 complete and draft, bacterial and archaeal genomes currently in the MiST2 database [132] and found 2,377 proteins with Bug domains. Outside the genera *Bordetella* and *Cupriavidus* only four genomes had a comparable count: *Delftia acidovorans* SPH-1 (159), *Variovorax paradoxus S110* (137), *Verminophrobacter eiseniae* EF01-2 (131), and *Polaromonas* sp. JS666 (97). The only other genomes with a large count (>40) were *Diaphorobacter* sp. TPSY (45), *Acidovorax avenae* ssp. *citrulli* (55), *Acidovorax* JS42 (46), and *Polaramonas naphtalenivorans* CJ2 (41). To our surprise, all organisms that showed an overrepresentation of *bug* genes belonged to the same beta-proteobacterial order of *Burkholderiales* (Figure 5; Table S8). We propose to call this group of prokaryotes the “Bug Receptor Bacteria” or BuRBs. Such overrepresentation may be a recent innovation in bacteria, or may be more useful to those with a particular lifestyle and habitat. Whether the propensity to utilise large numbers of Bug-domain containing receptors arose within the genus *Bordetella* and was radiated out to closely related genera, or whether it has originated elsewhere within the order of *Burkholderiales* is an open question. It is useful here to mention that *B. petrii*, which carries 107 *bug* genes, is not host-restricted like the other *Bordetella* species. It is an environmental species typically obtained from river sediment, grass root, and polluted soil [146], [147] and has the capacity to degrade aromatic compounds and detoxify heavy metals [148]. However, *B. petrii* strains subsequently were isolated from patients with mandibular osteomyelitis and chronic suppurative mastoiditis [149], [150]; thus, this species appears to be an opportunistic pathogen after all. It also contains many mobile genetic elements [148], some strikingly resembling those found in CH34 [59], that may have played an intermediate role in the evolution of BuRBs and the spread of the Bug domain. We speculate that BuRBs evolved many Bug-domain proteins as periplasmic receptors to expand their cellular capacity to bind and transport, via the Venus fly trap fold, a wide range of environmental carboxylated compounds. They might include aminoacids (glutamate, aspartate), other forms of C-skeletons useful for growth (oxalate, malate, succinate, lactate, citrate), humic-acid derivatives from soil, metal-complex carboxylated acids in polluted environments, or plant-root-exuded carboxylates naturally accumulating in the rhizosphere.

**Figure 6:**
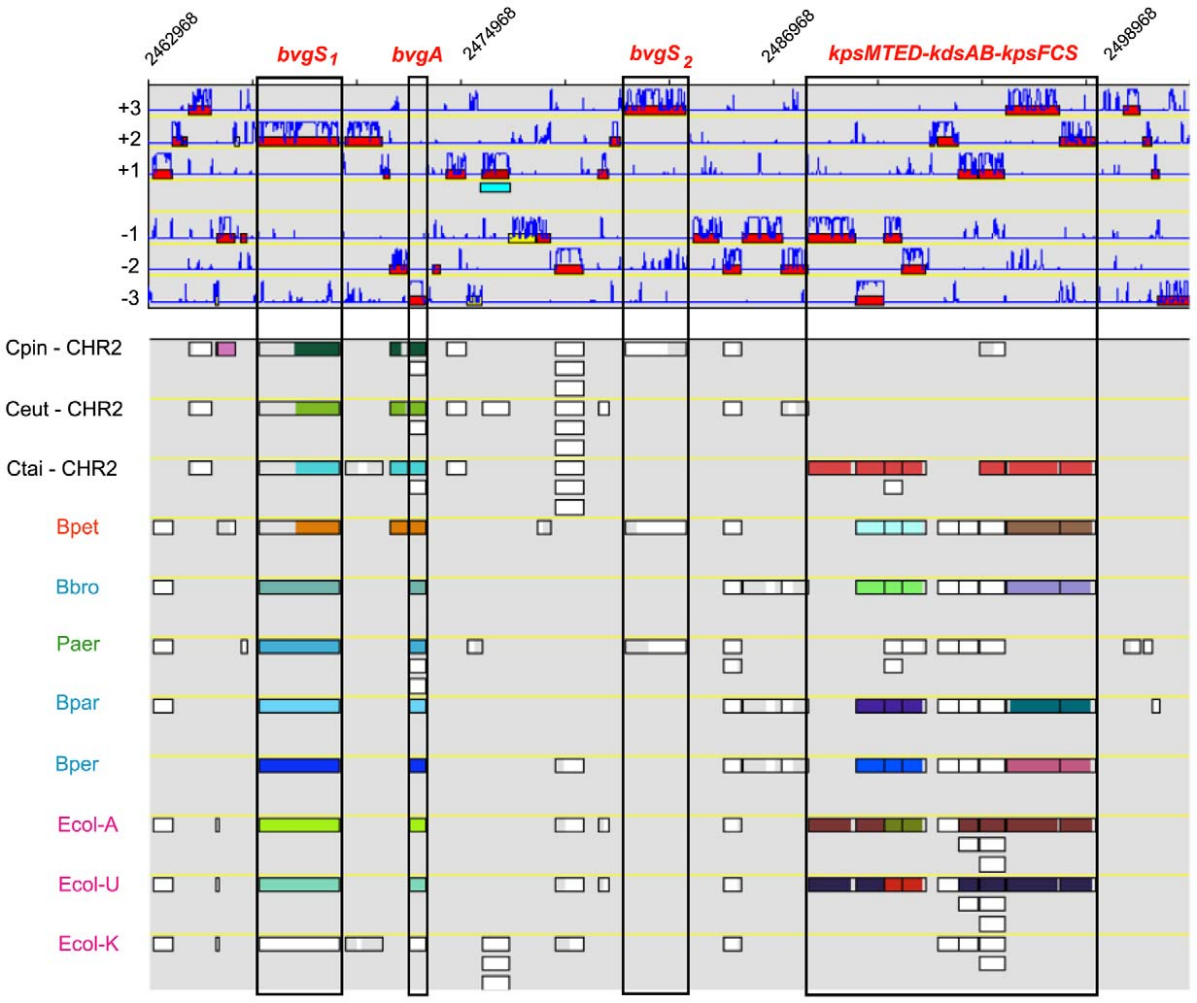
The *bvgSA* and *kps* loci of *C. metallidurans* CH34. Genomic region on the CHR2 replicon of *C. metallidurans* CH34 covering the *bvgSA* locus encoding a master virulence regulon (Rmet_5710, _5714, _5724) and the *kps* locus for synthesis and transport of capsular polysaccharides (Rmet_5729 to _5737). Relevant synteny maps are given for 11 organisms. Abbreviations: Cpin, *C. pinatubonensis* JMP134; Ceut, *C. eutrophus* H16; Ctai, *C. taiwanensis*; Bpet, *B. petrii* DSM 12804; Bbro, *Bordetella bronchiseptica* RB50; Paer, *Pseudomonas aeruginosa* PAO1; Bpar, *B. parapertussis* 12822; Bper, *B. pertussis* Tohama I; Ecol-A, avian pathogenic *Escherichia coli* (APEC01); Ecol-U, uripathogenic *E. coli* (UTI89); Ecol-K, *E. coli* K12. A nearby IS*1088* element (Rmet_5718) is indicated with a blue rectangle in the +1 reading frame (see text).

Another unusual feature of signal transduction in the *C. metallidurans* CH34 genome is the presence of an intact phosphorelay two-component system entailing a multidomain histidine-kinase sensor protein BvgS_1_ (Rmet_5710) and a response-transcriptional activator BvgA (Rmet_5714). This system BvgSA controls, via the transduction of environmental signals, the expression of numerous virulence- and colonization-factors in pathogenic *Bordetella* species [50], [151] and resembles, respectively, the EvgSA and RocSA systems in *E. coli* and *P. aeruginosa*. *E. coli* contains at least 37 EvgA-activated genes, including stationary-phase acid resistance genes and multidrug efflux pump genes [152], while *P. aeruginosa* RocA activates fimbrial adhesin genes for forming biofilms [153]. The BvgA transcriptional activator is well conserved in the four *Cupriavidus* species (63–68% aa identity) and *B. petrii* (58% aa identity), and displays a good similarity to EvgA and RocA, and to the BvgA regulators of pathogenic *Bordetella* species (39–45% aa identity) (all identities with minLrap >0.95). Likewise, the length and sequence of the *C. metallidurans* BvgS_1_ protein is conserved to EvgS and RocS, as well as to the BvgS sensory proteins of pathogenic *Bordetella* species (29–33% aa identity, minLrap 1.0). By contrast, the BvgS equivalents of *B. petrii* and the three other *Cupriavidus* strains are devoid of the large periplasmic domain spanning nearly 400 residues (Figure 6). Thus, only the BvgSA system of *C. metallidurans* is fully conserved in respect to the *E. coli* EvgSA, *P. aeruginosa* RocSA, and BvgSA systems of pathogenic *Bordetella*. Adding to the complexity of the BvgS structures in *C. metallidurans* and *B. petrii*, both have a second Bvg histidine-kinase sensor. It is called BvgS_2_ (encoded by Rmet_5724 and Bpet4472) and lacks the periplasmic domain. Also, in both organisms the phosphotransfer domain HPt, which is in pathogenic *Bordetella* part of the BvgS sensor, is an independent protein (Rmet_5713 and Bpet4470, respectively) (Figure 6). Certainly, the region spanning *bvgS_1_* (Rmet_5710) to *bvgS_2_* (Rmet_5724) has undergone multiple insertions and DNA rearrangements, proof of which is a nearby *IS*1088 copy (Rmet_5718) inserted into a defective serine protease gene (the IS element is indicated with a blue rectangle in the +1 reading frame of Figure 6).

### Capsular proteins

Immediately downstream of the *bvg* gene region (Rmet_5710-24; see above) there is a stretch of genes *kpsMTED-kdsAB-kpsFCS* putatively involved in synthesizing and transporting capsular polysaccharides (genes Rmet_5729 through Rmet_5737) (Figure 6). The *kdsA* and *kdsB* genes (Rmet_5733-34) located between these *kps* loci encode enzymes for the biosynthesis of 2-keto-3-deoxyoctulosonic acid (KDO), a common component of (lipo)polysaccharides. A similar gene casette is present in the *C. taiwanensis* genome as a cluster of nine genes (RALTA_B1091 through RALTA_B1100). Such gene casettes appear to be organised in three regions in analogy with the group II/III capsular polysaccharide gene clusters of K-type *Escherichia coli* [154], [155] in which a serotype-specific region (SSR) is flanked by conserved *kps* genes encoding a transport machinery for bringing polysaccharides to the cell surface. This entails a heterodimeric ABC transporter KpsMT, a multimeric KpsD outer membrane porter, and a periplasmic connector KpsE [156], [157]. The function of the other Kps proteins KpsF, KpsC, and KpsS is less clear but they were implicated in lipid metabolism, translocation and surface expression of capsule polymers, and determining chain length. We point out that the *C. metallidurans kpsF* gene (Rmet_5735), encoding a D-arabinose 5-phosphate isomerase, has a duplicate copy on CHR1 as gene Rmet_0307. Likewise, *kdsA* and *kdsB* have duplicate copies on CHR1, respectively, as Rmet_1053 and Rmet_0533. The genomes of *C. eutrophus* H16 and *C. pinatubonensis* JMP134 do not carry a *kps-*SSR*-kps* casette. In fact, of all the species sofar discussed in this paper, only pathogenic *Bordetella* species and *B. petrii* contain a similar casette *kpsMT-kpsE/wcbD-*SSR*-kpsCS/wcbAO* although they all seem to lack the *kpsD* gene encoding the outer membrane porter (in these species it may have been replaced by an unkown alternative protein). Interestingly, the plant symbiont *Sinorhizobium meliloti* produces a K-antigen that is exported by a typical *kpsMTED*-SSR-*kpsCS* module. This module is encoded in both *S. meliloti* strains Rm41 and 2101 by two separate *rkp* loci of which one is sited on the chromosome and the other one on the symbiotic megaplasmid [158], [159] (not shown).

Although there is no direct evidence for an K-antigen polysaccharide (KPS) in *C. metallidurans* CH34 or *C. taiwanensis*, such a Kps ‘secretin’ complex may be used to secrete other types of exopolysaccharides, lipo-oligosaccharides, or short-chained signaling molecules. In this context, the presence of a full set of *pelABCDEFG* genes (Rmet_4155 through Rmet_4561) in the *C. metallidurans* CH34 captured our interest. This *pel* operon first was described in *P. aeruginosa* PA14 as producing a glucose-rich biofilm matrix [160]; it later was identified in *P. aeruginosa* PAK and *R. solanacearum* GMI1000 [161]. Comparative analysis of related genomes via MaGe synteny maps (data not shown) failed to detect a *pel* locus in *C. pinatubonensis* JMP134; however, both *C. eutrophus* H16 and *C. taiwanensis* carry a full set of seven *pel* genes. Other genomes listed in the MaGe database with a complete set are those of three other *R. solanacearum* strains (Molk2, Ipo1609, and UW551), *R. picketti* 12D, *B. xenovorans* LB400, *Pseudomonas fluorescens* Pf-5, and *D. acidovorans*. The *pel* operon encodes sugar-processing enzymes, such as oligogalacturonide lyase (PelA), two glycosyltransferases (PelC and PelF), and a sucrose synthase (PelE), along with proteins involved in cellular signalling or transmembrane transport (i.e., PelD, a cyclic-di-GMP signal receptor and PelG, a putative ‘flippase’). Implication of the *pel* locus in biofilm formation was apparant by a variety of *pel* mutants of *P. aeruginosa* strains and *R. solanacearum* [160]–[162]. Immediately upstream of the *C. metallidurans* CH34 *pel* locus we identified three transposase (*tnpA*) genes belonging to different families of insertion elements namely Rmet_4154 (IS*Rme8*), Rmet_4153 (IS*Rme1*), and Rmet_4152 (IS*Rme5*) indicating a hotspot for insertion.

In searching for additional genes that might be involved in capsular protein synthesis and transport, we found two more regions of interest. The first is a large cluster of 20 genes (Rmet_2711 through Rmet_2731 situated on CHR1) clearly involved in lipopolysaccharide biosynthesis although the exact role of most proteins remains enigmatic. Based on the patched synteny with other genomes, seemingly this region, which is highly conserved compared to *C. taiwanensis*, to a lesser extent to the other *Cupriavidus* and *Ralstonia* species, and very intermittantly with the genomes of any other of the species in the MaGe database, displays an amalgam of genes encoding secretion proteins and sugar-processing enzymes, possibly of different origins, that may or may not act in concert. A second cluster of nearly 30 genes (Rmet_5832 through Rmet_5860 situated in CHR2) exhibits a similar mosaic structure with many genes being hypothetical or conserved hypothetical while others generally seem to be involved in transport, signal transduction, or sugar processing activities. Some genes are worth mentioning, for instance Rmet_5835 and Rmet_5843, encoding proteins that respectively display a fair- to-good aa identity with the *P. aeruginosa* PslA (32%) and PslB (43%) proteins. Psl proteins in *P. aeruginosa* are implicated in the synthesis of a mannose-rich component of the biofilm matrix [162]. Just downstream of these genes, on the opposite strand of CHR2, lies a small cluster of three genes *epsAPB* (Rmet_5851-5850-5849) encoding proteins involved in exporting exopolysaccharides; this locus is, on the same strand, immediately preceded by four genes encoding glycosyltransferases (Rmet_5853 through Rmet_5856), and two more genes encoding sugar-processing enzymes mannose epimerase (Rmet_5859) and mannose dehydratase (Rmet_5860). We believe that these two large regions (in addition to the better defined *pel* locus) deserve special attention in regard to the *kps*-SSR-*kps* casette or alternative transport systems encoded by the *C. metallidurans* CH34 genome, particularly in view of the organism's association with biofilms [14], [39], and the role of exopolysaccharides in bacterial complexation of metals [163].

### Motility, chemotaxis, biofilms, and Q-sensing

*C. metallidurans* displays flagella uniformly distributed over the cell surface [34] allowing it to swarm and swim. Most of the *flh*, *fli*, and *flg* genes necessary for synthesizing flagella are grouped within four gene clusters: A *flh* locus Rmet_3698-3703, two *fli* loci Rmet_5252-66 and Rmet_5298-5303, and the *flg* locus represented by Rmet_3731-44. In addition, the CH34 genome encodes *cpa*, *tad*, and *pil* loci essential for synthesizing type IV pili (Tfp), allowing cells to move across solid surfaces via a mode of twitching. These include the *pilVWX* genes (Rmet_0189/91), various *cpa* and *tad* genes within the region Rmet_0639 through Rmet_0697, *pilTU* genes (Rmet_2935/6), the *pilBCD* genes (Rmet_3108-10), the *pilMNOPQ* genes (Rmet_3268-72), and the *cpaBCD-tadABCD* genes (Rmet_3653-59). The CH34 genome also contains many genes required for the biogenesis of fimbriae important for cell adhesion, colonization, and biofilm formation, including the *fimAC-htrE* genes Rmet_0574/5/6, FimA-type adhesin genes Rmet_1667, Rmet_4250, and Rmet_4961, and a *fim* locus Rmet_4250-64. Our genome survey shows that more than 150 genes may be involved in generating or controlling flagella, pili, and fimbriae. Most occur within the above loci, or are situated within a large cluster of 18 chemotaxis (*che*), motility (*mot*) and flagellar (*flh, fli*) genes (Rmet_3678 through Rmet_3695); others are part of smaller sets of genes encoding sensory- or regulatory-proteins.

One major mechanism of regulating motility in strain CH34 is through the action of a Quorum Sensing (QS) system encompassing the *phcBSRQ* operon (Rmet_2949-52) and the *phcA* gene (Rmet_2977) [139]. This QS or cell-to-cell communication system was first discovered in *R. solanacearum* and is neither related to the classical LuxIR system utilizing acyl-homoserine lactone (AHL) autoinducers nor the LuxS system with autoinducer-2. In *R. solanacearum*, PhcB directs the synthesis of the diffusible signal molecule 3-OH-palmitic acid methyl ester (3-OH-PAME). Typically, with higher cell densities also 3-OH-PAME levels increase to an extent that it saturates the membrane-bound sensor PhcS, effectively lifting the PhcR-mediated repression of the PhcA regulator. All five *phc* genes of this system are conserved in CH34, the other three *Cupriavidus* species, and *R. pickettii*. However, while 3-OH PAME could be extracted and purified from *R. solanacearum*, this was not the case for CH34, indicating that 3-OH PAME-like activity in CH34 may result from a structural variant of this molecule [139]. In the plant-pathogenic bacterium *R. solanacearum* activation of the virulence regulator PhcA is followed by an enhanced production of exoenzymes and extracellular polysaccharides leading to severely decreased motility, presumably in preparation for host colonization [164]. This is not the case in strain CH34 where PhcA activation actually stimulates motility [139]. Likewise, the inactivation of *phcA* in *R. solanacearum* increased siderophore production tenfold [135] but not in *C. metallidurans* CH34 where no affect on siderophore production was seen [139]. Possibly, the Phc regulatory system may connect to different sets of genes in *C. metallidurans* compared to *R. solanacearum*; this is not unduly surprising bearing in mind the fundamental differences between their ecology and physiology. Interestingly, CH34 does possess an AHL acylase (Rmet_4680), which enables CH34 to modulate AHL levels and thereby AHL-dependent regulatory systems of other members in a multispecies microbial community.

### Heterotrophic carbon metabolism

*C. metallidurans* CH34 cannot grow on glucose, fructose, or galactose [9] because it lacks adequate glucose uptake systems and does not possess a functional 6-phosphofructokinase. Consequently, energy is not produced through the Embden-Meyerhof-Parnas pathway, but by assimilating acidic sugar-derivatives such as D-gluconate, acetate, pyruvate, lactate, L-malate, benzoate, 4-hydroxybenzoate, and various dicarboxylic acids (C_4_ to C_11_) [9]. CH34 can derive glucose-6-phosphate from gluconeogenesis (see below) or via the phosphorylation of cytoplasmic glucose by glucokinase (Rmet_5799). It then is further converted to 6-phosphogluconate (6GP) in a two-step reaction involving the *pgl* and *zwf* genes (encoded by Rmet_5800 and Rmet_5801, respectively). The 6GP is metabolized through the alternative glycolytic Entner-Doudoroff (ED) pathway involving a 6-phosphogluconate dehydratase (EDD; Rmet_5802) and a 2-keto-3-deoxy-6-phosphogluconate aldolase (EDA; Rmet_4768), thus generating two pyruvate molecules that finally are processed to acetyl-CoA. In many prokaryotes, this acetyl-CoA is fully oxidized to CO_2_ via citrate, D-isocitrate, 2-ketoglutarate, succinate, fumarate, malate, and oxalacetate in the tri carboxic-acid (TCA) cycle, that is, the Krebs cycle. However, *C. metallidurans* apparantly also can use the glyoxylate bypass in which D-isocitrate is cleaved by isocitrate lyase (AceA) (Rmet_1385, Rmet_4737) into succinate and glyoxylate. As in full TCA cycles, succinate is converted to fumerate by succinate dehydrogenase (succinate:quinone reductase, SQR), a multimeric enzyme encoded by four genes *sdhCDAB* (Rmet_2483/4/5/6) and further processed to malate by fumarase FumA (Rmet_2273, Rmet_5752). In parallel, glyoxylate condenses with acetyl-CoA yielding malate via malate synthase (AceB) (Rmet_1390). Malate finally is changed into oxaloacetate via the malate dehydrogenase enzyme MDH (Rmet_2489, Rmet_5095), completing a full cycle in which acetate is again transformed to citrate by citrate synthase CitA (Rmet_2481, Rmet_4144, Rmet_4268, Rmet_4946, and Rmet_5380) and citrate to D-isocitrate via aconitase AcnA (Rmet_1585, Rmet_2492, Rmet_4240, and Rmet_5296). We note that D-isocitrate, as for full TCA cycles, is transfigured into 2-ketoglutarate by isocitrate dehydrogenase ICDH (Rmet_2895, Rmet_3729). The carbon skeleton of 2-ketoglutarate then is used, depending on cellular ammonia levels and energy status, by either one of the two enzymes glutamate dehydrogenase GDH (Rmet_1181) or glutamate synthase (GOGAT) (GltAB encoded by Rmet_3262/3) to generate glutamate that in turn feeds the nitrogen metabolic pathways. We offer three important observations here. First, although the *C. metallidurans* CH34 genome encodes the E1, E2, and E3 components of an α-ketoglutarate dehydrogenase (KGDH) complex (Rmet_2048 through Rmet_2050), their role in the TCA cycle for converting 2-ketoglutarate to succinate via succinyl-CoA is undecided. Possibly, an alternative route is followed wherein the gene products of Rmet_2049 and Rmet_2050 actually form a ketoglutarate decarboxylase (KGD) complex that changes 2-ketoglutarate into the intermediate succinate semialdehyde followed by further conversion to succinate by a succinate-semialdehyde dehydrogenase (SSDH) encoded by the *gabD* genes (Rmet_1898, Rmet_3961, and Rmet_4942). Interestingly, these SSDH enzymes may have a role in 4-hydroxybutyric acid catabolism, as was reported for *C. eutrophus* H16 [165]. Second, neither succinate nor oxaloacetate are produced via a reductive TCA cycle as the essential enzymes are missing (see further). And third, most TCA enzymes in *C. metallidurans* CH34 appear to have multiple isoenzymes whose genes are scattered throughout both replicons CHR1 and CHR2 (i.e., we discerned at least five citrate synthase genes, four aconitase genes, three genes for SSDH, and two genes each for ICDH, MDH, fumerase, and isocitrate lyase).

None of the sequenced *Cupriavidus* strains contains a gene for 6-phosphogluconate dehydrogenase (6-PGDH), basically ruling out the oxidative branch of the pentose-phosphate pathway. Nonetheless, all essential genes for gluconeogenesis, in which glucose is generated from non-sugar carbon substrates (such as pyruvate, lactate, oxaloacetate, and glycerol) and glucogenic amino-acids (primarily alanine and glutamine) are present in *C. metallidurans*. They include genes to transform malate via oxaloacetate (Rmet_2489, Rmet_2750) or via pyruvate (Rmet_0878, Rmet_1453) to phosphoenolpyruvate that in turn is processed to fructose-1,6-biphosphate through a cascade of enzymatic actions by, for example, phosphopyruvate hydratase (ENO; Rmet_1055, Rmet_1176), phosphoglycerate mutase (PGM; Rmet_0251, Rmet_0420), phosphoglycerate kinase (PGK; Rmet_0501, Rmet_1516), glyceraldehyde-3-phosphate dehydrogenase (GAP; Rmet_1096, Rmet_1515, Rmet_2979), and fructose-1,6-biphosphate aldolase (FBA; Rmet_0503, Rmet_1492, Rmet_1518)]. The resulting fructose-1,6-biphosphate is further converted to fructose-6-phosphate by fructose-bisphosphatase (FBP; Rmet_0875, Rmet_1507, Rmet_1511) and finally into β-D-glucose-6-phosphate by glucose 6-phosphate isomerase (GPI; Rmet_1879). Without exception, CHR1 encompasses all the genes required for gluconeogenesis, some of which also are involved in the reductive pentose-phosphate pathway (Calvin-Benson-Bassham cycle).

Under aerobic conditions, *C. metallidurans* CH34 potentially can degrade benzoate and 4-hydroxybenzoate to ketoadipate via catechol and protocatechuate, respectively, as it has all the genes for both ortho-cleavage pathways (e.g., the *benABCDM* and *catABCD* genes Rmet_4875 through Rmet_4885, *pcaA* gene Rmet_4270, *pcaBCDHGKQ* genes Rmet_4011 through Rmet_4016, and *pobAR* genes Rmet_4017/8). The ketoadipate thus formed then is processed to acetyl-CoA by the products of the *pcaIJF* genes (Rmet_3667/8/9). In strain CH34, benzoate might be degraded anaerobically via the central metabolite benzoyl-CoA (instead of catechol; see Harwood *et al.* [166]) in an alternate pathway requiring the products of Rmet_1220 through Rmet_1225. Therein, the benzoyl-CoA reductase BoxAB (Rmet_1220/1) will open the benzene ring, followed by β-oxidation of the resulting product (oxidation occurs at the β-carbon C3) leading to 3-ketoadipyl-CoA, and finally to succinyl-CoA and acetyl-CoA. Five more genes of this operon (Rmet_1226 through Rmet_1230) probably encode an ABC-like transport system of benzoate.

Similarly, β-oxidation may play an important role in the formation of acetyl-CoA from dicarboxylated compounds because reportedly, *C. metallidurans* CH34 grows on a range of dicarboxylated compounds [9]. However, without experimental evidence, it is difficult to predict the genes and enzymes that would be involved in these conversions in CH34. An interesting report by Harrison and Harwood [167] implicated the *pimFABCDE* operon for the β-oxidative degradation of C_7_-C_14_ dicarboxylic- and fatty-acids in *Rhodopseudomonas palustris.* This operon encodes a set of acyl-CoA ligase, enoyl-CoA hydratase-, acyl-CoA dehydrogenase-, and acyl-CoA transferase-enzymes, and is accompanied by a IclR-type regulatory gene, and several genes for ABC-like transport. Our search through the MaGe annotated data of CH34 yielded more than a hundred of these enzymes. Though most of them might degrade fatty acids, we believe that in CH34 many others are recruited to specifically degrade dicarboxylates.

### Aerobic- and anaerobic-respiration

The citric-acid cycle is followed by oxidative phosphorylation in which NADH, FADH_2_, and GTP are reduced by cytochromes and other terminal oxidases in the cell's inner membrane, while protons are expelled into the periplasm. The resulting proton gradient across the membrane then drives ATP synthesis by the conversion of ADP (ATPase; α, β, γ, δ, ε, a, b, and c units encoded by Rmet_3493 through Rmet_3500). A key locus in this aerobic energy metabolism is a 14-gene cluster located on CHR1 (Rmet_0927 through Rmet_0940) encoding a large membrane-bound complex of NADH dehydrogenase (NADH:quinone oxidoreductase) that is highly conserved within the four sequenced *Cupriavidus* strains, and in other closely related species. According to BioCyc pathway analysis, alternative NADH dehydrogenases may exist in *C. metallidurans*. These are encoded by Rmet_2623 as a single-subunit FAD-binding NDHase, by Rmet_0177 as a NAD-dependent epimerase/dehydratase, and by Rmet_4310 as a FAD-dependent pyridine nucleotide-disulphide oxidoreductase. We also discerned the Rmet_4300 gene that apparently codes for a NuoF homologue (NADH ubiquinone oxidoreductase, chain F). Other membrane-associated protein complexes that may be important to the electron transport chain in strain CH34 are succinate:quinone reductase (SQR; see previous section on the TCA cycle), a *bc_1_*-type cytochrome complex (encoded by Rmet_3228/9/30), a *cbb_3_*-type cytochrome c oxidase (encoded by *cco* genes Rmet_2041/2/3/4), two cytochrome *bd*-type quinol oxidases CydAB (encoded by the genepairs Rmet_4955/6 and Rmet_5232/3), a *aa_3_*-type oxidase encoded by the *cta*-gene cluster (Rmet_0261 through Rmet_0271), a *bb_3_*-type cytochrome oxidase encoded by the *cox*-gene cluster (Rmet_3955/6/7/8/9), and three quinol oxidases encoded by the clusters *cyo1* (Rmet_0948/49/50/51), *cyo2* (Rmet_5608/09/10/11), and *cyo3* (Rmet_5790/1/2/3).

Under anoxic conditions, *C. metallidurans* CH34 may resort to alternate modes of energy generation by using electron acceptors other than oxygen. Thus, the bacterium reduces nitrate [32], which is reflected by three loci encoding distinct types of nitrate reductases: a cytoplasmic assimilatory enzyme (NAS; encoded by the *nasAB* genes Rmet_4820/1), a membrane-bound quinol-oxidizing enzyme (NAR; encoded by the *narGHJI* genes Rmet_2074 through Rmet_2077, accompanied by two ABC-type transporter-genes Rmet_2072/3), and a periplasmic enzyme (NAP; encoded by the *napEDABC* genes Rmet_4061 through Rmet_4065). Probably, the NAS-, NAP-, and NAR- systems have different physiological roles depending on metabolic conditions. Consequently, their pathways may be interconnected to foster rapid adaptation to changing nitrogen and/or oxygen conditions, a big asset for survival in nature.

Although strain CH34 reportedly does not reduce nitrite [32], it contains two genes Rmet_4018/9 encoding the large- and small- subunits of a putative assimilatory nitrite-reductase (ANR). These genes, preceded by a nitrate transporter gene (Rmet_4817), lie immediately upstream of the NAS genes (Rmet_4820/1). Theoretically, full denitrification by the successive reduction of nitrate to molecular nitrogen seems possible in CH34 because of three key genes. They are the *nirS* gene (Rmet_3172) encoding a NO-forming nitrite-reductase (NNR), the *norB* gene (Rmet_3167) encoding a nitric-oxide reductase (NOR), and a *nosZ* gene (Rmet_4917) coding for a nitrous-oxide reductase (NOS). These three are centered among additional genes that might be required for functional denitrification: (i) The *nirENJ* (Rmet_3168/69/70) and *nirCFGH* genes (Rmet_2079/80/81, RALME2203), (ii) the *norR* gene (Rmet_3166) encoding a σ^54^ dependent transcriptional regulator, and, (iii) the NOS accessory genes *nosLYFDR* (Rmet_4912/3/4/5/6) and *nosCX* (Rmet_4918/9). A regulatory two-component system NarX-NarL that may be important for nitrate- or nitrite- controlled transcription is encoded by Rmet_2086/7.

In view of the ability of strain CH34 to metabolize anaerobically and its potential for full denitrification, we note that it has all the genes for the three known classes of ribonucleotide reductase (RNRase): NrdAB (Rmet_3087/8), NrdJ (Rmet_2130), and NrdDG (Rmet_5137, Rmet_5139), along with NrdR (Rmet_2681) as a transcriptional repressor of these genes. According to the RNRase database (http://rnrdb.molbio.su.se/) less than 8% of all bacteria have all three classes of RNRase: *C. eutrophus* H16 and C. *metallidurans* CH34 are among the bacteria with these genes, but *C. pinatubonensis* JMP134 and *C. taiwanensis* lack the *nrdDG* genes. Our identification of *nrdDG* genes in strain CH34 is important because the NrdDG enzyme, which operates only under anaerobic conditions, is essential for complete denitrification in *C. eutrophus* H16 [168]. As the latter's *nrdDG* genes lie on the pHG1 plasmid, a pHG1-free mutant strain grows very poorly on nitrate and does not achieve full denitrification. This defect in the mutant is countered by adding cobalamin (VitB_12_) to the medium [169], most likely because the coenzyme B_12_-dependent ClassII RNRase, NrdJ, assumes the role of NrdDG, while at the same time *C. eutrophus* H16 readily takes up and assimilates cobalamin. Likewise, *C. metallidurans* CH34 encodes most, if not all genes for the uptake and assimilation of cobalamin (see next section). Together, these facts strongly indicate that anaerobic metabolism, with nitrate as the terminal electron-acceptor, runs through very similar pathways in strains H16 and CH34.

### Vitamin biosynthesis

*C. metallidurans* CH34 has multiple genes for assimilating a variety of B family vitamins, or their *de novo* production, including thiamin (VitB1), riboflavin (VitB2), pantothenic acid (VitB5), pyridoxal 5′-phosphate (VitB6), biotin (VitB7), folic acid (VitB9), and cobalamin (VitB12). These vitamins, important cofactors for synthesizing fatty acids, proteins, carbohydrates, and branched-chain amino acids, aid in electron-relay systems and energy production. In our genomic survey for vitamin-related genes we did not find any that are relevant to the biosynthetic pathways of retinol (VitA), niacin (VitB3), ascorbic acid (VitC), calciferol (VitD), tocopherol (VitE), or phylloquinone (VitK).

Thiamin (VitB1) biosynthesis is likely governed in *C. metallidurans* CH34 by the *thiCOSGED* locus (Rmet_0162/6, Rmet_0169). This set of genes is needed to generate the thiazole- and pyrimidine- moieties of the vitamin as well as to control the condensation reaction of both moieties to form thiamin phosphate. Additional genes involved in thiamin biosynthesis are *dxs* (Rmet_2615), *icsS* (Rmet_1025), and *thiL* (Rmet_3047) encoding an oxidase and catalytic enzymes. Two more kinases, ThiF and ThiK, usually are involved in thiamin synthesis but we identified only three putative *thiF* genes (Rmet_0249 and Rmet_2639, both located on CHR1, and RALMEp10091, present on the pMOL28 plasmid); we did not recognize the *thiK* gene.

The biosynthesis of riboflavin (VitB2) in CH34 might be linked to the genes *ribDC* (Rmet_2688/9), *rib(BA)E* (Rmet_2693/4), *ribA1* (Rmet_0194), *ribF* (Rmet_2884), *ribA2* (Rmet 4572), and *ribB* (Rmet_4758). No other *rib* genes were detected.

Besides its involvement in energy metabolism, pantothenic acid (VitB5) is regarded as a crucial component of coenzyme A biosynthesis, starting from β-alanine and L-valine. In *C. metallidurans* CH34, the synthesis of VitB5 could be connected to genes *ilvE* (Rmet_0493), *panB* (Rmet_2774), *panC* (Rmet_2917), and two copies of *panE* (Rmet_4536, Rmet_5770).

The biosynthesis of pyridoxal 5′-phosphate (VitB6) in this strain proceeds via two complementary pathways. The biosynthetic pathway involves the genes *pdxC* (*serC*; Rmet_0715), *pdxA* (present in two copies Rmet_0438, Rmet_2590), a gene encoding an FMN-binding protein (Rmet_0820), *pdxJ* (Rmet_2415), *dxs* (Rmet_2615; also involved in VitB1 synthesis, see above), *pdxH* (Rmet_2642), and a putative pyridoxine oxidase gene (Rmet_4050). We did not detect the genes involved in generating 2-oxo-3-hydroxy-4-phosphobutanoate, an early step in VitB6 sysnthesis and the actual substrate of the SerC catalytic action. The second pathway, the salvage pathway, basically converts pyridoxine, pyridoxamine, or pyridoxal, into pyridoxal-5′-phosphate, the active form of VitB6. This involves only two genes *pdxH* (Rmet_2642) and *pdxK* (Rmet_4114). We note that the former also is part of the *de novo* biosynthetic pathway (above).

The *bioAFDB* locus (Rmet_0114/5/6/7) in CH34 controls the production of biotin (VitB7). There are slight variations in the pathway, depending on whether pimelate or pimeloyl-CoA is the starting substrate; nonetheless, the geneproducts of all four genes are involved in both. Pimeloyl-CoA may be derived from the conversion of aromatic compounds via the intermediates benzoyl-CoA and 3-hydroxypimelyl-CoA, while the direct use of pimelate necessitates a fifth gene, *pauA* (pimelic acid-utilizing A; Rmet_2598). The *pauA* gene codes for a 6-carboxyhexanoate-CoA ligase (often referred to as a ‘pimeloyl-CoA synthetase’), essentially synthesising pimeloyl-CoA from pimelate and coenzyme A.

The variant pathways and intermediate reactions leading to the formation of folate (VitB9) from chorismate or GTP are complex and behind the scope of our genome study. We identified several genes that might be involved in folate biosynthesis in *C. metallidurans* CH34; they are scattered throughout the genome and linkage between individual ones is blurred. These genes are *folB* (Rmet_0183), *folD* (Rmet_1192), *purT* (Rmet_1820), *folP* (Rmet_2187), *folC* (Rmet_2463), *folA* (Rmet_2569), *purN* (Rmet_2878), *folK* (Rmet_2915), *trpG* (Rmet_3179), and a putative *folE* (Rmet_3990). We failed to identify a hydrolase gene for converting 7,8-dihydroneopterin triphosphate into 7,8-dihydro-D-neopterin.

There have been extensive studies on cobalamin, a vitamin of a particular interest in public health and medical sciences, and produced only by microorganisms [170]: indeed, its biosynthesis is one of the best known vitamin pathways to date [171]. Two pathways for corrin ring formation were described in bacteria, differing from each other in the way cobalt is incorporated [172]. In *C. metallidurans* CH34, the presence of a sirohydrochlorin cobaltochelatase gene *cbiX* (Rmet_2810) suggests that this organism synthesises cobalamin anaerobically, thereby including an early insertion step of cobalt. Genes involved in the late stage of the biosynthesis are grouped in a large operon of 13 genes (entailing *cbi*, *cob* and *btu* genes Rmet_2777 through Rmet_2789) with a cobalamin riboswitch we identified at the far end (Rmet_R0047). This entire operon, which according to the MaGe interface displays good synteny with all *Cupriavidus* and *Ralstonia* genomes, contains all the essential genes for producing adenosylcobalamin from cobyrinic acid (including those controlling corrinoid transport, viz. *btuB*, *btuC*, *btuD*, and *btuF*). Only the *bluB* gene (Rmet_2213), coding for a cobalt reductase, is at a different location on CHR1. Whilst the early and late stages of the biosynthesis generally are covered by these listed genes, other genes involved in the intermediate corrin biosynthesis in *C. metallidurans* await elucidation.

### Autotrophic metabolism

In the absence of heterotrophic carbon sources, *C. metallidurans* CH34 can switch to an autotrophic lifestyle by fixing and reducing carbon dioxide for synthesizing organic molecules. Generally, autotrophic prokaryotes have four different biochemical routes to accomplish this (reviewed by Atomi [173]): (i) The reductive pentose-phosphate pathway; (ii) the reductive acetyl-CoA pathway; (iii) the 3-hydroxypropionate cycle; and, (iv) the reductive tricarboxylic-acid (rTCA) cycle.

The *C. metallidurans* CH34 genome encodes all enzymes necessary for the 13 biochemical reactions of the reductive pentose phosphate pathway, also known as the Calvin-Benson-Bassham (CBB) cycle. Figure S6 is a schematic view of the *cbb* loci in CH34, using Tabita *et al.* 's gene notations [174]. We note especially that this region contains some IS elements (marked in red by their *tnpA* transposase genes in Figure S6), including two exterior IS*1071* elements, Rmet_1491 and Rmet_1544. This may explain the earlier observed loss of autotrophic growth in CH34 under mutagenic stress [22] since the IS*1071*-mediated excision of this region would remove, amongst other genes, the two structural genes *cbbL* (Rmet_1501) and *cbbS* (Rmet_1500) of RubisCo (ribulose 1–5 bifosfaat carboxylase oxygenase) as well as of the accessory genes *cbbQ* (Rmet_1499) and *cbbO* (Rmet_1498) needed to assemble RubisCo.

Several regulators control the CBB cycle. First, there are three copies of *cbb*R, a LysR-type transcriptional activator of *cbb* gene expression. Two copies juxtapose the central *cbb* gene clusters, *cbb*R1 (Rmet_1502) and *cbb*R2 (Rmet_1508), while a third copy, *cbb*R3 (Rmet_4365), is located on the second replicon CHR2 (unlike other *cbb* genes that all are sited on CHR1). Furthermore, the *C. metallidurans* CH34 genome harbours a set of *reg*A (Rmet_0135) and *reg*B (Rmet_0136) genes probably regulating carbon fixation via the CBB-cycle, as in other organisms [173], [175]. Remarkably, three non-essential *cbb*-genes appear to be transcriptionally linked to the *cbb* operon: *cbb*Y (Rmet_1517), *cbb*Z1 (Rmet_1514), and *cbb*Z2 (Rmet_3177). Under certain conditions, autotrophic bacteria carrying these exceptional *cbb* genes display a phosphoglycolate phosphatase (PGP) enzyme activity [176] that may help cells to cope with toxic levels of phosphoglycolate accumulated by the oxygenase reaction of RubisCo whilst the carbon skeleton of phosphoglycolate is salvaged as glycolate for further use in glycine synthesis. We did not detect a *cbbX* gene in the *C. metallidurans* CH34 genome.

Besides the CBB cycle, *C. metallidurans* CH34 apparantly has functional genes to fix carbon dioxide via the reductive acetyl coenzyme A pathway (also termed the Wood-Ljungdahl pathway). One key biochemical reaction in this pathway is the transition between carbon dioxide and carbon monoxide mediated by a metalloenzyme, CO dehydrogenase (CODH). This enzyme is a dimeric heterotrimer consisting of a small, medium, and large subunit representing respectively, an iron-sulfur protein (CoxS), a flavoprotein (CoxM), and a molybdoprotein (CoxL). Two forms of aerobic CO-DH are known: Form I was characterized explicitally for its ability to oxidize carbon monoxide, but the function of form II CODH, distinct from form I but phylogenectically closely related, remains unclear [177]. Strain CH34 carries the genes *coxSLM* (Rmet_0365 – Rmet_0363) and the accessory genes *coxDEGI* (Rmet_0362 – Rmet_0359). The order of the CODH genes in this operonic structure and the presence of an AYRGAGR motif in CoxL (encoded by Rmet_0364) strongly suggest that the *C. metallidurans'* CODH is of form II. Although the ability of *C. metallidurans* CH34 to undertake substantial oxidation of carbon monoxide still needs to be tested, CODH-II-encoding *cox* genes were found in *Bradyrhizobium japonicum* USDA 110, an organism relying solely on CODH-II for CO oxidation and hence, growth on carbon monoxide as a sole carbon- and energy- source [178].

Based on our genomic survey and supported by KEGG analysis, *C. metallidurans* CH34 lacks the cycle-specific malonyl-CoA reductase (EC:4.1.1.9), an essential enzyme for the 3-hydroxypropionate cycle [179], [180], and hence, cannot employ this pathway to assimilate carbon dioxide. Likewise, the *C. metallidurans* CH34 genome does not code for the key enzymes of a rTCA cycle, i.e., ATP-citrate lyase and 2-oxoglutarate synthase nor does it encode enzymes for alternative formation of oxaloacetate, for example, via the combined action of citryl-CoA synthetase and citryl-CoA lyase, thus effectively ruling out the rTCA-cycle as a route for fixing carbon dioxide.

From this genomic information it appears that *C. metallidurans* CH34 relies primarily on a RubisCO Type I enzyme for fixing carbon dioxide as part of a complete CBB-cycle. Although an intact CODH-II based system is present, it is yet unclear whether strain CH34 can use CO as a sole energy and carbon source.

### Sulfur oxidation (sulfur autotrophy)

*C. metallidurans* CH34 has the genetic capacity to oxidize inorganic sulfur compounds by the action of sulfur oxidizing genes (*sox*) and sulfur oxygenase reductase genes (*sor*) (Figure S7A) [181]. The first set of genes comprises the *soxBWXAZYFE* gene cluster situated on CHR1 as Rmet_3417 through Rmet_3427. Basically, sulfur respiration requires only five *sox* genes whose products form three key periplasmic protein complexes: SoxYZ, a sulfur carrier protein, SoxXA, a c-type cytochrome complex, and SoxB, a sulfate thiol hydrolase. In this system, the oxidation of thiosulfate (S_2_O_3_^2−^) to sulfate entails the oxidative linkage, mediated by SoxXA, of thiosulfate to the conserved C-terminal cysteine of SoxY, followed by hydrolysis of the terminal sulfone (SO_3_^−^_)_ group by SoxB (Figure S7B). The SoxXA complex then covalently binds other pathway intermediates to the SoxY cysteine thus forming extended chains of sulfane (S^−^) groups preceding the terminal sulfone group (SO_3_^−^) [182]. These sulfane chains are broken by the iterated actions of the SoxB hydrolase and a periplasmic sulfur dehydrogenase, SoxCD. Although *C. metallidurans* CH34 lacks the *soxCD* genes, this would not impair its ability to oxydise thiosulfate to sulfate in the first step of the cycle. However, fewer electrons would be liberated compared to the electron generation of complete *sox* systems.

In *C. metallidurans* CH34, the likely route for sulfide (HS^−^) oxidation would be via the *soxE* and *soxF* gene products, so forming a typical c-type flavocytochrome complex in which SoxF functions as a sulfide dehydrogenase (Figure S7C). Lastly, the *C. metallidurans sox* gene cluster includes two genes of unknown function coding for a conserved hypothetical protein (Rmet_3421), putatively exported, and an unknown c-type cytochrome precursor (Rmet_3424).

*C. metallidurans* probably oxidizes sulfites (SO_3_^−^) through the action of the *sorA* and *sorB* genes (present on CHR2 as Rmet_4891 and Rmet_4892) (Figure S7D) that are orthologues of the *C. eutrophus* H16 *sorA* and *sorB* genes encoding, respectively, a periplasmic sulfite dehydrogenase and a membrane-bound c-type cytochrome [71].

These routes of sulfur oxidation (SoxB-XA-YZ, SoxEF, and SorAB) in combination with an autotrophic lifestyle would enable *C. metallidurans* to use reduced sulfur compounds as electron donors, while employing carbon dioxide as an electron acceptor, and hence it could function as a true chemolithotrophic sulfur-oxidizing bacterium. Such a sulfur-based growth is sustainable only when sufficient amounts of thiosulfate ions are present. A biological source for them is from the fermentation of taurine (2-aminoethanesulfonate), together with the potential fermentation of other organosulfonates in anoxic environments [183]. Interestingly, *C. metallidurans* thrives not only on thiosulfate but also on a wide range of aromatic sulfonates, alkanesulfonates, and sulfate esters [184]. Therefore, it is not surprising that the *C. metallidurans* CH34 genome encodes alkanesulfonate- and taurine-specific ABC transporter systems, such as SsuABC (Rmet_1370/2/3) and TauABC (Rmet_2857/8/9), an alkanesulfate monooxygenase SsuD (Rmet_1371), at least six copies of taurine dioxygenase TauD (encoded by Rmet genes _4136, _4409, _4633, _5119, _5292, and _5823), a reductase-ferredoxin AsfA/AsfB (Rmet_4334/5), and a putative NAD(P)H-dependent FMN reductase SsuE (Rmet_5075) that with AsfA/AsfB may form an electron chain for aromatic desulfonation. We did not identify other genes for enzymatic desulfonation from the *C. metallidurans* CH34 genome data.

### Hydrogen oxidation (hydrogenotrophy)

*C. metallidurans* CH34 can gain its energy from oxidising hydrogen gas (H_2_) into electrons (e^−^) and protons (H^+^). The underlying enzymatic engine has two parts: A periplasmic membrane-bound hydrogenase (MBH), and a cytosolic soluble hydrogenase (SH). This allows the bacterium to switch between an H_2_-driven energy supply and a classic lithotrophic growth mode. In contrast to the H_2_-oxidising *C. eutrophus* H16, the expression of MBH and SH in *C. metallidurans* CH34 is not controlled by an H_2_-sensor in the form of a third hydrogenase. Therefore, the nearby concentration of hydrogen gas does not regulate the lithotrophic growth of CH34. Nonetheless, the lack of a specific H_2_-sensor-hydrogenase does not entail the constitutive expression of the energy-related hydrogenases, such as in the absence of H_2._ Rather, both the hydrogenases and the RubisCo enzyme (see autotrophic metabolism) are regulated by the carbon source afforded by the growth substrate [9].

All the genes needed to form and activate MBH and SH are grouped in two large gene clusters situated on CHR1 (Figure S8A). The first cluster, governing MBH, harbors the *hy*drogenase *p*leiotropic operon encompassing six genes *hypE_1_D_1_C_1_F_1_B_1_A_1_* (Rmet_1281 – Rmet_1286), followed by a set of genes required for *h*ydrogen *ox*idation, *hoxVTRQOLMZGK* (Rmet_1287 through Rmet_1293, Rmet_1295, and Rmet_1297-98). This entire cluster is located on genomic island CMGI-2. A second cluster, governing the SH, contains one partial *hox* gene denoted Δ*hoxV* (Rmet_1534), nine intact *hox* genes *hoxFUYHWINXA* (Rmet_1522 through Rmet_1527, Rmet_1533, and Rmet_1541-42), and six genes *hypA_2_B_2_F_2_C_2_D_2_E_2_* (Rmet_1535 through Rmet_1540) all on genomic island CMGI-3. The latter group of *hyp* genes (index 2) appears to have arisen from a reverse duplication of the *hyp* operon of the first *hyp* cluster (index 1) on CMGI-2. We note that the *hyp* and *hox* genes for SH are located, together with the *cbb* genes, on a region flanked by IS elements (Figures S6 and S8A) and that the IS-mediated excission of this region would lead to the loss of RubisCo and the entire SH system, thereby impairing not only autotrophic growth but causing the loss of hydrogenotrophic growth as well. Indeed, stress-induced mutants of CH34 lacking this genomic region are unable to utilize hydrogen gas as a source of energy [6].

Besides these hydrogenase gene clusters on CHR1, there are six *hy*drogenase ‘*f*our’ (*hyf*) genes grouped together on CHR2 as *hyfBCEFGI* (Rmet_4666 through Rmet_4671). This resembles an incomplete operon that, in *E. coli K12*, exists as a 10-gene cluster encoding a multimeric hydrogenase complex. Presumably, this complex interacts with formate dehydrogenase FdhF to form a hydrogenlyase system cleaving formate to dihydrogen and carbon dioxide [185]. The same six *hyf* genes also occur on the first replicon CHR1 of *C. eutrophus* H16 as genes H16_A2196 through H16_A2201 [45], [169]. However, because of the lack of at least four subunit-encoding genes, and the structural differences in the CH34 and H16 Hyf proteins compared to the *E. coli* K12 Hyf units, it is very unlikely that the *hyf* genes in either strain are involved in any aspect of lithotrophic growth.

The *C. metallidurans* MBH is a typical [Ni-Fe]-hydrogenase [186] composed of three subunits (Figure S8B): HoxG, holding the active hydrogen-splitting Ni-Fe centre; HoxK, an electron-transferring subunit containing two 4Fe-4S clusters; and HoxZ, a membrane-bound di-heme cytochrome b. The HoxK signal sequence contains the SRRSFMK sequence recognized by the Twin arginine translocation (Tat) system. Consequently, MBH is exported from the cytoplasm to the periplasmic space, to where its enzymatic activity is limited. The *C. metallidurans* SH is classified as a cytoplasmic bidirectional or hydrogen-evolving [Ni-Fe]-hydrogenase [186], [187] consisting of five subunits HoxHUFIK, with HoxY holding the e^-^ transferring 4Fe-4S cluster. While the reducing powers of MBH reside solely under the form of reduced ubiquinone (ubiquinol), the SH can directly reduce NAD^+^ to NADH (Figure S8B). Because SH acts as a bidirectional enzyme complex, it reverses its activity with increasing NADH to NAD^+^ ratios, effectively acting as a “redox valve”. Accordingly, SH is an important factor in protecting the electron transfer chain from becoming hyper-reductive. The fact that *C. metallidurans* CH34 can utilize H_2_ instantaneously as a reducing power and hence as an alternative source of energy when nutrients are scarce indicates that chemolithoautotrophic growth is an integral part of the organism's lifestyle.

We point out that, although *C. eutrophus* H16 is known as the model ‘knallgas’ bacterium, the MBH of *C. metallidurans* CH34 appears to be far more efficient than its counterpart in *C. eutrophus* H16 since it can oxidise H_2_ gas at concentrations as low as 3% (v/v) and this under atmospheric conditions (i.e., at 20.9% v/v oxygen) [188]. The remarkable features of the *C. metallidurans* MBH (high catalytic activity, H_2_ affinity, and O_2_ tolerance) might be valuable in the emerging field of microbial fuel-cell technology [189], [190]. Further, the oxygen tolerance of MBH may account for the ability of *C. metallidurans* CH34 to grow autotrophically, even in high-oxygen atmospheres [9].

### Organoautotrophy and degradation of aromatics

*C. metallidurans* CH34 can utilize various recalcitrant organics as sole source of carbon and energy, including volatile compounds such as 1-propanol, 2-propanol, 1-butanol, 2-butanol, and acetone, as well as the monoaromatic hydrocarbons benzene, toluene, o-xylene, and phenol. The CH34 genome encodes at least 18 alcohol dehydrogenases (ADHs) and at least 10 aldehyde dehydrogenases (ALDHs). This multitude of fermentation enzymes may seem unusual for an organism that typically follows respiratory metabolism. However, as discussed in earlier separate sections, under limiting oxygen and carbon conditions CH34 will degrade short-chain fatty acids, dicarboxylated compounds, or energy-storage products, such as polyhydroxybutyrate (PHB). This may engender elevated levels of acetyl-CoA while simultaneously, fewer terminal electron acceptors are available, thus giving rise to partially oxidized toxic products including alcohols and aldehydes. Hence, the primary role of the multiple ADHs and ALDHs in CH34 would be cellular detoxification. Further accumulation of acetyl-CoA may initiate the condensation of two acetyl-CoA molecules to acetoacetyl-CoA by the action of an acetyl-CoA acetyltransferase (AAT; EC 2.3.1.9). This compound then may be converted by an acetyl-CoA:acetoacetate CoA transferase (CoAT; EC 2.8.3.8) to acetoacetate, which may have various metabolic fates. However, some bacteria change acetoacetate further into acetone via acetoacetate decarboxylase (ADC; EC 4.1.1.4). The CH34 genome carries the genes for ten AATs very similar in length and sequence (though some may have alternate functions in the cell, for example, the β-oxidation of fatty acids and dicarboxylates, or PHB metabolism) and has two CoATases encoded by two independent *atoAD* loci (Rmet_1153/4 and Rmet_5059/60). However, we detected no ADC in the CH34 sequence data (by BlastP analysis against the ADC sequence of *Clostridium acetobutylicum* ATCC 824, a model organism for bacterial solventogenesis). Hence, it is doubtful that CH34 produces acetone directly from acetoacetate through decarboxylation.

We were very surprised to learn that the CH34 genome encodes an acetone carboxylase (ACX; EC 6.4.1.6) (encoded by Rmet_ 4105/6/7), an (α_2β2_γ_2_) multimeric enzyme involved in acetone metabolism and identified in only very few bacteria [191], [192]. The ACX catalytic reaction basically is the reverse of the ADC reaction in that it carboxylates acetone to generate acetoacetate that is metabolized to acetoacetyl-CoA and further to acetyl-CoA for oxidation in the TCA cycle. The *acxABC* genes in *C. metallidurans* CH34 encoding the three subunits of ACX seemingly are regulated by the AcxR transcriptional activator, whose gene (Rmet_4104) immediately precedes *acxABC* and lays in the opposite orientation, closely resembling the *acxRABC* operon of *Xanthobacter autotrophicus* Py2 [192]. Both in *X. autothropicus* and *C. metallidurans* the AcxR protein contains a SELFGXXXGAFTGA motif that is typical for σ^54^ subunit binding regulators [193], and there is a clearly identifiable σ^54^ promoter sequence TGGCATGNNNNTTGC in the *acxRA* intergenic region. Importantly, proteomic studies demonstrated that the growth of strain CH34 on acetone or 2-propanol as the sole carbon source induces ACX expression (C. Rosier, pers. comm.). A survey of CH34-related genomes using the MaGe interface reveals that the *C. pinatubonensis* JMP134 megaplasmid displays an *acxRABC* operon almost identical to the CH34 *acx* locus in organization and sequence (Ceut_C6170/72/73/74; with aa sequence identities of 89-93% between the respective ACX subunits and 71% identity between the AcxR proteins). None of the other organism listed in Table 2 appears to harbor an *acxRABC* locus. According to current MaGe data, only *Azorhizobium caulinodans* ORS 571 carries an *acxRABC* locus closely resembling the CH34 *acx* locus (with 62-71% sequence identity between the respective ACX subunits and 41% sequence identity between the AcxR proteins) albeit that its *acrR* gene is oriented in the same direction as the *acxABC* genes. We also detected *acx* loci in *Mesorhizobium loti* MAFF 303099 and *Sinerhizobium loti* 1021 with 37-40% sequence identity of their ACX subunits to the respective ACX subunits of CH34. However, the *M. loti* and *S. loti* loci do not code for an AcxR-type σ^54^ dependent transcriptional activator, but for a LuxR/FixJ type transcriptional regulator.

Bacteria can produce acetone through the dehydrogenation of 2-propanol (iso-propanol) or via the oxidation of 2-nitropropane by a 2-nitropropane dioxygenase (NPD; EC 1.3.11.32). Among the 18 ADH genes we detected in CH34, the most likely candidate for an isopropanol dehydrogenase (iPDH) is Rmet_5645 because its product is highly similar in length and sequence to the multifunctional ADH of *C. eutrophus* H16 (H16_A0757) [194]. In addition, the CH34 genome contains six NPD encoding genes (Rmet_0222, Rmet_2083, Rmet_2605, Rmet_3674, Rmet_5434, and Rmet_5534). The production of acetone by CH34 would explain the presence of an ACX because this way cells can utilize it for growth and keep (toxic) acetone levels low.

Benzene, toluene (methylbenzene), ethylbenzene, and xylene (dimethylbenzene) jointly are known as BTEX compounds, with phenol as a major chemical intermediate. In *C. metallidurans* CH34 aerobic degradation of BTEX involves ring hydroxylation by one of three different multicomponent monooxygenases and proceeds via typical *meta*-cleavage pathways initiated via catechol decyclation by a 2,3-dioxygenase (in contrast to 1,2-dioxygenase *ortho*-cleavage pathways that cleave the catechol ring between the two hydroxyl groups). Bacterial multicomponent monooxygenases (BMMs), transcribed from single operons encoding four to six subunits, are classified in six distinct groups [195]. Although the genomes of aromatic-degrading strains only rarely carry more than one BMM [195], Cafaro *et al.* [196] suggested that multiple BMMs in the same system may be beneficiary in terms of BMM specificity and efficiency of substrate utilization. We identified two Group 1 (phenol hydroxylases; PHs) and one Group 2 (toluene-benzene monoxygenases; TOMOs) BMMs in two different loci of the CH34 genome. The first locus (Rmet_1305 to Rmet_1331) is part of the CMGI-2 genomic island and contains the genes for two BMMs: a phenol hydroxylase, encoded by six genes Rmet_1326 to Rmet_1331, and a benzene-toluene monooxygenase, encoded by six genes Rmet_1311 to Rmet_1316. All genes are present for the *meta*-cleavage of catechol (by an 2,3-dioxygenase encoded by Rmet_1324), and its subsequent enzymatic processing (by the products of genes Rmet_1317 to Rmet_1322) to the final product acetyl-CoA. The Rmet_1305 gene (*patR*) was identified as a σ^54^-dependent Fis-type transcriptional regulator. This entire region is in high synteny with the aromatic degradation gene clusters in the related genomes of *C. pinatubonensis* JMP134 (Reut_B5671 through Reut_B5693) and *Ralstonia pickettii* 12D (Rpic_5178 through Rpic_5201). The second locus (Rmet_1781 to Rmet_1788) encodes a second phenol hydroxylase (PH) also consisting of six subunits (Rmet_1782/3/4/5/6/7), with Rmet_1788 encoding the Fis-type regulator PoxR. However, gene Rmet_1781 encodes a 1,2-dioxygenase with high similarity (64% aa sequence identity) to the 1,2-dioxygenase for ortho-cleavage of benzoate (encoded by Rmet_4881; see ‘Heterotrophic carbon metabolism’ in this discussion). This eight-gene cassette, including a 1,2-dioxygenase gene, also occurs in strain JMP134 (as Reut_A1698 to Reut_A1705) and in *Wautersia numadzuensis* strain TE26 (as the *phtRABCDEF-catA* locus; [197]). The very high sequence similarities between the eight corresponding gene products of the CH34, JMP134, and TE26 loci (82–99% sequence identity) point to their acquisition via gene transfer as one cassette.

The combination of a phenol hydroxylase gene cluster with a 1,2-dioxygenase gene seems to be very rare and confined to the above-mentioned strains. As far as we are aware, *P. putida* strain GB1 is the only other organism with a similar locus (as evidenced by MaGe data) although the 1,2-dioxygenase gene is not located downstream of the hydroxylase genes, but on the other side of the regulatory gene. Also, the sequence similarity between the corresponding hydroxylase subunits is much lower (48–68% sequence identity). Notomista *et al.* [195] postulated that the modularity of multiple BMMs in the same system may foster the invention of new mixed pathways, or even hybrid BMMs with novel substrate specificities. Here, it might be valuable to explore systematically the aromatic-substrate specificity of *C. metallidurans* CH34, as was done recently for *C. pinatubonensis* JMP134 [198]. Only then can we correlate aromatics degradation with pathway predictions and determine the true catabolic potential of CH34.

### Polyester synthesis PHA/PHB

Bacteria produce polyhydroxybutyrate (PHB), a polyester polymer belonging to the family of polyhydroxyalkanoates (PHAs), as a carbon- and energy-reserve to be metabolized after exhausting all other carbon resources (reviewed by Lenz and Marchessault [199]). The bacterial synthesis of PHAs, such as PHB, has gathered renewed interest because their physical properties can be remarkably similar to those of the plastic polypropylene, and because, unlike polypropylene, it is rapidly biodegradable (hence the name “bioplastic” or “green plastic”). Although there are various bacterial pathways to create PHAs [200], the key model one presently used is that from *C. eutrophus* H16 which encompasses only three enzymes: A PHA synthase (or polymerase) encoded by *phaC*; an acetoacetyl-CoA reductase encoded by *phaB*; and, a β-ketothiolase encoded by *phaA*. The synthesis of PHB has been studied most extensively in *C. eutrophus* H16 because it can generate PHB solely using simple carbon substances, such as gluconate, via the Entner-Doudoroff (ED) pathway [45]. Additional genes playing a role in PHB synthesis are *phaR* encoding a transcriptional regulator, and *phaP* encoding a phasin acting on the surface of PHA granules. Phasins inhibit the coalescence of the granules to regulate their number, and may take part in mobilizing the storage polymer. Lastly, several *phaZ* paralogs encode the intracellular depolymerases needed for PHB homeostasis and degradation.

Like its relative *C. eutrophus* H16, *C. metallidurans* CH34 synthesizes PHB and probably accumulates it in the form of intracellular storage granules. Interestingly, PHB synthesis in CH34 is initially (i.e., after 24 hours of growth) enhanced under simulated microgravity [41]. The CH34 genome has three copies of *phaC*, one copy each of *phaA* and *phaP*, three copies of *phaB*, and one regulatory gene *phaR* (for Rmet numbers see Table S9). These PHB synthesis genes mainly are organized into two casettes: *phaC1-phaA-phaB1-phaR* (Rmet_1356-1359) located on the CHR1, and *phaC3-phaB3* (Rmet_5122-5123) located on CHR2. All the other genes are scattered throughout the genome (Table S9). We also found isologs of *phaA* (*bktB, atoB*) encoding additional acetoacetyl-CoA thiolases but these were annotated as having a different substrate specificity than PhaA (Table S9). For intracellular PHB depolymerization, we identified three *phaZ* genes on the *C. metallidurans* CH34 genome: One on CHR1 and two on CHR2. These phaZ genes correspond with the phaZ1, phaZ2 and phaZ3 genes of *C. eutrophus* H16 identified by York *et al.* [201]. In addition, the CHR1 replicon carries a gene for a PHB-oligomer hydrolase (designated by *phaY* or Rmet_1960) that is an ortholog of the earlier identified 3-hydroxybutyrate-oligomer hydrolase (3HBH) of *C. eutrophus* H16 [202]-[204]. The 3HBH enzyme in *C. euthrophus* H16 degrades the trimeric products formed by intracellular PHB depolymerases [205]; based on the very high aa similarity (63%), PhaY seems to have the same function in *C. metallidurans*. Lastly, a gene fragment (Rmet_1670) located adjacent to the *phaC2* gene (Rmet_1671) is predicted by the MaGe system to encode a defective γ-aminobutyrate transporter, indicating that these genes might be remnants of an obsolete PHB biosynthesis operon.

### DNA repair genes and stress

The robustness of *C. metallidurans* CH34 under a wide variety of harsh environmental conditions does not rest just on its efficient transport and detoxification of harmful substances, ions or molecules, but also greatly depends on safeguarding its genetic material from irreversible damage. Generally, genome integrity is maintained by a number of DNA-repair mechanisms, including excision repair, direct repair, and recombinational repair. The CH34 genome contains DNA-repair genes for base-excision repair (*alkA, mutY, tag, ung, fpg, nth, xthA*) (all Rmet numbers are given in Table S10) and the complete pathway for nucleotide-excision repair (*mfd, uvrA, uvrB, uvrC, uvrD*). Direct DNA repair in strain CH34 is mediated by photoreactivation (*phr*), oxidative demethylation (*alkB1*, *alkB2*), and alkyltransferases activity (*ada*, *ogt1, ogt2, ogt3*). Its genome does not seem to carry a RecBCD nor a RecFOR pathway to repair single-strand gaps and double-strand DNA breaks by homologous recombination. The lack of these genes was previously postulated to allow the transformation in CH34 of large linear ds DNA fragments [206]. Nonetheless, strain CH34 has a functionally analogous system in the form of AddAB that is prevalent among β- and α-proteobactaria and firmicutes [207]. The CH34 genome carries the genes encoding the Holliday-junction protein complex RuvAB, the resolvase RuvC, a dsDNA exonuclease SbcD, and the mismatch-repair proteins MutS and MutL. These genes *ruvAB*, *ruvC*, *sbcD*, and *mutSL* often co-occur within genomes [207]. Interestingly, CH34 does not contain a *mutH* gene encoding a dGATC endonuclease but a *dam* homologous gene is present (Rmet_5483, located on CHR2). Dam affects the transposition frequency of several transposons and IS elements (Tn*10*, Tn*5*, Tn*903*, IS*3*, and possibly others), and modulates gene expression by the adenine methylation of DNA regulatory sites [208]. The presence of Dam in *C. metallidurans* CH34 might well be significant in view of the complex transcriptional networks and the large number of mobile genetic elements intrinsic to its flexible, fluid genome.

In addition, CH34 harbors the main genes of the RpoH regulon (heat shock) and stringent response (*relA* and *spoT*), the main regulatory genes of the SOS reponse (*lexA* and *recA*), and the oxidative-stress-response regulators, OxyR and SoxR (all Rmet numbers in Table S10). *C. metallidurans* CH34, however, lacks a SoxS homolog and does not carry any typical SoxR binding site, as indicated by a genome-scale pattern search using the SoxR binding site consensus sequence [209] and the Regulatory Sequence Analysis Tools webservice (RSAT) [210] (results not shown). Therefore, the involvement of SoxR-mediated regulation of the oxidative-stress response is unresolved. Finally, CH34 harbors 15 proteins belonging to the universal stress protein (UspA) superfamily (Table S11). Generally, UspA proteins are induced in response to a variety of starvation and stress conditions and have partially overlapping but distinct biological functions [211]. Eleven genes encode small proteins harboring one Usp domain, two encode intermediate proteins with one Usp domain (*uspA13, uspA14*), and two encode large proteins consisting of two Usp domains (*uspA6, uspA11*). The *uspA11* gene is present in 2 copies and located on transposon Tn*6050*. All but five (*uspA3, uspA4, uspA13, uspA14, uspA15*) contain the G-2X-G-9X-[S/T] motif that is shared by many members of the UspA family [212].

### Conclusion

We have embarked on an exciting journey through the genome of a versatile soil bacterium, *C. metallidurans* strain CH34, best known for its tolerance to a wide range of heavy metals and representative for a species that is mainly found in areas linked to mining sites and metallurgical or chemical industries. Genome data show that this genome underwent numerous genetic adaptations in its evolutionary history by continuously gathering novel genes and pathways, and by rearranging and combining them in new clusters useful for its survival. This continuous acquisition of genes, and clusters of genes, has led to the co-occurrence of many complementary systems either involved in heavy-metal resistance or other types of stress, requiring intricate fine-tuning of cellular responses on many different levels. Accordingly, its complex genome boasts a vast network of regulatory proteins, including 18 sigma factors and hundreds of transport, signal sensory, and transduction systems. Although we only have just begun to understand this complexity, genome data presented here will now allow us to systematically link whole genome expression studies with high quality annotation data that are publicly available via the MaGe system. In addition, the comparison of the *C. metallidurans* CH34 genome with the genomes of three other *Cupriavidus* strains, each from a species with a particular lifestyle and habitat, will offer many clues on their physiology, metabolic capacities, and distinct interactions with their environment.

## Materials and Methods

### *C. metallidurans* strain CH34 isolation and propagation

Strain CH34 of *C. metallidurans* was isolated previously as described [3]. This strain is the type strain of the species (currently holding at least 17 strains [32]), and was deposited into the BCCM/LMG public strain collection (http://bccm.belspo.be/) as strain LMG1195. The bacterium was routinely grown at 28–30°C in rich Luria-Bertani medium or in Tris-salt mineral medium with gluconate as sole carbon source (MM284). The latter medium especially was used for its growth in the presence of heavy metals [9].

### Sequencing, assembly, and gap closure

The complete genome sequence of *C. metallidurans* strain CH34 was determined using the whole-genome shotgun method at the Joint Genome Institute (JGI), USA, using a combination of three randomly sheared libraries that contain inserts in the 3kb and 8kb (plasmids), and 40 kb (fosmids) size range. All general aspects of library construction and sequencing performed at the JGI are described at http://www.jgi.doe.gov/. These libraries were sequenced to a total depth of 9.9X, and all reads assessed for quality. Before assembling the reads, all were trimmed by removing vector-specific sequences. Libraries were assembled using the Paracel Genome Assembler (PGA; http://www.paracel.com/). Possible faulty assemblies, resulting from large repeat elements such as IS elements, were corrected by verifying the correspondence between the left- and right-direct repeat (i.e., of the IS element) via PCR amplification and sequence analysis of the predicted product. Gaps between contigs were closed by using the Consed editing software [213], custom primer walks, and/or PCR amplification. The final assembly consisted of 184,165 Sanger reads (i.e., using conventional dye-terminator chemistry). Of those, 3,752 were finishing reads used to correct errors in assembling the draft. The estimated error rate of the completed genome sequence was 1 in 2.5 Mb, or less. The final assembly contained two chromosomes (3,928,089 bp and 2,580,084 bp in size) and two plasmids (233,720 bp and 171,459 bp in size). We verified the consistency of the chromosome whole assembly by comparing the genome sequence map with a genetic experimental map obtained by conjugation [22]. The sequence and annotation data of the four replicons of *C. metallidurans* CH34 were deposited in the NCBI database (http://www.ncbi.nlm.nih.gov/Genbank/) under the GenBank accession numbers NC_007971 (for pMOL30), NC_007972 (for pMOL28), NC_007973 (for CHR1), and NC_007974 (for CHR2).

### Structural and functional genome annotation

Prediction of ORFs and other genome features (RNA encoding genes, riboswitches, ribosome binding sites, signal sequences, etc.) as well as functional predictions were done by the MaGe platform [48]. This system allowed the manual curation of all genes and their functions. MaGe is also interlinked to metabolic pathway tools such as KEGG and BioCyc, displays synteny maps for easy genome comparison, and has an exploration interface to query the underlying SQL databases. All *C. metallidurans* CH34 project data are freely available at https://www.genoscope.cns.fr/agc/mage/.

### Circular plots

Circular maps for the two main replicons CHR1 and CHR2 (Figure 1) were drawn using the Circos software [214]. We calculated the GC content and GC-skew data employing the *gc2viz.pl* and *gcskew2viz.pl* scripts from the GenomeViz package [215].

### Identification of *ori* and *ter* regions

Regions representing the putative origin of replication (*ori*) and termination (*ter*) were predicted by Ori-Finder software [216] that combines gene finding, detection of base composition assymetry (e.g. GC-skew), and analysis of DnaA-box distribution. These regions were scrutinised for additional features and repetitive elements (Figure S1).

### Distribution of functional classes

Functional classes were classified in COGs (Clusters of Orthologous Groups) by the COGnitor software imbedded into the MaGe system. All COG tables were retrieved from MaGe via the Export pages and the derived CDS numbers were used to calculate the normalised distribution of functional content over the two main replicons (Table S1; Figure 2).

### Clustering

Hierarchical clustering was performed via the *heatmap.2* function of the *gplots* library in R (version 2.8.0) using single linkage clustering. For clustering of the different replicons (Figure 3; Figure S3), we normalized the obtained numbers of orthologs shared between two replicons according to the actual size of the replicon, i.e., the distance scores used in hierarchical clustering were calculated as [1 – number of orthologs/size of the smallest genome] [217]. For the clustering of different RpoD proteins (Figure S5) we used the distance measure [100 – the percentage of protein similarity].

### Genome comparative analysis

Whole genome alignments of the four *Cupriavidus* and the *R. solanacearum* genomes (Figure 4; Figure S2) were performed using the anchor-alignment software Murasaki (http://murasaki.dna.bio.keio.ac.jp/) [218]. Genome comparisons between *C. metallidurans* CH34 and *P. necessarius* STIR were done using either the LinePlot tool of MaGe [48] or the PROmer module of the MUMmer v3.0 package [219].

### Clustering of protein families (paralogy)

To classify protein sequence sets into families according to sequence similarity we used the GeneRAGE algorithm [56]. This algorithm constructs a binary matrix holding all similarity relationships from an all-to-all protein sequence comparison performed with BLAST v2.0 [220] and deploys the CAST algorithm [221] to filter sequences for low-complexity regions. The matrix then is processed for symmetry and transitivity relationships using successive rounds of the Smith-Waterman dynamic programming algorithm [222]. In this way, false relationships within the matrix are detected, as well as multi-domain protein families are detected. We have used a cut-off *E*-value threshold of 10^−10^ for all BLAST comparisons, and the default Z-score values for symmetrification and multi-domain detection (10 and 3, respectively).

### Identification of orthologs

Protein sequence similarity was determined using BLAST (v2.0) software [220]. Proteins between different organisms were defined as orthologs when (i) they displayed a minimal percentage sequence identity of 35%; (ii) they are each other's reciprocal best BLAST hit; and, (iii) the sequence identity was measured over at least 80% of the smallest protein.

## Supporting Information

Figure S1 Origins of replication for the main replicons (chromosomes) CHR1 and CHR2 of *C. metallidurans* CH34 (panels A and B, respectively). (A) DnaA-binding boxes with consensus sequence TTATCCACA are given as blocked arrows. Dark shaded boxes have a perfect match with the consensus sequence, while lighter shaded boxes have one or two mismatches (marked in red in their respective sequences). (B) Putative RepA-binding sites (see text) are indicated as ovals. Blue shaded ovals indicate putative RepA-binding sites on the opposite strand.
(0.05 MB PDF)

Figure S2 Genome dynamics in the second chromosome of *Cupriavidus* and *Ralstonia*. Nucleotide based comparison of the CHR2 replicons of four *Cupriavidus* species and *R. solanacearum* GMI1000 (denoted as Rsol) using the anchor-allignment software Murasaki (http://murasaki.dna.bio.keio.ac.jp/). Scale in Mb is shown on top. Abbreviations: Cmet, *C. metallidurans* CH34; Cpin, *C. pinatubonensis* JMP134; Ceut, *C. eutrophus*; Ctai, *C. taiwanensis*.
(1.86 MB TIF)

Figure S3 Similarity matrix between CH34 replicons and replicons of related organisms using protein based orthology data. Protein contents of all replicons for four *Cupriavidus* species and related organisms were compared by scoring the reciprocal best BlastP hits (values in lower part of the matrix) and subsequent cluster analysis of normalised values (upper part of the matrix) (see Methods). Most analysed replicons broadly fall into two large clusters A and B, with main chromosomes grouping tightly together, while three plasmids group together in cluster C owing to a backbone of essential genes (see also text). General information on all those species is given in Table 2. The genome of *Escherichia coli* K-12 served as a reference and outlier. Abbreviations: MPL, megaplasmid; Cmet, *C. metallidurans* CH34; Cpin, *C. pinatubonensis* JMP134; Ceut, *C. eutrophus* H16; Ctai, *C. taiwanensis*; Ecol, *Escherichia coli* K-12; Bxen, *Burkholderia xenovorans* LB400; Bpet, *Bordetella petrii*; Jmar, *Janthinobacterium* sp. Marseille; Hars, *Herminiimonas arsenicoxydans*; Daci, *Delftia acidovorans* SPH-1; Rsol, *Ralstonia solanacearum* GMI1000; and Pnec, *Polynucleobacter necessarius* STIR1. Replicons are denoted by their commonly known name (e.g., pRALTA) or subindexed (e.g., Cmet_CHR1).
(2.72 MB TIF)

Figure S4 Protein-based synteny between the *C. metallidurans* CH34 chromosomes and the genome of *P. necessarius* STIR1, an obligate symbiont of freshwater protozoans. (A) Synteny displays were generated by the LinePlot tool of the MaGe system using a synton size (S) of 3 genes. Both species belong to the family of *Burkholderiaceae* and a plausible reason for the high synteny between CH34-CHR1 and the STIR1 genome would be the preservation of essential genes during genomic reduction in the STIR1 symbiont. Genes indicated on the positive (+) and negative (−) strand are rRNA (blue), tRNA (green), and transposons (pink). Strand conservations and strand inversions between de compared replicons are colored green and red, respectively. (B) Dot plot analysis of MUMs (maximal unique matching subsequences) between the main chromosome of *C. metallidurans* CH34 and the *P. necessarius* STIR1 genome. MUMs between both forward strands are shown in red and MUMs between the forward and reverse strands are shown in blue. The analysis was performed at protein level (using the six reading frames) deploying the PROmer module of MUMmer software with default parameters.
(0.18 MB PDF)

Figure S5 Sigma-70 sequence similarity heatmap based on pair-wise Smith-Waterman alignments of RpoD sequences. While the RpoD1 protein of CH34 (Rmet_2606 sited on CHR1) groups tightly with RpoD orthologs from the other three *Cupriavidus* species, the second sigma-70 factor RpoD2 (Rmet_4661 sited on CHR2) does not group with any of the RpoD orthologues in *Cupriavidus* but displays more significant BlastP hits with the RpoD proteins of *Bordetella* and *Burkholderia* species. All gene notations are from GenBank. Abbreviations: Rmet, *C. metallidurans* CH34; Bxe, *B. xenovorans* LB400; Reut, *C. pinatubonensis* JMP134; RALTA, *C. taiwanensis*; H16, *C. eutrophus* H16; Bpet, *B. petrii*; Daci, *D. acidovorans* SPH-1; Pnec, *P. necessarius* STIR1; mma, *Janthinobacterium* sp. Marseille; HEAR, *H. arsenicoxydans*; RSc, *R. solanacearum* GMI1000; b3067 denotes the RpoD protein sequence of *E. coli* str. K-12 substr. MG1655.
(0.73 MB PDF)

Figure S6 Schematic view of the *cbb* loci in *C. metallidurans* CH34. Loci involved in the Calvin-Benson-Bassham cycle (reductive pentose phosphate pathway) are dispersed over the CH34 genome although most are within the Rmet_1491 through Rmet_1544 region. Note that IS-mediated excision of this region (or parts thereoff) would lead to the impairment of autotrophic growth in CH34, a phenomenon that can be observed when strain CH34 is under mutagenic stress (see text).
(0.07 MB PDF)

Figure S7 Genes and pathways for sulfur oxidation (sulfur autotrophy) in *C. metallidurans* CH34. (A) Genetic loci encompassing sulfur oxidizing genes (*sox*) and sulfur oxygenase reductase genes (*sor*). Note that strain CH34 lacks the *soxCD* genes for a periplasmic sulfur dehydrogenase which is not essential for the oxidation of thiosulfate although fewer electrons are generated (see text and next panel). (B) Proposed pathway for the oxidation of thiosulfate (S_2_O_3_^2−^) to sulfate (SO_4_^−^) by the combined action of the sulfur carrier protein SoxYZ, a cytochrome c-type complex SoxXA, and the sulfate thiol hydrolase SoxB. (C) Proposed pathway for the oxidation of sulfide (HS^−^) by the SoxEF c-type flavocytochrome complex in which SoxF acts as a sulfide dehydrogenase. (D) Proposed pathway for the oxidation of sulfite (SO_3_^−^) to sulfate (SO_4_^−^) through the action of a periplasmic sulfite dehydrogenase, SorA, and a membrane-bound c-type cytochrome, SorB.
(0.05 MB XLS)

Figure S8 Genetic loci in *C. metallidurans* CH34 for the oxidation of hydrogen. (A) *hox* and *hyp* genes needed to form and activate the two hydrogenases of CH34. The first cluster of genes governs the membrane-bound hydrogenase (MBH) and is sited on CHR1 as part of the CMGI-2 genomic island. The second gene cluster encodes the cytoplasmic hydrogenase (SH) and is sited on CHR2 as part of the genomic island CMGI-3. (B) Multimeric composition of the MBH and SH metalloproteins and their cellular location in *C. metallidurans* CH34. While the MBH enzyme is transloacted to the periplasmic space and transfers electrons in one direction, the SH remains inside the cytoplasm and is able to transfer electrons in both directions, depending on NADH to NAD^+^ ratios in the cell. As such, the SH enzyme effectively acts as a ‘redox valve’.
(0.06 MB XLS)

Table S1 Functional classification. Normalised distribution of functional content over the two large replicons of *C. metallidurans* CH34, based on COG assignments. These data were used for colorcoding in Figure 1 and the drawing of Figure 2.
(0.08 MB PDF)

Table S2 Novel HMR genes in *Cupriavidus metallidurans* CH34. Correspondance between HMR loci reported here with HMR loci reported in an earlier overview [8]. Novel genes are given in bold letter type.
(0.02 MB XLS)

Table S3 Efflux and transport proteins in *C. metallidurans* CH34. Transport proteins predicted by TransAAP analysis for the four *Cupriavidus* species by submitting the genomes of these species to the TransportDB website (http://www.membranetransport.org/). Only five classes were considered (indicated with blue bars). Transporters that are not present in *C. metallidurans* CH34 but are present in one or more of the other *Cupriavidus* strains are shaded in gray. Reversely, transporters that are present in CH34 but are not in either of the three other strains are shaded in green.
(0.03 MB XLS)

Table S4 Sigma factors of *C. metallidurans* CH34. The 17 sigma factors of *C. metallidurans* CH34 and their orthologs in related organisms (as listed in Table 2) and *E. coli* K-12. Note that some organisms, including strain CH34, possess multiple sigma-70 factors (RpoD proteins). All gene notations are from GenBank (and are as in Figure S5).
(0.02 MB XLS)

Table S5 Transcription factors - meta analysis. Transcription factors across 965 organisms, including 673 bacteria (source: http://www.transcriptionfactor.org/). Note that this table consist of multiple sheets: Sheet 1, DBDoverview - general information and data from DBD; Sheet 2, DBD organism - breakdown of transcriptional regulators for each of the organisms listed in Table 2 and *E. coli* K-12; Sheet 3, MageReplicon - distribution of transcriptional regulators across the four replicons of CH34, using MaGe data.
(0.62 MB XLS)

Table S6 Functional distribution and chromosomal location of *C. metallidurans* CH34 genes implicated in iron uptake and -metabolism.
(0.12 MB DOC)

Table S7 Signal transduction systems. Domain profiles of *C. metallidurans* CH34 and related organisms listed in Table 2 and *E. coli* K-12 (source: http://mistdb.com/). Only domains that occur 10 times or more in CH34 are considered. Extreme underrepresentations of domains (according to actual numbers versus genome size) are shaded in orange. Rows 25–27 hold the distributions of predicted one-component and two-component signal transduction systems (denoted as 1CST and 2CST, respectively) as well as the total number of predicted systems. The latter was used to calculate the proportional number (in percentages) of proteins putatively involved in signal transduction.
(0.02 MB XLS)

Table S8 Prevalence of Bug domains according to the MiST2 database. Distribution of 2,377 proteins with Bug domains across 1,216 complete and draft, bacterial and archaeal genomes (source: http://mistdb.com). Organisms without Bug domain-containing proteins are omitted from the table.
(0.07 MB XLS)

Table S9 Functional distribution and chromosomal location of *C. metallidurans* CH34 genes involved in polyester (PHA/PHB) biosynthesis and degradation.
(0.06 MB DOC)

Table S10 *C. metallidurans* CH34 genes for DNA repair and stress-related regulators.
(0.08 MB DOC)

Table S11 *C. metallidurans* CH34 genes encoding universal stress proteins (UspA superfamily) that are induced by a variety of starvation- and stress-conditions.
(0.04 MB DOC)
