# Supplementary figures and images for: The Complete Genome Sequence of Cupriavidus metallidurans Strain CH34, a Master Survivalist in Harsh and Anthropogenic Environments
Source: PLoS One. 2010 May 5;5(5):e10433. doi: 10.1371/journal.pone.0010433 (PMC2864759; doi:10.1371/journal.pone.0010433)

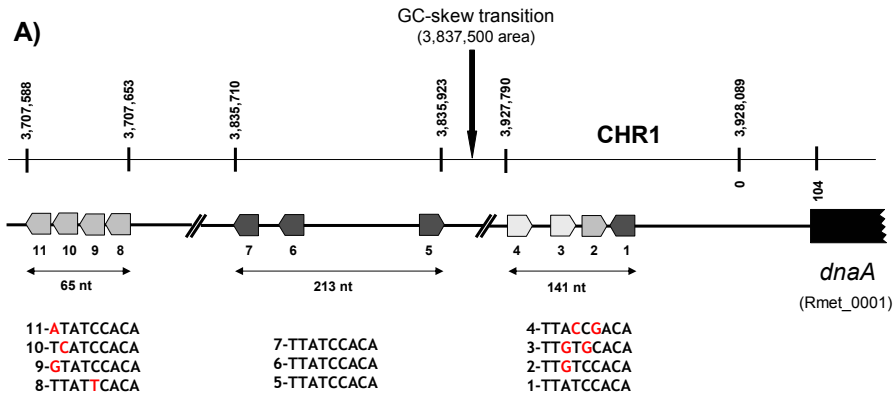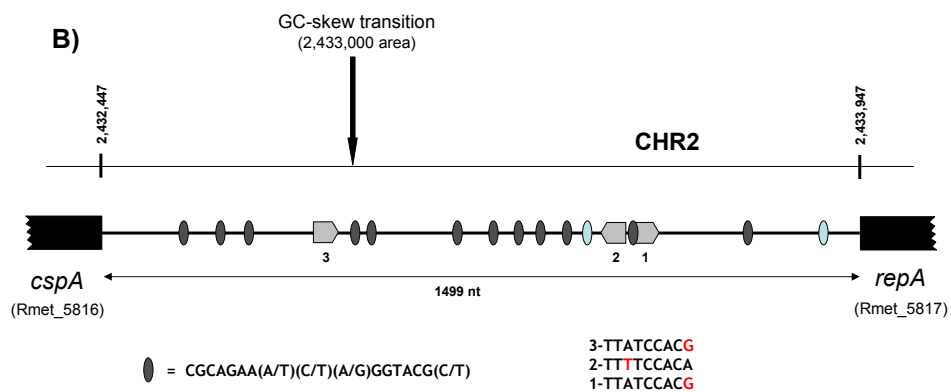

Supplement: Figure S1 — Origins of replication for the main replicons (chromosomes) CHR1 and CHR2 of C. metallidurans CH34 (panels A and B, respectively). (A) DnaA-binding boxes with consensus sequence TTATCCACA are given as blocked arrows. Dark shaded boxes have a perfect match with the consensus sequence, while lighter shaded boxes have one or two mismatches (marked in red in their respective sequences). (B) Putative RepA-binding sites (see text) are indicated as ovals. Blue shaded ovals indicate putative RepA-binding sites on the opposite strand. (0.05 MB PDF) [file pone.0010433.s001.pdf]

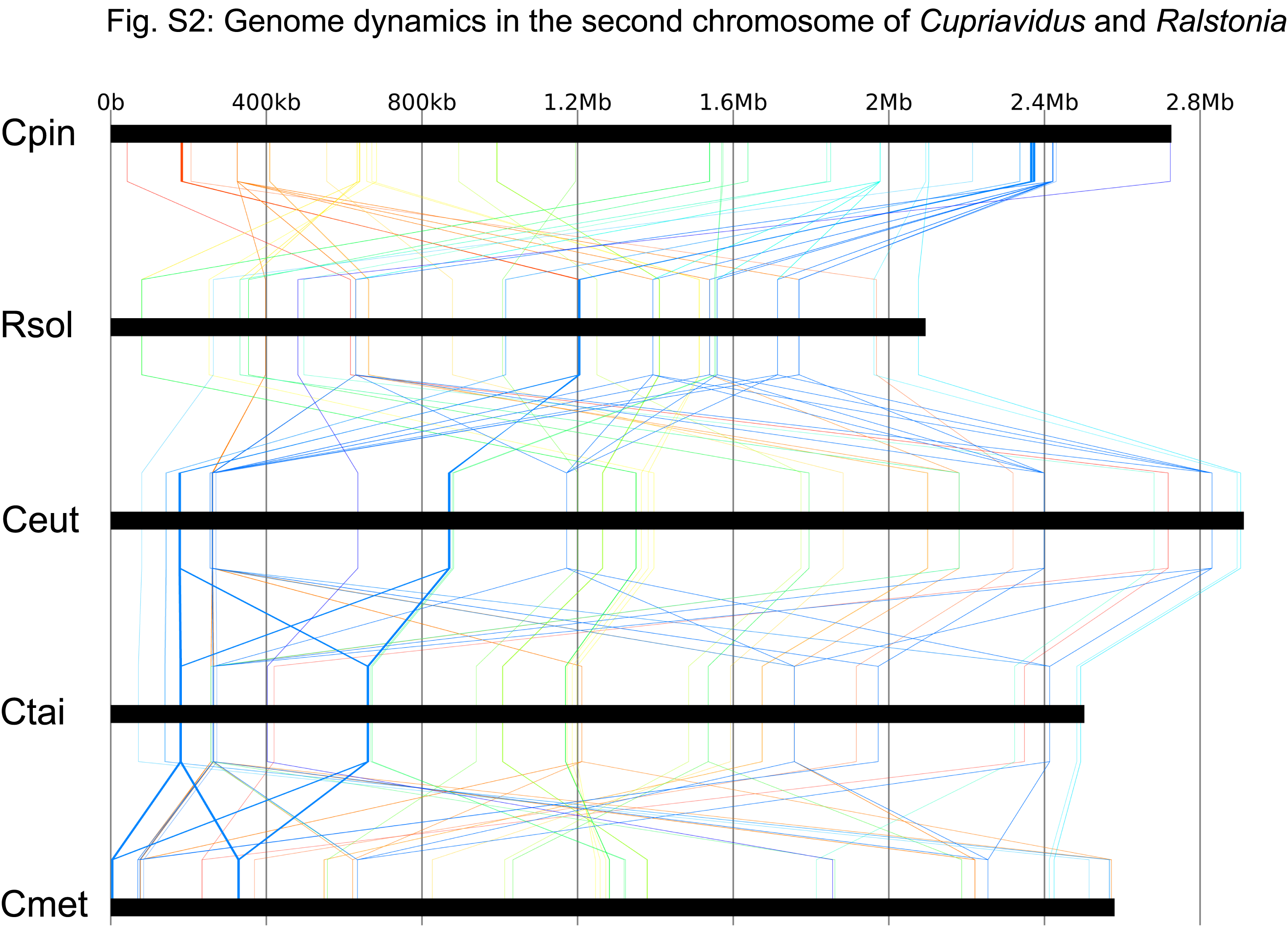

Supplement: Figure S2 — Genome dynamics in the second chromosome of Cupriavidus and Ralstonia. Nucleotide based comparison of the CHR2 replicons of four Cupriavidus species and R. solanacearum GMI1000 (denoted as Rsol) using the anchor-allignment software Murasaki (http://murasaki.dna.bio.keio.ac.jp/). Scale in Mb is shown on top. Abbreviations: Cmet, C. metallidurans CH34; Cpin, C. pinatubonensis JMP134; Ceut, C. eutrophus; Ctai, C. taiwanensis. (1.86 MB TIF) [file pone.0010433.s002.tif]

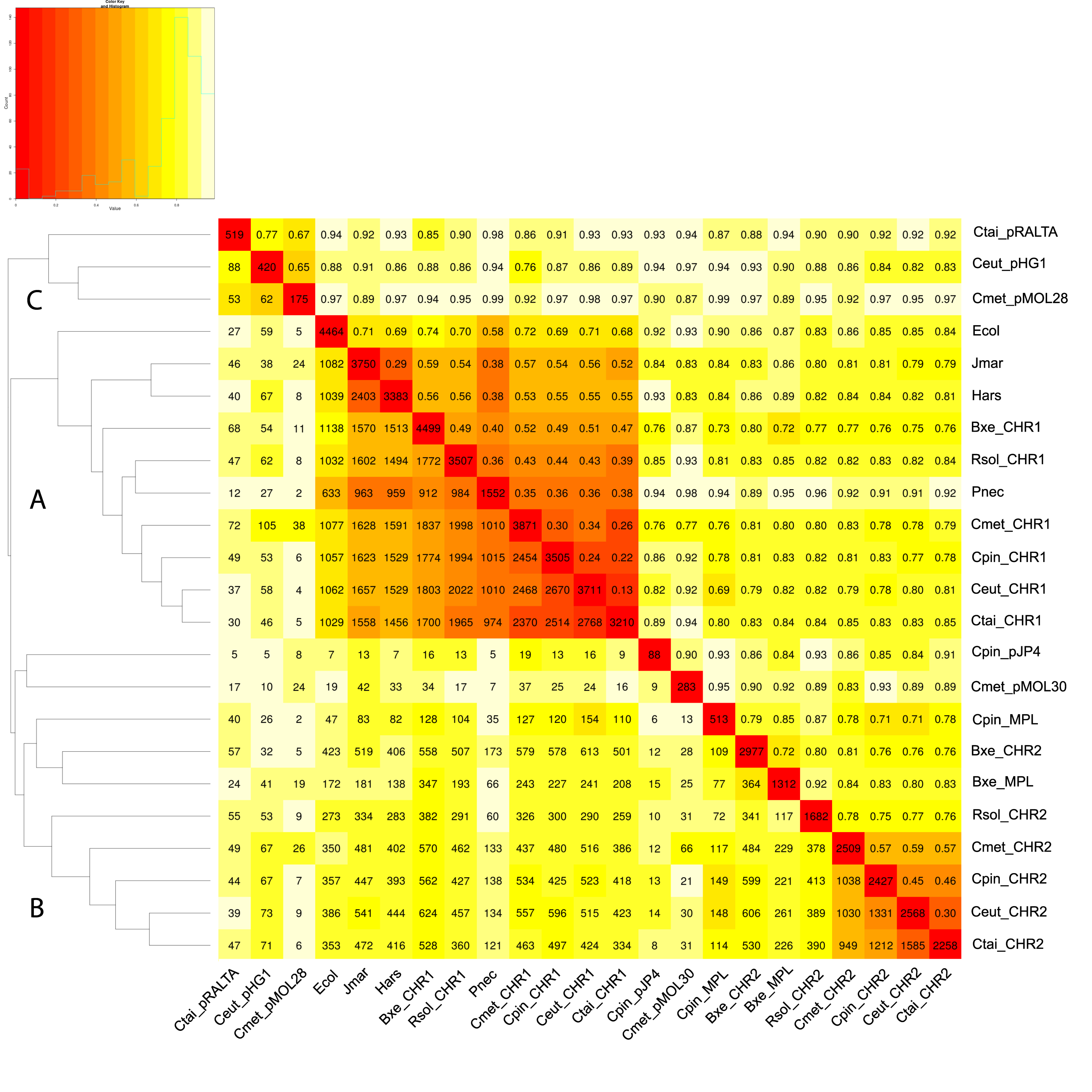

Supplement: Figure S3 — Similarity matrix between CH34 replicons and replicons of related organisms using protein based orthology data. Protein contents of all replicons for four Cupriavidus species and related organisms were compared by scoring the reciprocal best BlastP hits (values in lower part of the matrix) and subsequent cluster analysis of normalised values (upper part of the matrix) (see Methods). Most analysed replicons broadly fall into two large clusters A and B, with main chromosomes grouping tightly together, while three plasmids group together in cluster C owing to a backbone of essential genes (see also text). General information on all those species is given in Table 2. The genome of Escherichia coli K-12 served as a reference and outlier. Abbreviations: MPL, megaplasmid; Cmet, C. metallidurans CH34; Cpin, C. pinatubonensis JMP134; Ceut, C. eutrophus H16; Ctai, C. taiwanensis; Ecol, Escherichia coli K-12; Bxen, Burkholderia xenovorans LB400; Bpet, Bordetella petrii; Jmar, Janthinobacterium sp. Marseille; Hars, Herminiimonas arsenicoxydans; Daci, Delftia acidovorans SPH-1; Rsol, Ralstonia solanacearum GMI1000; and Pnec, Polynucleobacter necessarius STIR1. Replicons are denoted by their commonly known name (e.g., pRALTA) or subindexed (e.g., Cmet_CHR1). (2.72 MB TIF) [file pone.0010433.s003.tif]

## Loci on Chromosome 1

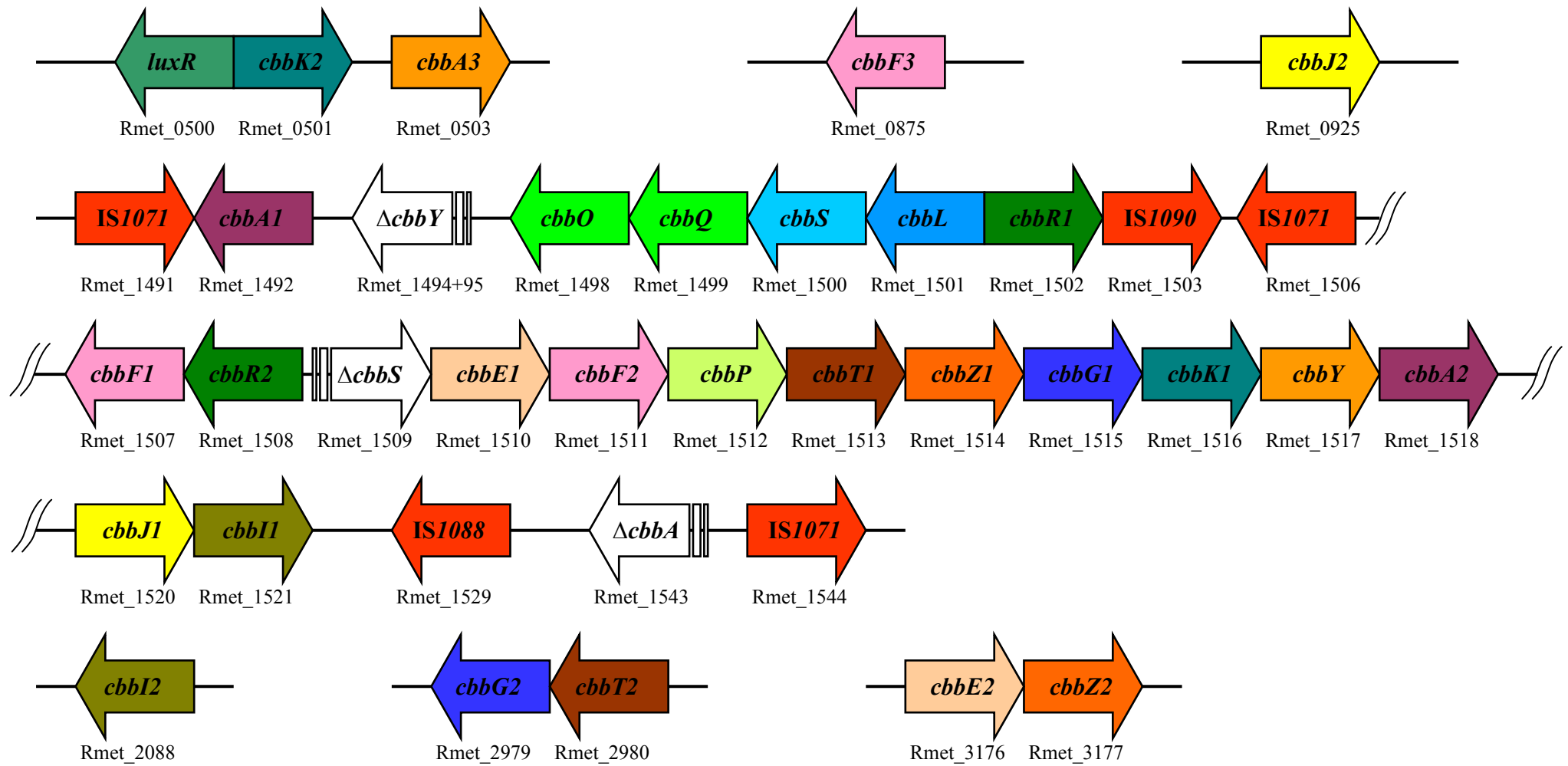

## Loci on Chromosome 2

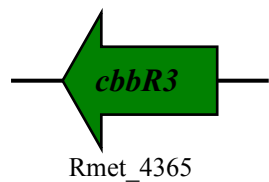

Supplement: Figure S6 — Schematic view of the cbb loci in C. metallidurans CH34. Loci involved in the Calvin-Benson-Bassham cycle (reductive pentose phosphate pathway) are dispersed over the CH34 genome although most are within the Rmet_1491 through Rmet_1544 region. Note that IS-mediated excision of this region (or parts thereoff) would lead to the impairment of autotrophic growth in CH34, a phenomenon that can be observed when strain CH34 is under mutagenic stress (see text). (0.07 MB PDF) [file pone.0010433.s006.pdf]
