# Supplementary material for: The Complete Genome Sequence of Cupriavidus metallidurans Strain CH34, a Master Survivalist in Harsh and Anthropogenic Environments
Source: PLoS One. 2010 May 5;5(5):e10433. doi: 10.1371/journal.pone.0010433 (PMC2864759; doi:10.1371/journal.pone.0010433)

<u>Fig. S4A:</u> synteny between *C. metallidurans* CH34 replicons and *P. necessarius* STIR1 (analysis performed by the LinePlot tool of MaGe)

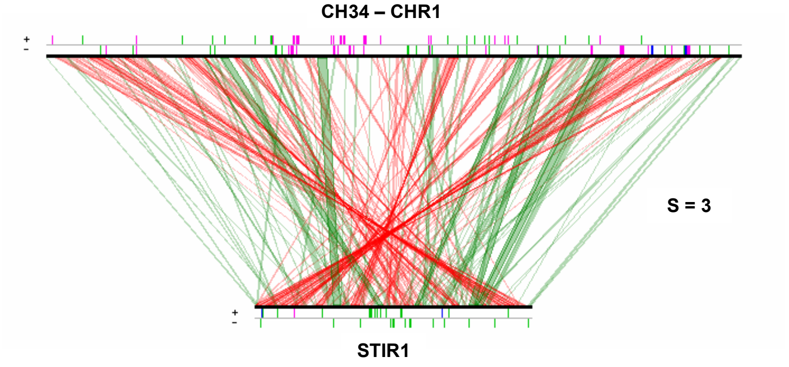

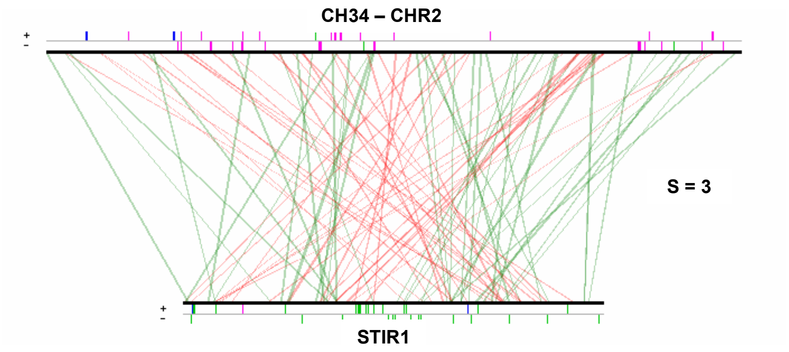

green and red lines depict strand conservations and strand inversions, respectively (S, synton size)

genes indicated on both strands (+/-) of the DNA:

pink = transposons (insertion sequences)
blue = rRNA
green = tRNA

<u>Fig. S4B</u>: PROmer analysis between CH34 (CHR1) and STIR1

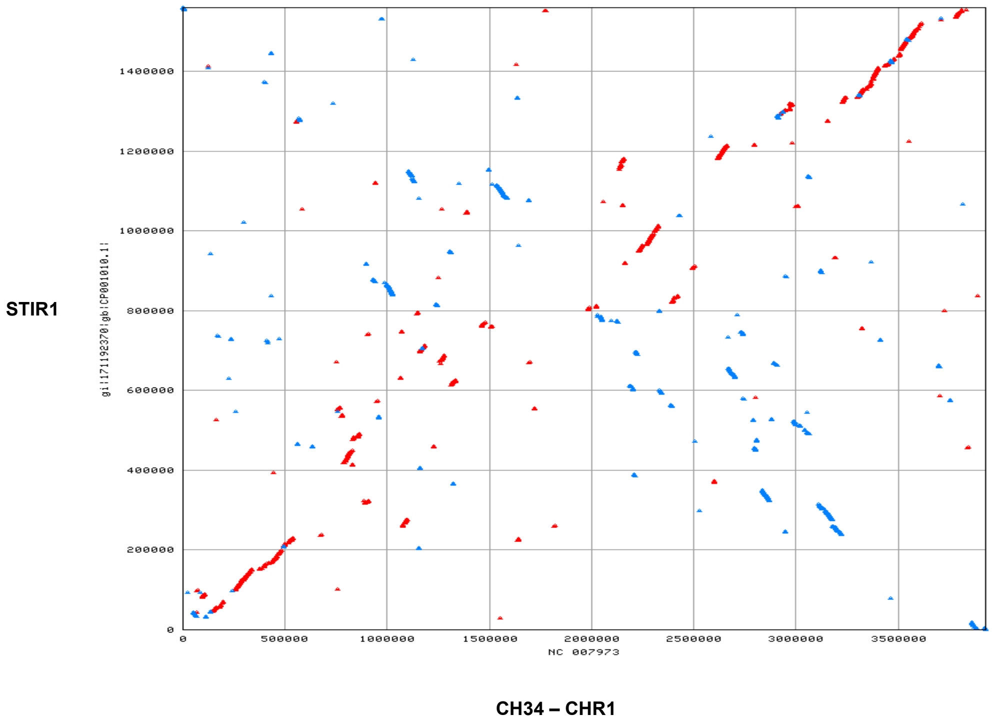

Supplement: Figure S4 — Protein-based synteny between the C. metallidurans CH34 chromosomes and the genome of P. necessarius STIR1, an obligate symbiont of freshwater protozoans. (A) Synteny displays were generated by the LinePlot tool of the MaGe system using a synton size (S) of 3 genes. Both species belong to the family of Burkholderiaceae and a plausible reason for the high synteny between CH34-CHR1 and the STIR1 genome would be the preservation of essential genes during genomic reduction in the STIR1 symbiont. Genes indicated on the positive (+) and negative (−) strand are rRNA (blue), tRNA (green), and transposons (pink). Strand conservations and strand inversions between de compared replicons are colored green and red, respectively. (B) Dot plot analysis of MUMs (maximal unique matching subsequences) between the main chromosome of C. metallidurans CH34 and the P. necessarius STIR1 genome. MUMs between both forward strands are shown in red and MUMs between the forward and reverse strands are shown in blue. The analysis was performed at protein level (using the six reading frames) deploying the PROmer module of MUMmer software with default parameters. (0.18 MB PDF) [file pone.0010433.s004.pdf]
