# Supplementary material for: The Complete Genome Sequence of Cupriavidus metallidurans Strain CH34, a Master Survivalist in Harsh and Anthropogenic Environments
Source: PLoS One. 2010 May 5;5(5):e10433. doi: 10.1371/journal.pone.0010433 (PMC2864759; doi:10.1371/journal.pone.0010433)

Figure S5. Pairwise Smith-Waterman alignments of RpoD protein sequences for all analysed genomes represented as a heatmap (gene notations from GenBank)

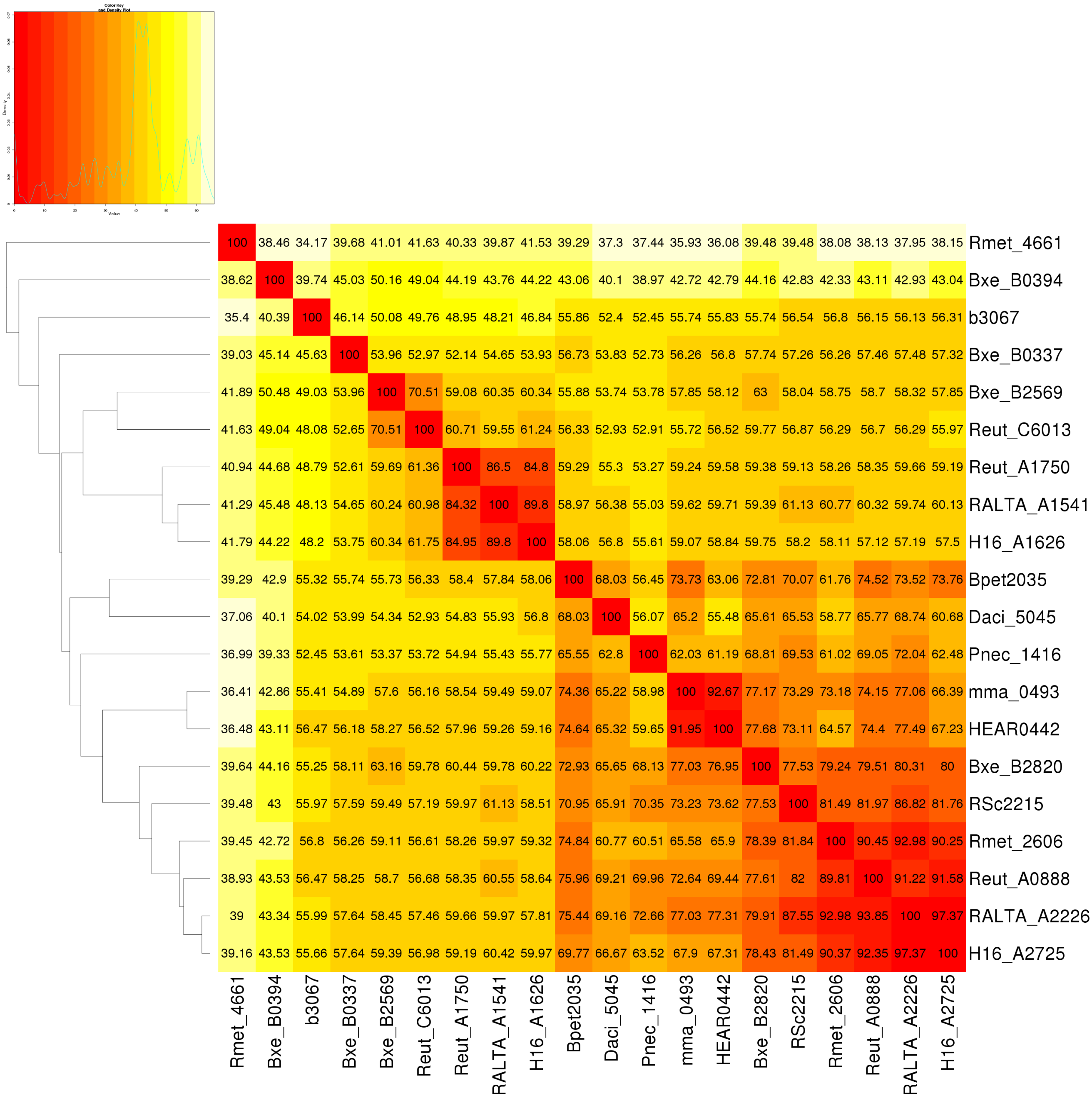

Supplement: Figure S5 — Sigma-70 sequence similarity heatmap based on pair-wise Smith-Waterman alignments of RpoD sequences. While the RpoD1 protein of CH34 (Rmet_2606 sited on CHR1) groups tightly with RpoD orthologs from the other three Cupriavidus species, the second sigma-70 factor RpoD2 (Rmet_4661 sited on CHR2) does not group with any of the RpoD orthologues in Cupriavidus but displays more significant BlastP hits with the RpoD proteins of Bordetella and Burkholderia species. All gene notations are from GenBank. Abbreviations: Rmet, C. metallidurans CH34; Bxe, B. xenovorans LB400; Reut, C. pinatubonensis JMP134; RALTA, C. taiwanensis; H16, C. eutrophus H16; Bpet, B. petrii; Daci, D. acidovorans SPH-1; Pnec, P. necessarius STIR1; mma, Janthinobacterium sp. Marseille; HEAR, H. arsenicoxydans; RSc, R. solanacearum GMI1000; b3067 denotes the RpoD protein sequence of E. coli str. K-12 substr. MG1655. (0.73 MB PDF) [file pone.0010433.s005.pdf]
