# Supplementary material for: The Complete Genome Sequence of Cupriavidus metallidurans Strain CH34, a Master Survivalist in Harsh and Anthropogenic Environments
Source: PLoS One. 2010 May 5;5(5):e10433. doi: 10.1371/journal.pone.0010433 (PMC2864759; doi:10.1371/journal.pone.0010433)
Supplement: Table S1 — Functional classification. Normalised distribution of functional content over the two large replicons of C. metallidurans CH34, based on COG assignments. These data were used for colorcoding in Figure 1 and the drawing of Figure 2. (0.08 MB PDF) [file pone.0010433.s009.pdf]

Class	Description	CHR1	CHR1	CHR2	CHR2	ratio	CHR1	CHR2
D	Cell cycle control, cell division, chromosome partitioning	50	1,33	10	0,41	3,28	35	4
M	Cell wall/membrane/envelope biogenesis	199	5,28	133	5,39	1,02	160	114
N	Cell motility	76	2,02	93	3,77	1,87	55	81
O	Posttranslational modification, protein turnover, chaperones	197	5,23	45	1,82	2,87	144	27
T	Signal transduction mechanisms	191	5,07	167	6,76	1,33	125	111
U	Intracellular trafficking, secretion, and vesicular transport	119	3,16	78	3,16	1,00	49	21
V	Defense mechanisms	101	2,68	54	2,19	1,23	48	29
A	RNA processing and modification	1	0,03	0	0,00	1	1	0
B	Chromatin structure and dynamics	4	0,11	0	0,00	1	3	0
J	Translation, ribosomal structure and biogenesis	199	5,28	24	0,97	5,44	176	22
K	Transcription	296	7,86	273	11,06	1,41	228	228
L	Replication, recombination and repair	221	5,87	87	3,52	1,67	187	78
C	Energy production and conversion	295	7,83	184	7,45	1,05	247	156
E	Amino acid transport and metabolism	515	13,67	278	11,26	1,21	409	226
F	Nucleotide transport and metabolism	86	2,28	31	1,26	1,82	64	16
G	Carbohydrate transport and metabolism	210	5,58	140	5,67	1,02	93	33
H	Coenzyme transport and metabolism	137	3,64	41	1,66	2,19	96	24
I	Lipid transport and metabolism	194	5,15	185	7,49	1,45	155	164
P	Inorganic ion transport and metabolism	330	8,76	192	7,78	1,13	147	84
Q	Secondary metabolites biosynthesis, transport and catabolism	132	3,51	144	5,83	1,66	71	75
R	General function prediction only	530	14,07	350	14,18	1,01	276	161
S	Function unknown	284	7,54	242	9,80	1,30	271	232
		(1)	(2)	(3)	(4)	(5)	(6)	(7)
	**nr. of CDS**	**3766**		**2469**			3040	1886

Table S1 - distribution of functional content over the two large replicons of *C. metallidurans* CH34

TheCDS numbers in columns (1) and (3) were extracted from COG tables obtained via the MaGe Export page and are in principal equal to the numbers obtained from the MaGe Genome Overview page (COG Automatic Classification). Classification into COGs is done by the COGnitor software imbedded into the MaGe system in which each protein is compared to the COG databank using BlastP. All best hits are scanned and the protein is classified in a COG if this protein has at least 3 Best Hits with proteins classified in the same COG but originating from 3 different taxonomic clades. As a consequence, proteins may be classified in more then one COG. Columns (2) and (4) are the percentages per replicon while column (5) is the ratio of these percentages. These ratios are used to draw Fig. 2 to display the bias towards particular COG classes. Colums (6) and (7) represent COG numbers taking only the highest COGnitor score into account (e.g. for colorcoding in Fig.1).
