# Supplementary material for: The Complete Genome Sequence of Cupriavidus metallidurans Strain CH34, a Master Survivalist in Harsh and Anthropogenic Environments
Source: PLoS One. 2010 May 5;5(5):e10433. doi: 10.1371/journal.pone.0010433 (PMC2864759; doi:10.1371/journal.pone.0010433)
Supplement: Table S6 — Functional distribution and chromosomal location of C. metallidurans CH34 genes implicated in iron uptake and -metabolism. (0.12 MB DOC) [file pone.0010433.s014.doc]

**Table S6**

| **Function** | **Replicon** | **Gene** | **Rmet_** | **Start** | **End** | **Annotation (product)** |
| --- | --- | --- | --- | --- | --- | --- |
| **Iron storage** | CHR1 | *bfr* | 0195 | 203746 | 204222 | bacterioferritin, iron storage and detoxification protein |
|  | CHR1 | *bfr* | 0248 | 264456 | 264935 | bacterioferritin, iron storage and detoxification protein |
| **Regulation** | CHR1 |  | 0128 | 134333 | 134842 | putative ferric uptake regulator, Fur-family |
|  | CHR1 |  | 1119 | 1222378 | 1223451 | FecR-like transmembrane sensor |
|  | CHR1 | *rpoI (fecI)* | 1120 | 1223515 | 1224015 | RNA polymerase sigma-19 factor |
|  | CHR1 | *fur* (*aleO)* | 2976 | 3235281 | 3235712 | transcriptional dual regulator of siderophore biosynthesis and transport |
|  | CHR2 | *fecR* | 4498 | 952517 | 953503 | anti FecI sigma factor, regulatory protein iron uptake |
|  | CHR2 | *fur* | 5746 | 2359972 | 2360445 | ferric uptake regulator, transcriptional regulator of siderophore biosynthesis |
| **Siderophore Synthesis** | CHR1 | *bfrC* | **1104** | 1201230 | 1203416 | hydroxamate/ferric/ferrichrome siderophore receptor (TonB-dependant) |
|  | CHR1 | *fcuA* | **1108** | 1206098 | 1208383 | ferric/ferrichrome receptor (TonB-dependant) |
|  | CHR1 |  | 1109 | 1208442 | 1208810 | conserved hypothetical protein |
|  | CHR1 | *lysA* | 1110 | 1208965 | 1210158 | diaminopimelate decarboxylase (DAP decarboxylase) |
|  | CHR1 |  | 1111 | 1210197 | 1210952 | aldolase |
|  | CHR1 | *iucC* | 1112 | 1210949 | 1212802 | putative aerobactin siderophore biosynthesis protein |
|  | CHR1 | *iucC* | 1113 | 1212808 | 1214634 | putative aerobactin siderophore synthetase component protein |
|  | CHR1 |  | 1114 | 1214621 | 1215808 | Permeases of the major facilitator superfamily (MFS) |
|  | CHR1 | *iucA* | 1115 | 1215811 | 1217580 | Putative siderophore synthetase component protein |
|  | CHR1 | *ocd* | 1116 | 1217577 | 1218644 | ornithine cyclodeaminase (OCD) |
|  | CHR1 | *cysK* | 1117 | 1218641 | 1219666 | cysteine synthase |
|  | CHR1 | *aleB* | **1118** | 1219766 | 1222285 | staphyloferrin B receptor |
|  | CHR1 |  | 1119 | 1222378 | 1223451 | FecR-like transmembrane sensor |
|  | CHR1 | *rpoI (fecI)* | 1120 | 1223515 | 1224015 | RNA polymerase sigma I (sigma 19) factor |
|  | CHR2 | *entE* | 3777 | 166051 | 167754 | 2,3-dihydroxybenzoate-AMP ligase, Enterobactin synthetase component E |
| **Ferrous iron transport** | CHR2 | *feoB* | 5890 | 2520783 | 2522645 | Fe2+ transport system protein B |
|  | CHR2 | *feoA* | 5891 | 2522669 | 2522971 | Fe2+ transport system protein A |
| **Siderophore Uptake** | CHR1 |  | **0123** | 128153 | 130312 | outer membrane receptor, TonB dependent |
|  | CHR1 | *exbD2* | 0536 | 570742 | 571173 | biopolymer transport protein |
|  | CHR1 | *exbB2* | 0537 | 571191 | 571811 | biopolymer transport channel protein |
|  | CHR1 |  | **0837** | 915139 | 917418 | hydroxamate-type ferrisiderophore receptor |
|  | CHR1 |  | **1819** | 1973011 | 1975239 | TonB-dependent siderophore receptor |
|  | CHR1 | *tonB* | 2277 | 2500813 | 2501472 | TonB-like protein, membrane spanning |
|  | CHR1 | *exbB1* | 2278 | 2501526 | 2502257 | biopolymer transport exbB protein |
|  | CHR1 | *exbD1* | 2279 | 2502290 | 2502703 | biopolymer transport protein |
|  | CHR1 | *btuB* | 2789 | 3035218 | 3037314 | outer membrane cobalamin receptor, TonB dependent |
|  | CHR1 |  | 3055 | 3311718 | 3312704 | putative TonB-like protein |
|  | CHR1 |  | **3077** | 3332074 | 3334335 | outer membrane receptor, TonB-dependent |
|  | CHR2 |  | **3999** | 422017 | 424482 | putative TonB-dependent siderophore receptor |
|  | CHR2 | *viuB* | 4199 | 636819 | 637631 | FAD-binding 9, siderophore-interacting protein |
|  | CHR2 | *ffcA2* | **4496** | 947754 | 950168 | ferric siderophore receptor protein |
|  | CHR2 | *ffcA1* | **4497** | 950179 | 952413 | ferric siderophore receptor protein,TonB-dependent |
|  | CHR2 |  | **4565** | 1028158 | 1030359 | TonB-dependent receptor |
|  | CHR2 | *oprC* | **4607** | 1085213 | 1087387 | TonB-dependent copper receptor |
|  | CHR2 | *piuA* | **4617** | 1095946 | 1098171 | hydroxamate-type ferrisiderophore receptor (PiuA-like) |
|  | CHR2 |  | **5373** | 1951113 | 1953098 | TonB-dependent outer-membrane transporter |
|  | CHR2 | *fecA1* | **5806** | 2418875 | 2421070 | ferric citrate outer membrane receptor |
|  | CHR2 | *fecA2* | **5807** | 2421332 | 2423485 | ferric citrate outer membrane receptor |

(ferric siderophore receptor genes with Rmet_ numbers in colored bold)
