# Supplementary material for: The Complete Genome Sequence of Cupriavidus metallidurans Strain CH34, a Master Survivalist in Harsh and Anthropogenic Environments
Source: PLoS One. 2010 May 5;5(5):e10433. doi: 10.1371/journal.pone.0010433 (PMC2864759; doi:10.1371/journal.pone.0010433)
Supplement: Table S9 — Functional distribution and chromosomal location of C. metallidurans CH34 genes involved in polyester (PHA/PHB) biosynthesis and degradation. (0.06 MB DOC) [file pone.0010433.s017.doc]

**Table S9.** poly-3-hydroxybutyrate (PHB) biosynthesis and degradation genes

| **Function** | **EC Number** | **Replicon** | **Gene** | **Rmet_**  **number** | **start** | **end** | **Annotation (product)** |
| --- | --- | --- | --- | --- | --- | --- | --- |
| **Acetoacetyl-CoA**  **reductase** | 1.1.1.36 | CHR1 | *phaB1* | 1358 | 1467642 | 1468382 | acetoacetyl-CoA reductase |
|  |  | CHR1 | *phaB2* | 1333 | 1444571 | 1445317 | acetoacetyl-CoA reductase |
|  |  | CHR2 | *phaB3* | 5123 | 1670972 | 1671718 | acetoacetyl-CoA reductase |
| **Poly**  **(3-hydroxybutyrate)**  **polymerase** | 2.3.1.- | CHR1 | *phaC1* | 1356 | 1464177 | 1466267 | Poly(3-hydroxybutyrate) polymerase |
|  |  | CHR1 | *phaC2* | 1671 | 1807369 | 1809087 | Poly(3-hydroxybutyrate) polymerase |
|  |  | CHR2 | *phaC3* | 5122 | 1669058 | 1670968 | Poly(3-hydroxybutyrate) polymerase |
| **Acetyl-CoA**  **acetyltransferase** | 2.3.1.9 | CHR1 | *phaA* | 1357 | 1466344 | 1467525 | Acetyl-CoA acetyltransferase (Acetoacetyl-CoA thiolase) |
|  |  | CHR1 | *atoB* | 1853 | 2007441 | 2008619 | Acetyl-CoA acetyltransferase (Acetoacetyl-CoA thiolase) |
|  |  | CHR1 | *atoB* | 106 | 113259 | 114443 | Acetyl-CoA acetyltransferase (Acetoacetyl-CoA thiolase) |
|  |  | CHR1 | *bktB*/*atoB* | 1362 | 1471529 | 1472713 | Acetyl-CoA acetyltransferase (Acetoacetyl-CoA thiolase) |
| **Regulation** | - | CHR1 | *phaR* | 1359 | 1468751 | 1469302 | Transcriptional regulator |
| **Phasin** | - | CHR1 | *phaP* | 1200 | 1319777 | 1320355 | Phasin (PHA-granule associated protein) |
| **Oligomer hydrolase** | - | CHR1 | *phaY1* | 1960 | 2135352 | 2137520 | D-(-)-3-hydroxybutyrate oligomer hydrolase |
| **Poly**  **(3-hydroxybutyrate)**  **depolymerase** | 3.1.1.75 | CHR2 | *phaZ* | 3823 | 218156 | 219280 | poly(3-hydroxybutyrate) (PHB) depolymerase |
|  |  | CHR1 | *phaZ1* | 1017 | 1106373 | 1107617 | intracellular poly(3-hydroxybutyrate) depolymerase |
|  |  | CHR1 | *phaZ2* | 2701 | 2934351 | 2935583 | intracellular poly(3-hydroxybutyrate) depolymerase |
|  |  | CHR2 | *phaZ3* | 3949 | 358779 | 359852 | poly(3-hydroxybutyrate) (PHB) depolymerase |
