# Supplementary material for: The Complete Genome Sequence of Cupriavidus metallidurans Strain CH34, a Master Survivalist in Harsh and Anthropogenic Environments
Source: PLoS One. 2010 May 5;5(5):e10433. doi: 10.1371/journal.pone.0010433 (PMC2864759; doi:10.1371/journal.pone.0010433)
Supplement: Table S10 — C. metallidurans CH34 genes for DNA repair and stress-related regulators. (0.08 MB DOC) [file pone.0010433.s018.doc]

**Table S10.** *C. metallidurans* CH34 genes identified for DNA repair and stress

| Base excision repair | |  | Direct repair pathway | |
| --- | --- | --- | --- | --- |
| Monofunctional DNA glycosylases | |  | *ada* | [Rmet_3765](https://www.genoscope.cns.fr/agc/mage/wwwpkgdb/Info/getInfoLabel.php?id=1512509&wwwpkgdb=6f3561a75f13c9d862ae224e276eab79) (CHR2) |
| *alkA* | [Rmet_3766](https://www.genoscope.cns.fr/agc/mage/wwwpkgdb/Info/getInfoLabel.php?id=1512510&wwwpkgdb=6f3561a75f13c9d862ae224e276eab79) (CHR2) |  | *alkB1* | [Rmet_3769](https://www.genoscope.cns.fr/agc/mage/wwwpkgdb/Info/getInfoLabel.php?id=1512513&wwwpkgdb=6f3561a75f13c9d862ae224e276eab79) (CHR2) |
| *mutY* | [Rmet_0295](https://www.genoscope.cns.fr/agc/mage/wwwpkgdb/Info/getInfoLabel.php?id=1514692&wwwpkgdb=6f3561a75f13c9d862ae224e276eab79) (CHR1) |  | *alkB2* | [Rmet_5268](https://www.genoscope.cns.fr/agc/mage/wwwpkgdb/Info/getInfoLabel.php?id=1512131&wwwpkgdb=6f3561a75f13c9d862ae224e276eab79) (CHR2) |
| *tag* | [Rmet_3404](https://www.genoscope.cns.fr/agc/mage/wwwpkgdb/Info/getInfoLabel.php?id=1516231&wwwpkgdb=6f3561a75f13c9d862ae224e276eab79) (CHR1) |  | *phr* | [Rmet_2745](https://www.genoscope.cns.fr/agc/mage/wwwpkgdb/Info/getInfoLabel.php?id=1515976&wwwpkgdb=6f3561a75f13c9d862ae224e276eab79) (CHR1) |
| *ung* | [Rmet_3183](https://www.genoscope.cns.fr/agc/mage/wwwpkgdb/Info/getInfoLabel.php?id=1516159&wwwpkgdb=6f3561a75f13c9d862ae224e276eab79) (CHR1) |  | *ogt1* | [Rmet_0520](https://www.genoscope.cns.fr/agc/mage/wwwpkgdb/Info/getInfoLabel.php?id=1516534&wwwpkgdb=6f3561a75f13c9d862ae224e276eab79) (CHR1) |
| Bifunctional DNA glycosylases | |  | *ogt2* | [Rmet_3767](https://www.genoscope.cns.fr/agc/mage/wwwpkgdb/Info/getInfoLabel.php?id=1512511&wwwpkgdb=6f3561a75f13c9d862ae224e276eab79) (CHR2) |
| *fpg* | Rmet_0293 (CHR1) |  | *ogt3* | [Rmet_5913](https://www.genoscope.cns.fr/agc/mage/wwwpkgdb/Info/getInfoLabel.php?id=1512447&wwwpkgdb=6f3561a75f13c9d862ae224e276eab79) (CHR2) |
| *nth* | Rmet_1014 (CHR1) |  |  |  |
| AP endonucleases | |  | Nucleotide excision repair | |
| *xthA2* | [Rmet_1189](https://www.genoscope.cns.fr/agc/mage/wwwpkgdb/Info/getInfoLabel.php?id=1516839&wwwpkgdb=6f3561a75f13c9d862ae224e276eab79) (CHR1) |  | *mfd* | [Rmet_1955](https://www.genoscope.cns.fr/agc/mage/wwwpkgdb/Info/getInfoLabel.php?id=1515593&wwwpkgdb=6f3561a75f13c9d862ae224e276eab79) (CHR1) |
| *xthA3* | [Rmet_4910](https://www.genoscope.cns.fr/agc/mage/wwwpkgdb/Info/getInfoLabel.php?id=1513071&wwwpkgdb=6f3561a75f13c9d862ae224e276eab79) (CHR2) |  | *uvrA1* | [Rmet_0313](https://www.genoscope.cns.fr/agc/mage/wwwpkgdb/Info/getInfoLabel.php?id=1516431&wwwpkgdb=6f3561a75f13c9d862ae224e276eab79) (CHR1) |
|  |  |  | *uvrA2* | Rmet_4549 (CHR2) |
| Recombinational repair | |  | *uvrB* | [Rmet_1019](https://www.genoscope.cns.fr/agc/mage/wwwpkgdb/Info/getInfoLabel.php?id=1515065&wwwpkgdb=6f3561a75f13c9d862ae224e276eab79) (CHR1) |
| *radA* | Rmet_1401 (CHR1) |  | *uvrC* | [Rmet_2410](https://www.genoscope.cns.fr/agc/mage/wwwpkgdb/Info/getInfoLabel.php?id=1517411&wwwpkgdb=6f3561a75f13c9d862ae224e276eab79) (CHR1) |
| *recA* | [Rmet_0466](https://www.genoscope.cns.fr/agc/mage/wwwpkgdb/Info/getInfoLabel.php?id=1514771&wwwpkgdb=6f3561a75f13c9d862ae224e276eab79) (CHR1) |  | *uvrD* | Rmet_2629 (CHR1) |
| *addA* | [Rmet_2131](https://www.genoscope.cns.fr/agc/mage/wwwpkgdb/Info/getInfoLabel.php?id=1517297&wwwpkgdb=6f3561a75f13c9d862ae224e276eab79) (CHR1) |  | *uvrD* | Rmet_6314 (pMOL28) |
| *addB* | [Rmet_2132](https://www.genoscope.cns.fr/agc/mage/wwwpkgdb/Info/getInfoLabel.php?id=1517298&wwwpkgdb=6f3561a75f13c9d862ae224e276eab79) (CHR1) |  | *uvrD2* | Rmet_6248 (pMOL28) |
| *recG* | Rmet_2942 (CHR1) |  | *uvrD* | Rmet_6048 (pMOL30) |
| *recJ* | Rmet_1038 (CHR1) |  |  |  |
| *recN* | Rmet_0998 (CHR1) |  | Mismatch repair pathway | |
| *recO* | Rmet_2416 (CHR1) |  | *dam* | [Rmet_5483](https://www.genoscope.cns.fr/agc/mage/wwwpkgdb/Info/getInfoLabel.php?id=1512244&wwwpkgdb=6f3561a75f13c9d862ae224e276eab79) (CHR2) |
| *recQ* | Rmet_3328 (CHR1) |  | *mutL* | [Rmet_2908](https://www.genoscope.cns.fr/agc/mage/wwwpkgdb/Info/getInfoLabel.php?id=1516043&wwwpkgdb=6f3561a75f13c9d862ae224e276eab79) (CHR1) |
| *recR* | Rmet_2127 (CHR1) |  | *mutS* | [Rmet_1075](https://www.genoscope.cns.fr/agc/mage/wwwpkgdb/Info/getInfoLabel.php?id=1516790&wwwpkgdb=6f3561a75f13c9d862ae224e276eab79) (CHR1) |
| *ruvA* | [Rmet_0424](https://www.genoscope.cns.fr/agc/mage/wwwpkgdb/Info/getInfoLabel.php?id=1516476&wwwpkgdb=6f3561a75f13c9d862ae224e276eab79) (CHR1) |  |  |  |
| *ruvB* | [Rmet_0423](https://www.genoscope.cns.fr/agc/mage/wwwpkgdb/Info/getInfoLabel.php?id=1516475&wwwpkgdb=6f3561a75f13c9d862ae224e276eab79) (CHR1) |  | Stress-related regulators | |
| *ruvC* | [Rmet_0426](https://www.genoscope.cns.fr/agc/mage/wwwpkgdb/Info/getInfoLabel.php?id=1516478&wwwpkgdb=6f3561a75f13c9d862ae224e276eab79) (CHR1) |  | *relA* | [Rmet_1159](https://www.genoscope.cns.fr/agc/mage/wwwpkgdb/Info/getInfoLabel.php?id=1515153&wwwpkgdb=6f3561a75f13c9d862ae224e276eab79) (CHR1) |
| *sbcC* | [Rmet_3664](https://www.genoscope.cns.fr/agc/mage/wwwpkgdb/Info/getInfoLabel.php?id=1511283&wwwpkgdb=6f3561a75f13c9d862ae224e276eab79) (CHR2) |  | *spoT* | [Rmet_0858](https://www.genoscope.cns.fr/agc/mage/wwwpkgdb/Info/getInfoLabel.php?id=1514966&wwwpkgdb=6f3561a75f13c9d862ae224e276eab79) (CHR1) |
| *sbcD* | [Rmet_3663](https://www.genoscope.cns.fr/agc/mage/wwwpkgdb/Info/getInfoLabel.php?id=1511282&wwwpkgdb=6f3561a75f13c9d862ae224e276eab79) (CHR2) |  | *lexA* | [Rmet_1981](https://www.genoscope.cns.fr/agc/mage/wwwpkgdb/Info/getInfoLabel.php?id=1515600&wwwpkgdb=6f3561a75f13c9d862ae224e276eab79) (CHR1) |
| *ssb1* | Rmet_0315 (CHR1) |  | *oxyR* | [Rmet_2941](https://www.genoscope.cns.fr/agc/mage/wwwpkgdb/Info/getInfoLabel.php?id=1517716&wwwpkgdb=6f3561a75f13c9d862ae224e276eab79) (CHR1) |
| *ssb2* | Rmet_2297 (CHR1, CMGI-1) |  | *soxR* | [Rmet_4538](https://www.genoscope.cns.fr/agc/mage/wwwpkgdb/Info/getInfoLabel.php?id=1512891&wwwpkgdb=6f3561a75f13c9d862ae224e276eab79) (CHR2) |
