# Supplementary material for: The Complete Genome Sequence of Cupriavidus metallidurans Strain CH34, a Master Survivalist in Harsh and Anthropogenic Environments
Source: PLoS One. 2010 May 5;5(5):e10433. doi: 10.1371/journal.pone.0010433 (PMC2864759; doi:10.1371/journal.pone.0010433)
Supplement: Table S11 — C. metallidurans CH34 genes encoding universal stress proteins (UspA superfamily) that are induced by a variety of starvation- and stress-conditions. (0.04 MB DOC) [file pone.0010433.s019.doc]

**Table S11.** Genes encoding for UspA family proteins in *C. metallidurans* CH34

| CHR1 | *uspA1* | Rmet_0458 |
| --- | --- | --- |
|  | *uspA2* | Rmet_1334 |
|  | *uspA3* | Rmet_1387 |
|  | *uspA4* | Rmet_1676 |
|  | *uspA5* | Rmet_1677 |
|  | *uspA6* | Rmet_1741 |
|  | *uspA7* | Rmet_2212 |
|  | *uspA8* | Rmet_3346 |
| CHR2 | *uspA9* | Rmet_4395 |
|  | *uspA10* | Rmet_4451 |
|  | *uspA11* | Rmet_4471 (on Tn*Cme3*) |
|  | *uspA11* | Rmet_4601 (on Tn*Cme3*) |
|  | *uspA12* | Rmet_4517 |
|  | *uspA13* | Rmet_4518 |
|  | *uspA14* | Rmet_4520 |
|  | *uspA15* | Rmet_5647 |
